# Refinement of cg05575921 demethylation response in nascent smoking

**DOI:** 10.1186/s13148-020-00882-w

**Published:** 2020-06-24

**Authors:** Kelsey Dawes, Allan Andersen, Emma Papworth, Brandon Hundley, Natasha Hutchens, Heba El Manawy, Ashley Becker, Luke Sampson, Willem Philibert, Frederick X. Gibbons, Meg Gerrard, Robert Philibert

**Affiliations:** 1 Department of Psychiatry, University of Iowa, Rm 2-426 MEB, 500 Newton Road, Iowa City, IA 52242 USA; 2 Molecular Medicine Program, University of Iowa, Iowa City, IA USA; 3 Department of Psychological Sciences, University of Connecticut, Storrs, CT USA

**Keywords:** Smoking, Cotinine, cg05575921, DNA methylation, ddPCR

## Abstract

The initiation of adolescent smoking is difficult to detect using carbon monoxide or cotinine assays. Previously, we and others have shown that the methylation of cg05575921 is an accurate predictor of adult smoking status. But the dose and time dependency of the demethylation response to smoking initiation in adolescents is not yet well understood. To this end, we conducted three consecutive annual in-person interviews and biological samplings of 448 high school students (wave 1 (W1)-wave 3 (W3)). At W1 (*n* = 448), 62 subjects reported using tobacco and 72 subjects reported using cannabis at least once in their life-time with 38 and 20 subjects having a positive cotinine and cannabinoid levels, respectively, at W1 intake. At W3 (*n* = 383), 67 subjects reported using tobacco and 60 subjects reported using cannabis at least once with 75 and 60 subjects having positive cotinine and cannabinoid levels, respectively, at W3. Subjects with undetectable cotinine levels at all three-time waves had stable levels of cg05575921 methylation throughout the study (88.7% at W1 and 88.8% at W3, *n* = 149), while subjects with positive cotinine levels at all 3 time points manifested a steady decrease in cg05575921 methylation (81.8% at W1 and 71.3% at the W3, *n* = 12). In those subjects with an affirmative smoking self-report at W3 (*n* = 17), the amount of demethylation at cg05575921 was correlated with time and intensity of smoking. We conclude that cg05575921 methylation is a sensitive, dose-dependent indicator of early stages of smoking, and may help to identify smokers in the early stages of smoking.

## Introduction

Smoking is the largest preventable cause of morbidity and mortality in the world [1]. Thankfully, public health measures including smoking prevention and early intervention have been at least partially effective in addressing this scourge with the latest surveys by the Centers for Disease Control indicating that the rate of adult smoking in the USA is now just 14% [2]. Still, each year, a substantial portion of youths begin to smoke. Since 90% of adult smokers report that they began smoking during their adolescence, prevention and treatment of adolescent smoking are both critical to further reductions of the adult smoking rate [3].

Like many addictive behaviors, smoking develops over an extended period of time. Typically, smoking begins with initial “experimental” puffs on a cigarette, which is then followed by episodic, context-dependent smoking [4]. Subsequently, regular contextual-independent smoking and then dependent smoking driven by psychological or physiological cues develop [4]. Since cessation efforts for dependent smokers have high failure rates, the optimal time for efforts may be during the irregular phase of smoking in adolescents during which some interventions appear to be particularly effective [5].

These early interventions for smoking may have other benefits including the reduction of risk behaviors that exhibit a co-morbidity with smoking. For example, early tobacco use is highly associated with early high-risk sexual behavior and drunk driving [6–8]. Although the exact temporal relationship needs to be further clarified, meta-analysis also supports a bi-directional relationship between early onset smoking and adolescent depression [9]. If this is correct, conceivably it should be possible to prevent or diminish the rate of these other co-morbid syndromes.

A barrier to the development of more effective early interventions for smoking and smoking related disorders maybe the method through which smoking behaviors are quantified. The default method for many clinicians for quantifying current smoking and the success of cessation therapy relies on patient self-reported data. Unfortunately, biological validation studies have shown that adolescents have high rates of unreliable self-report in both epidemiological and clinical treatment studies [10, 11]. This results in failure to recognize ineffective treatment and deprives patients of the opportunity to receive more intensive services when they fail to quit. Therefore, more objective methods for determining smoking status and program efficacy are needed to identify non-responders and prioritize them for more intensive efforts.

Currently, two biological methods are used to assess smoking status: exhaled carbon monoxide (CO) and cotinine levels [12]. While exhaled CO is the easiest method to perform, it has its limitations. Unfortunately, it is only able to detect smoking within the past 3-4 h, it is not sensitive to episodic or light smoking patterns, and thus is unable to quantify changes in smoking behaviors [12]. In contrast, cotinine has a half-life of 15 h and can detect nicotine use within the past 48-72 h. However, conventional cotinine determinations are unable to distinguish the source of nicotine exposure, creating false-positives when the subject uses nicotine replacement therapy. The development of a dose and time dependent biomarker that can quantify smoking behaviors could improve the outcomes of smoking prevention and treatment interventions.

Epigenetic biomarkers could be one solution to this problem. Dozens of genome wide studies have suggested that DNA methylation approaches may address some of these limitations [13, 14]. Building on these studies, we and others have shown that single assessments of blood or saliva DNA methylation status at cg05575921, a CpG residue in the aryl hydrocarbon repressor receptor (AHRR) locus, is extremely sensitive to regular adult smoking [15, 16]. Furthermore, in preliminary work, we have shown that saliva DNA assessment of cg05575921 predicts adolescent smoking status [17]. However, the sensitivity and timing of the demethylation response at cg05575921 to smoking initiation is not yet well defined. Here, we aimed to determine if clinicians could use the DNA methylations status of cg05575921 to detect the early stages of smoking initiation. In this communication, we further refine the early demethylation response and demonstrate that cg05575921 is sensitive to those in the escalating phase of smoking.

## Methods

### Study approval

All procedures and methods used in this study, including the NIH Certificate of Confidentiality and use of bilingual interview procedures, were approved by the University of Iowa Institutional Review Board (IRB 201409705).

### Study participants

The Healthy Iowans Study is a longitudinal study that follows high school sophomores over a 2-year period to better understand the trajectories of tobacco use in adolescence. The participants in this study were selected from seven public high schools in and around Johnson County Iowa. We obtained the publicly available contact information for the sophomores within that district. A letter describing the study was sent to each student and their parents. A research assistant then followed up with a phone call within 2-5 days of obtaining the letter to inquire about their interest in participating in the study. If interested, an intake appointment was scheduled for the student and at least one parent/guardian.

At the intake visit, a full and detailed description of the study was presented. Parents/guardians and the adolescents who were willing to participate in the study signed a written consent for their child, and themselves respectively. In the case of subjects whose Spanish-speaking parents were not fluent in English, written consent was obtained in Spanish from the parents by a bilingual staff member. If a student in the study reached the age of 18 over the course of the study, the student was re-consented. After consent, each student was interviewed with an abbreviated child version of the semi-structured assessment for the genetics of alcoholism in private by a trained research assistant (Supplementary File 1) at W1 and W3 [18]. The substance use questionnaire and the patient health questionnaire-9 (PHQ-9) were administered in person at W1, W2, and W3 (0 months, 12 months, and 24 months) [19, 20]. In addition, the substance use questionnaire and the PHQ-9 were administered over the phone at 6 month and 18 month time points. The substance use questionnaire is an inventory that counts the quantity consumed and delivery mode (e.g., cigarette vs cigar) of a variety of substances over 1 day, 1 week, 1 month, 6 month, and 12-month time periods. The PHQ-9 is a very well-established clinical aid that determines the presence and intensity of depressive symptoms. Phlebotomy was performed by a trained research assistant at 1-year intervals (W1, W2, and W3).

### Serological analyses

Sera was separated via centrifugation and stored at −80 °C until use. Serum cotinine and tetrahydrocannabinol (THC) levels were determined for all participants at each time point using quantitative cotinine and THC enzyme-linked immunoassay (ELISA) kits from AbNova (Taiwan) according to the manufacturer’s protocol.

### Exhaled CO assessment

Exhaled carbon monoxide (CO) was assessed using a Smokelyzer® according to manufacturer’s directions (CoVita, USA).

### DNA extraction and bisulfite conversion

Whole blood DNA was prepared using cold protein precipitation, quantified with a Nanodrop photometer (Thermofisher, USA) and stored at −20 °C until use [21]. The methylation status of cg05575921 was determined using a methylation sensitive droplet digital PCR as previously described [22]. First, 1 μg of whole blood DNA from each subject was bisulfite converted using the Fast 96 Bisulfite Conversion kit (Qiagen, Germany), then eluted in a 70 μl volume.

### DNA methylation quantification

The methylation status of cg05575921 was determined using a methylation sensitive droplet digital PCR as previously described [22]. In brief, a 3 μl aliquot of sample of bisulfite converted DNA was pre-amplified, diluted 1:3000, and then PCR amplified using fluorescent, dual labeled primer probe sets specific for cg05575921 from Behavioral Diagnostics (Coralville, IA) and Universal Digital PCR reagents and protocols from Bio-Rad (Carlsbad, CA). The QX-200 droplet counter and the Quantisoft Software (Bio-Rad, CA) were used to determine the number of droplets containing amplicons that have a “C” allele (representing a methylated cytosine residue), a “T” allele (representing an unmethylated cytosine), at least one “C” and “T” allele, or no amplified alleles. The results were calculated as a percent of methylation [22].

### Statistical analyses

All data were analyzed using JMP Version 10 (SAS Institute) using its embedded standard general linear model algorithms [23]. All data were analyzed using JMP Version 10 (SAS Institute) using its embedded standard general linear model algorithms [23]. *T* tests (*T* test) were used for comparisons between two groups with respect to continuous variables. One-way analysis of variance (ANOVA) was used for comparisons of groups with continuous variables and estimate the amount of variance explained by the predictor. Bivariate regression (bivariate) was used for analyses of the relationship between two continuous variables. Cotinine and carbon monoxide (CO) levels, depending on context, could be treated as either a categorical or a continuous variable. Cotinine levels of 2 ng/μl or greater were classified as categorical positives. Carbon monoxide levels of 10 parts per million (ppm) were treated as categorical positives. Self-report of substance use by subject or parent was treated as a categorical variable. Methylation and PHQ-9 scores were used as continuous variables.

## Results

The demographic and clinical characteristics of the 448 subjects who participated in the study are given in Table 1. At wave 1 (W1), the subjects averaged 15 years of age, were mostly of European ancestry (83%) and more likely to be female (55%) than male (45%). Although there was a sharp decrease in the number of subjects who participated from W1 (*n* = 448) to wave 2 (W2, *n* = 389), there was only a slight decrease in the number of subjects from W2 (*n* = 389) to wave 3 (W3, *n* = 383) with the proportions of gender and ethnicity remaining roughly constant in each wave.

**Table 1:**
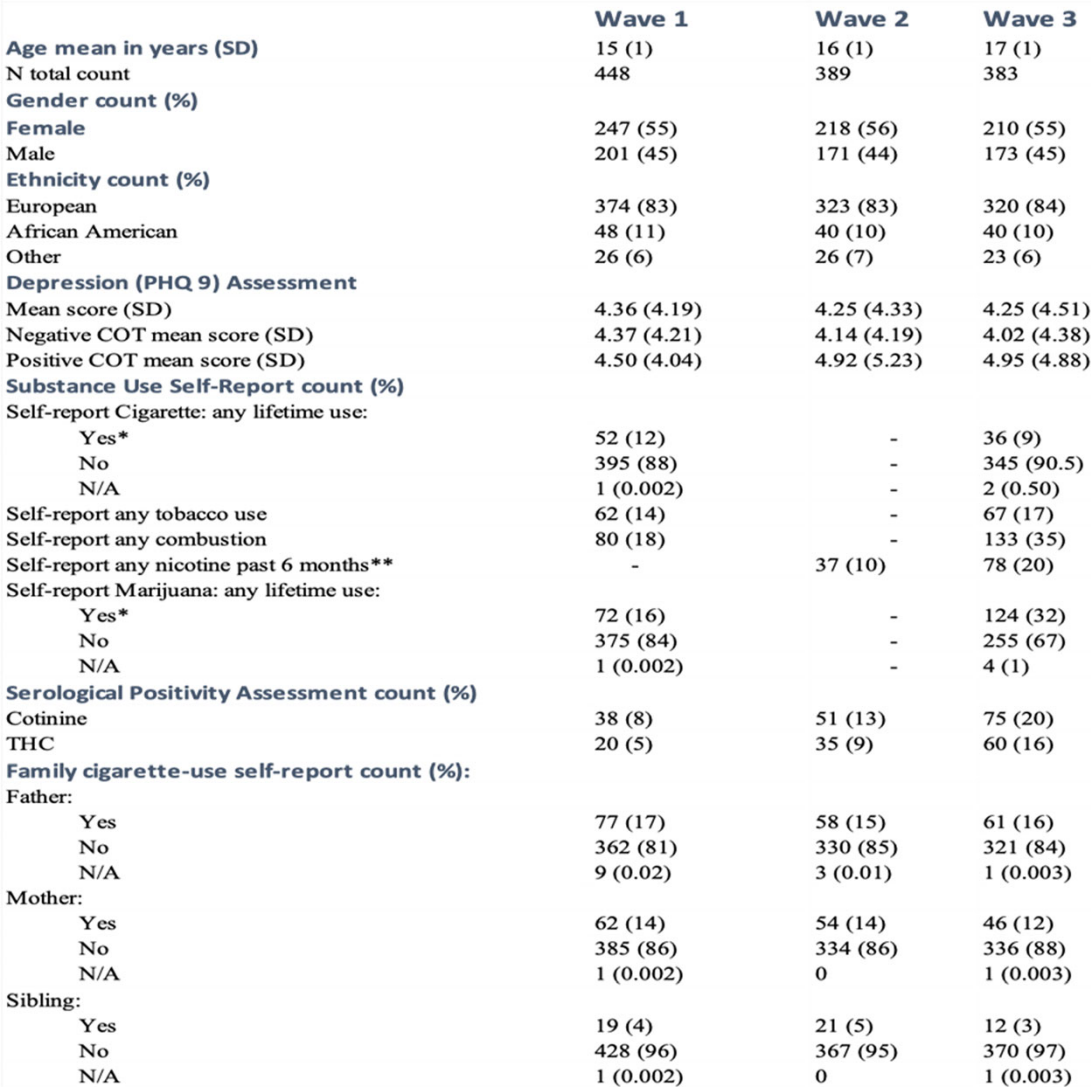
Demographic, clinical and substance use characteristics of subjects

*N/A* missing values

*22 of the 38 subjects that participated in W1 and W3, reported lifetime use of tobacco in W1 but denied use in W3; 10 of the 52 subjects that participated in both W1 and W3 reported lifetime marijuana use in W1 but denied use in W3

**Includes vaping/e-cigarette use

Each of the subjects was interviewed about their use of substances at each wave. In W1, 52 participants (12%) reported having smoked at least one puff of a cigarette in their lifetime. Interestingly, the number of subjects who answered affirmatively to the same question in W3 decreased to 36 participants (9%) with 22 of the 38 subjects who participated in both waves and giving an affirmative answer in W1, answering negatively to the same question in W3. The self-report of any tobacco use (including cigarettes, cigars, tobacco pipe, and chewing tobacco) within the past 6 months increased from 14% in W1 to 17% in W3. The self-report of any combustible tobacco or cannabis product (including cigarettes, any type of smoked cannabis, hookah, and tobacco pipe) within the past 6 months almost doubled from 18% in W1 to 35% in W3. The rate of self-reported nicotine use (including cigarettes, tobacco pipes, hookah, chewing tobacco, and e-cigarettes) in the past 6 months doubled from 10% in W2 to 20% in W3.

The self-report of any lifetime use of cannabis doubled from W1 (16%) to (33%) W3. Notably, contrast to the inconsistent self-report of lifetime cigarette use, only 10 of the 52 subjects who participated in both W1 and W3 and answered affirmatively to cannabis use in W1, denied lifetime cannabis use in W3.

The rate of cotinine positivity, defined by a serum level of 2 ng/ml or greater, increased at each wave (8%, 13%, and 20% respectively). In the same subjects, the presence of cotinine positivity at W1 was a strong predictor of cotinine positivity at subsequent timepoints; with 15 of the 25 subjects, who were positive for cotinine W1 and participated in W3, being positive for cotinine at wave 3. Similarly, the rate of detectable THC in serum increased steadily 4% at W1 to 9% at W2 to 16% at W3. Serum positivity for THC at earlier wave was also a strong predictor of serum positivity at a later wave. Seven of the 13 subjects who participated in both waves and were positive for THC at W1 were also positive for THC at wave 3.

**Fig. 1:**
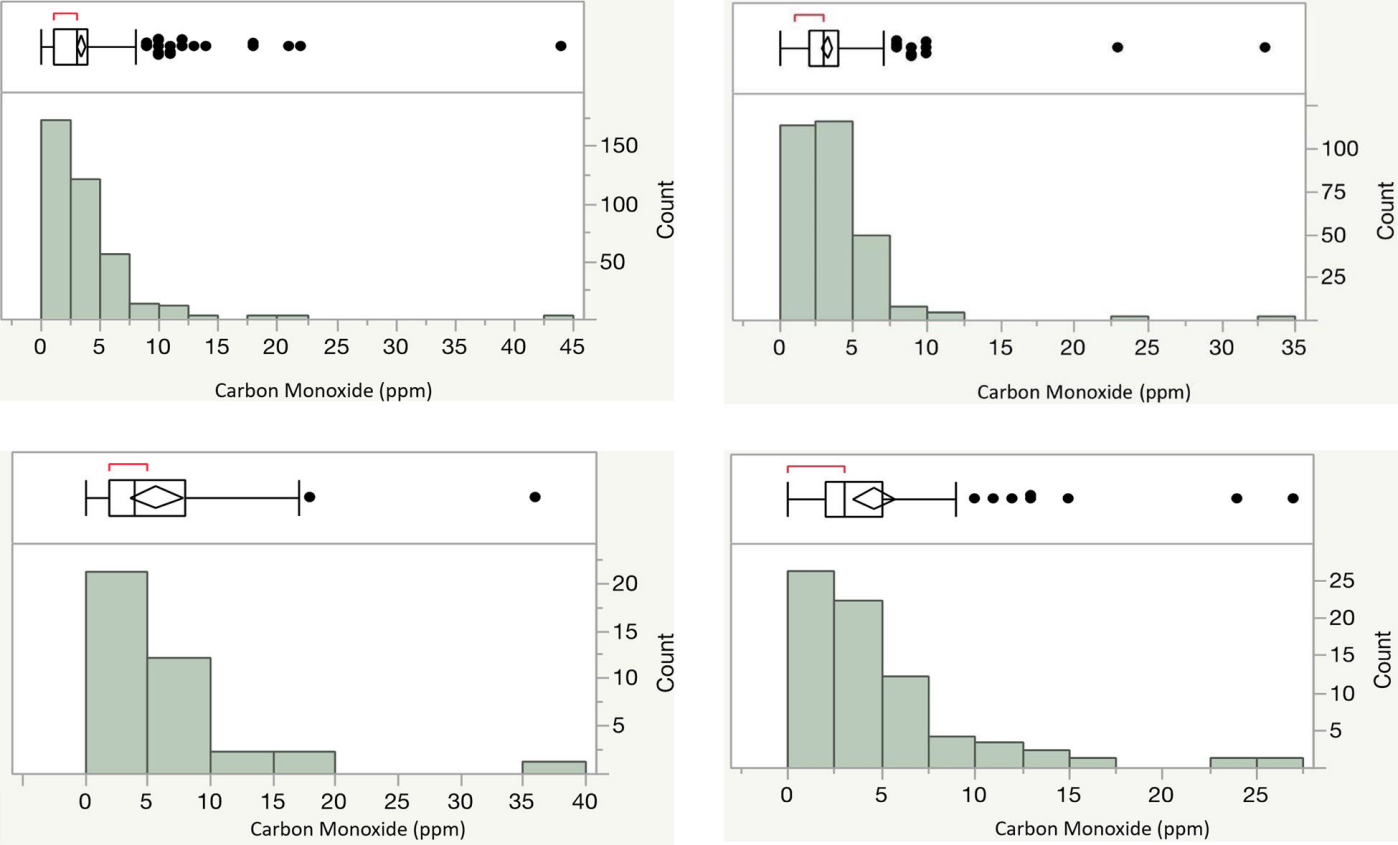
**a** CO levels in W1 cotinine negative subjects. Exhaled carbon monoxide levels (parts per millions; ppm) given on the *x*-axis. The box plot with bars indicating mean or standard deviation with individual data distribution outside of the 2nd SD indicated by filled circles is given above the histogram. The count of subjects with a particular CO level is given on *y*-axis. Each of these subjects in W1 had a negative cotinine level (defined as < 2 ng/mL), suggesting no nicotine use within the past 48 h (*n* = 329). **b** CO levels in W3 cotinine negative subjects. Exhaled carbon monoxide levels (parts per million; ppm) given on the *x*-axis. The box plot with bars indicating mean or standard deviation with individual data distribution outside of the 2nd SD indicated by filled circles is given above the histogram. The count of subjects with a particular CO level is given on *y*-axis. Each of these subjects in W3 had a negative cotinine level (defined as < 2 ng/mL), suggesting no nicotine use within the past 48 h (*n* = 257). **c** CO levels in W1 cotinine positive subjects. Exhaled carbon monoxide levels (parts per million; ppm) given on the *x*-axis. The box plot with bars indicating mean or standard deviation with individual data distribution outside of the 2nd SD indicated by filled circles is given above the histogram. The count of subjects with a particular CO level is given on *y*-axis. Each of these subjects in W1 had a positive cotinine level (defined as > 2 ng/mL), suggesting nicotine use within the past 48 h (*n* = 38). **d** CO levels in W3 cotinine positive subjects. Exhaled carbon monoxide levels (parts per million; ppm) given on the *x*-axis. The box plot with bars indicating mean or standard deviation with individual data distribution outside of the 2nd SD indicated by filled circles is given above the histogram. The count of subjects with a particular CO level is given on *y*-axis. Each of these subjects in W3 had a positive cotinine level (defined as > 2 ng/mL), suggesting nicotine use within the past 48 h (*n* = 75)

The level of exhaled CO (ppm) was measured at each wave (Fig. 1). As per the Smokerlyzer® manual’s guidelines, CO positivity is defined as ≥ 10 ppm and CO negativity as ≤ 10 ppm. At W1, 14 out of 329 subjects with negative serum cotinine values were CO positive while 33 out of 38 serum positive subjects were CO negative (Fig. 1a and b). Still, W1 categorical serum cotinine positivity (*R*^2^ = 0.03, *p* < 0.001, ANOVA) and absolute serum cotinine levels (*R*^2^ = 0.12, *p* < 0.0001, bivariate) significantly predicted CO levels. The absolute serum cotinine levels at W1 ranged from 0 to 156 ng/ml, with an average of 1.98 ng/ml. At W3 using the same 10 ppm cutoff, 4 out of 257 serum negative subjects were CO positive while 67 out of 75 cotinine positive subjects were CO negative (Fig. 1c and d). W3 categorical serum cotinine positivity (*R*^2^ = 0.02, *p* < 0.003, ANOVA) and absolute serum cotinine levels (*R*^2^ = 0.10, *p* < 0.0001, bivariate) also significantly predicted CO levels. The absolute serum cotinine levels at W3 ranged from 0 to 160.5 ng/ml, with an average of 8 ng/ml.

The level of depression at each wave was assessed with the patient depression questionnaire (PHQ-9). The severity of depression is associated with the total PHQ-9 score; a score of 1-4 is associated with minimal depression, 10-14 with moderate depression, and 20-27 with severe depression [19]. The average total PHQ-9 scores of all subjects were similar through all three waves (4.4 W1, and 4.3 in both W2 and W3).

Because others have suggested an etiological link between tobacco or cannabis use and depression, we analyzed the relationship of nicotine and cannabis use status to total PHQ-9 score. A positive cotinine value at W1 was not significantly correlated with the PHQ-9 score at W1 or at subsequent waves. A positive cannabinoid at W1 also was not significantly correlated with the PHQ-9 score at W1 or subsequent waves. Finally, although the relationship between W3 cotinine positivity and W3 PHQ-9 score was not significant, there was a trend for association between W3 THC positivity and W3 PHQ-9 score (*p* < 0.08).

An important emphasis of this study was to determine the reliability of self-report of tobacco and cannabis consumption in adolescents. The results of self-report as a function of ELISA positivity are given in Table 2. In W1, 25 of the 38 (66%) subjects who were positive for cotinine (serum level ≥ 2 ng/ml) denied the consumption of any nicotine containing product in the prior 6 months. The rate of unreliable report rose to 82% at W2 (37 out of 45) decreasing only slight to 77% (58 out of 75) at wave 3. In contrast, the frequency of unreliable self-report of cannabis consumption was substantially lower at the two time points in which its self-report of use was assessed with rates of only 32% (6 of 19, *p* < 0.02, bivariate regression) and 27% (16 of 60, *p* < 0.001) subjects at W1 and W3, respectively.

**Table 2:** Self-report as a function of positive ELISA

	W1	W2	W3
Positive cotinine			
Denied smoking	25	37	58
Reported smoking	13	8	17
Denied nicotine use	N/A	34	39
Reported nicotine use	N/A	17	33
Positive cannabinoid			
Denied cannabis use	6	N/A	16
Reported cannabis use	13	N/A	44

A major rationale for the conduct of this project was to understand the relationship between smoking and methylation status. Therefore, we examined the relationship of cotinine serum positivity or levels on cg05575921 methylation. At W1 both categorical use status (*R*^2^ = 0.06, *p* < 0.0001, ANOVA), and quantitative levels of cotinine (*R*^2^ = 0.30, *p* < 0.0001, bivariate) were correlated with cg05575921 status. At W1, in those who denied smoking, there were no significant effects of ethnicity, gender, or maternal smoking status on methylation levels. At W2, both categorical use status (*R*^2^ = 0.11, *p* < 0.0001, ANOVA), and quantitative levels of cotinine (*R*^2^ = 0.27, *p* < 0.0001, bivariate) were correlated with cg05575921 status. Finally, at W3, both categorical use status (*R*^2^ = 0.09, *p* < 0.0001, ANOVA) and quantitative levels of cotinine (*R*^2^ = 0.16, *p* < 0.0001, bivariate) were correlated with cg05575921 status.

Because smoking cannabis also generates polycystic aromatic hydrocarbons (PAH) [24], we next analyzed the relationship of both cotinine and THC serum positivity to methylation status. The addition of THC positivity to cotinine positivity modestly increased the amount of variance in the correlation of categorical use status (COT and THC) with methylation at W1 (*R*^2^ = 0.09, *p* < 0.0001, ANOVA), W2 (*R*^2^ = 0.13, *p* < 0.0001, ANOVA) and W3 (*R*^2^ = 0.10, *p* < 0.0001, ANOVA).

The high rate of unreliable self-report and the episodic initiation of nicotine use suggest the possibility that many of the subjects negative for serum cotinine yet positive for cotinine at a later time point were in fact smoking or vaping during earlier time points. To decrease the effects of these unreliable reports on the methylation trajectory and better understand the possible sources of the cotinine that was detected, we categorized subjects with respect to reliability of self-report and then re-examined the relationship between smoking and whole blood DNA methylation. In W1, 327 of the subjects (73%) gave reliable negative self-report while at W3, only 192 (49%) of the subjects denied prior nicotine use and had negative serum cotinine values. The average methylation levels of these reliable non-nicotine-use self-reporters were similar at each time point (W1, 86.7 ± 3.2 and W3, 87.2 ± 2.8). In contrast, those reliable reporters of smoking (i.e., subjects who both reported smoking and were positive for cotinine) had lower average levels of cg05575921 methylation (W1, 78.5 ± 11.3 and W3, 80.5 ± 12.1; *T* test, *p* < 0.001 for both). Finally, those unreliable self-reporters (i.e., subjects who denied nicotine use and yet were positive for cotinine) had average methylation levels arithmetically between those of the smokers and non-smokers at W1 (85.6% ± 3.8) and W3 (83.5% ± 8.0).

When interpreting these results, it is important to consider other potential sources of nicotine exposure. Only a handful of subjects reported the use of non-combustible forms of tobacco during the study. In contrast, the rates of self-reported e-cigarettes/vaping steadily increased each year with positive reports of use from 3 subjects in W1, 19 subjects in W2, and then 51 subjects in W3.

**Fig. 2:**
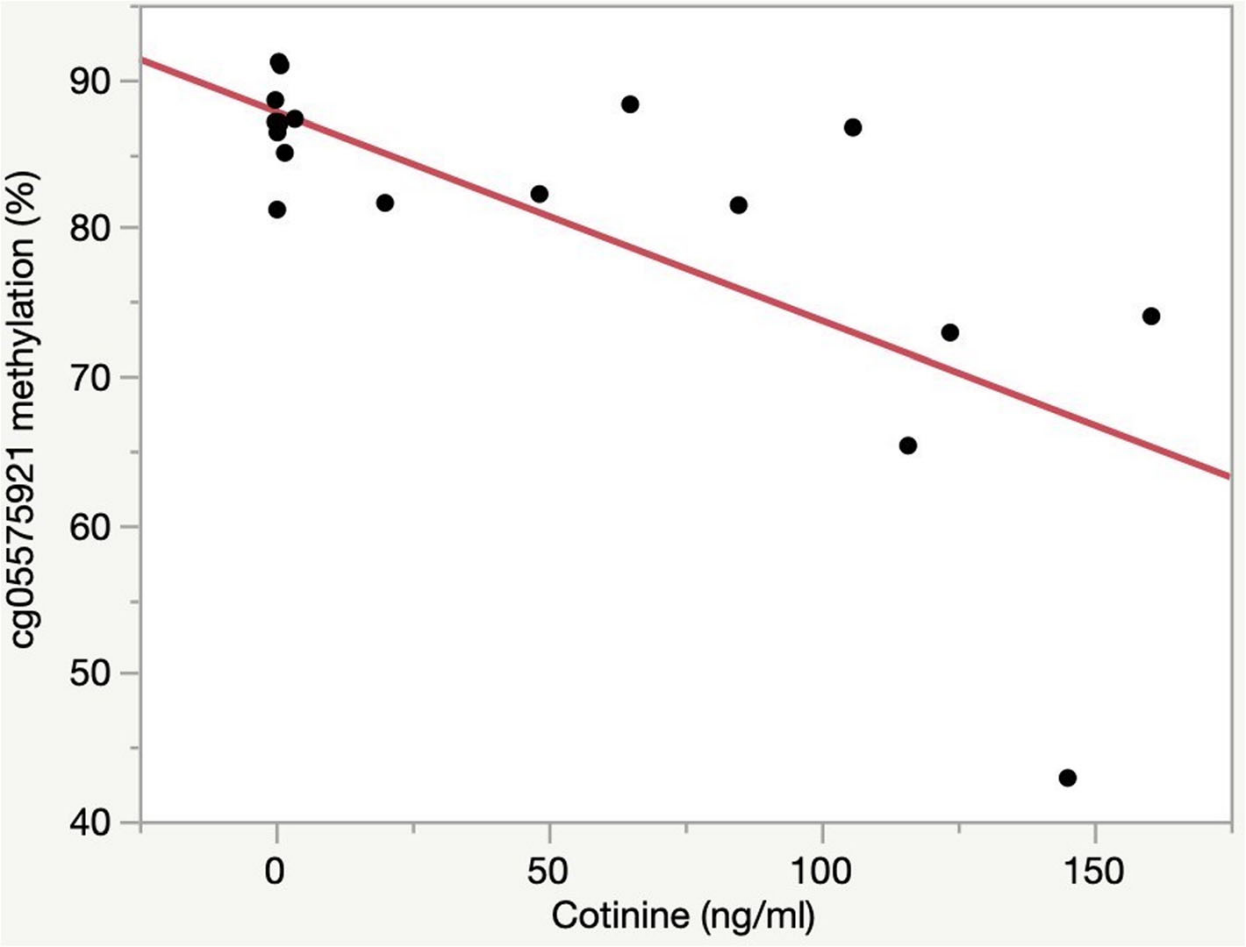
cg05575921 methylation as a function of cotinine levels in those with a positive cotinine and self-reported smoking in W3. Percent methylation, indicated by methylation-sensitive droplet digital PCR, is given on the *y*-axis. Cotinine value in ng/mL, indicated by enzyme-linked immunoassay, is given on the *x*-axis. Each subject had a positive cotinine (defined as > 2 ng/mL) and self-reported smoking cigarettes within the past 6 months (*n* = 17)

The single time point relationship between cotinine levels and cg05575921 levels in the self-reported W3 smokers is shown in Fig. 2. Using a linear model, we show that cg05575921 methylation levels are strongly negatively correlated with serum cotinine values (*p* < 0.001) which suggests that as daily cigarette consumption increases, methylation steadily decreases. There was not ethnicity or gender effect on the model.

**Fig. 3:**
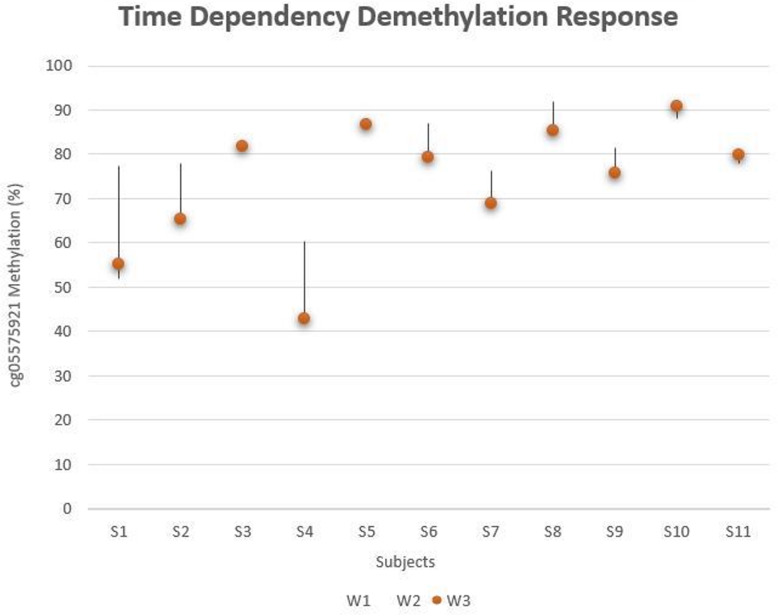
cg05575921 methylation in those with a positive cotinine at all three waves. Percentage of cg05575921, indicated by methylation-sensitive PCR, is given on the *y*-axis. Individual subjects characterized by a positive cotinine level at all three waves is given on the *x*-axis (*n* = 11). Subjects 1-5 self-reported using a combustible form of nicotine use. W1 is indicated at the top of each line, W3 is indicated by the orange dot, and the position of W2 is dependent on its methylation compared to W1 and W3

Eleven subjects, including 5 who reported smoking, had detectable levels of cotinine at each biological sampling (W1, W2, and W3). Figure 3 is a “tear-drop” plot of their methylation values at each time point. Although for 4 of the subjects, methylation values were relatively consistent over the three waves, seven of the subjects showed a marked decrease in methylation as a function of continued serum positivity. These decreases were most notable in subject S1, S2, and S4, all of whom were self-reported smokers.

## Discussion

In this longitudinal study, we demonstrate that cg05575921 demethylates in response to smoking in adolescents. For all three waves, the tobacco use status (defined as serum cotinine ≥ 2 ng/mL) and quantitative cotinine levels were significantly negatively correlated with methylation of cg05575921. Most notably, using data from the 19 subjects in W3 whom reported smoking cigarettes within the past week and had detectable cotinine values, we showed a negative relationship between quantitative cotinine levels and the degree of demethylation of cg05575921. Limitations of the findings include the high rate of unreliable self-report observed in the study, heterogeneity in the type of tobacco product consumed, and the single site, largely European ancestry cohort.

The high rate of unreliable self-report of nicotine use is not only consistent with some prior studies but also is informative for prospective study design. In this study, whose overarching goal was to understand the relationship between smoking and demethylation, 67% (W2) and 55% (W3) of the subjects positive for serum cotinine denied any use of nicotine containing products in the past 6 months. Since the bogus pipeline principle stipulates that telling subjects that they will be tested for substances should decrease the rate of unreliable self-report, we expected that informing each subject during the consenting procedures that we would be using testing for tobacco and cannabinoid metabolites; and testing for exhaled carbon monoxide levels at each phlebotomy visit would increase the level of reliable self-report [25, 26]. Nevertheless, we had a high, but not entirely unprecedented, rate of unreliable self-report. Using data from NHANES population collected over a decade ago, Caraballo and associates found that about 1/3 of the adolescents (12-17 years old) with serum cotinine levels > 10 ng/ml self-reported smoking [10]. Conceivably, if they would have used a lower cutoff level, even higher levels would have been observed. Taking the low cutoff into consideration, some of the false positives that we observed could have come from second hand smoke. In that regard, others have reported high rates of low background nicotine exposure in adolescents [27, 28]. It is also important to appreciate that many students who are vaping may not know that they were consuming nicotine. At the same time, we will note that among those in our study with positive serum levels, there is no relationship between the level of cotinine and veracity of self-report, which suggests that second hand smoke is not causing the majority of these discrepancies. Furthermore, in those with positive levels in earlier waves, there is a marked tendency for their cotinine levels to increase in subsequent waves which suggests that what we are detecting in those subjects is the progressive onset of a nicotine use disorder. Finally, we have not had a problem with false positives when using this ELISA methodology in our studies of adults. Therefore, we feel confident that the vast majority of the positive cotinine values that we observed are secondary to nicotine consumption by the subject. Still, no matter what explanation(s) for the high rate of unreliable self-report is correct, our findings suggest that projects such as ours whose goal is to understand the initial response thresholds of biomarker response in adolescents, may have difficulty obtaining accurate initial self-report of substance consumption.

The finding that the subjects were much more forthcoming with their self-report of cannabis use than their use of nicotine products was surprising. In contrast to the problems encountered with respect to nicotine use self-report, only 30% (W1) and 27% (W3), of those subjects with a positive cannabinoid level denied use of cannabis. Conceivably, this better rate of cannabis self-report could be due to shifting attitudes. Over the past decade, the use of marijuana for medical purposes has become progressively more socially acceptable. Conversely, the messaging with regards to nicotine use and, in particular, to smoking, has continued to increase in negativity. In a 2016 study of 786 California teenagers, Roditis and colleagues found that cannabis was consistently more positively viewed than cigarettes [29]. Unfortunately, these more positive attitudes towards cannabis may be misplaced [30] and our findings support the need for additional steps to educate adolescents on the potential harms of cannabis use [31].

These data suggest that the demethylation response to PAH occurs early in the course of smoking. In prior work in the Strong and Healthy African Americans Project (SHAPE) cohort, we showed clear demethylation at cg05575921 in a group of 42 eighteen-year-old subjects who reported less than a ½ pack-year of consumption (3600 cigarettes) [32]. The objective and subjective data from the current study support those prior findings. With respect to a purely objective approach, a simple linear fit of the objective data from the nineteen W3 subjects who reported some smoking in 6 months prior to phlebotomy shows that a serum cotinine level of 100 ng/ml corresponds to a methylation level of about 72%. According to the data of Caraballo and associates, this level of cotinine reflects the consumption of about 4 cigarettes per day. Assuming that these subjects smoked at that rate for a year, this would correspond to an annual average consumption of 0.2 pack years for those 5 subjects with cotinine levels ≥ 100 ng/ml. Still, the actual consumption of cigarettes that these cotinine levels represent may be lower. In our studies of adults who only smoked cigarettes that used this same ELISA assay, the same methylation level corresponded to ~ 2 cigarettes per day [15]. Furthermore, the majority of the 19 W3 subjects, whose data is given in Fig. 1, also reported using other sources of nicotine, it is likely that a substantial portion of the cotinine in these subjects may be from non-combustible sources. Subjectively, the number of cigarettes reported by those subjects was remarkably low. All totaled together, the 19 subjects report a combined consumption of less than 3400 cigarettes (n.b., 1 pack year = 7300 cigarettes in a year) over the 3 years prior to the W3 blood draw. If that figure is even remotely reflective of actual cigarette consumption that indicates that the actual threshold for demethylation may be relatively small, as little as a carton of cigarettes for some individuals. Given the challenges that we experienced obtaining reliable information, resolving the exact threshold for demethylation in adolescents may be difficult and thus achieving an accurate understanding of the relative effects of acute versus chronic smoke exposure on demethylation in adolescents may be impossible. If this is so, establishing an exact understanding of the dose and time dependency of initial demethylation may be a task best accomplished through use of a rodent or primate model.

An important finding of this study is that in the absence of smoking, the baseline methylation of subjects of both genders and all ethnicities at study entry (age ~ 15 years) is about the same (86.7% ± 3.2). This may be confusing to some because of Joubert and colleague’s prior demonstration that the newborns from mothers who smoked had lower methylation at cg05575921 [33]. However, we and others have shown that methylation at cg05575921 reverts as a function of smoking cessation [34–37]. Bauer has shown that in the absence of smoking, the smoking induced methylation changes at this locus in newborns fully reverts back to normal within 2 years [36]. The reversion speed in adults may be even faster. In 2016, we showed in a small group of subjects that the methylation levels at cg05575921 increased ~ 5% after 1 month of smoking cessation. Recently, we have confirmed and extended this finding in a larger set of subjects [38].

Our findings also support some prior studies that suggest that CO detectors are limited tools for screening for smoking in adolescent or adult smokers [39, 40]. In our study of smoking cessation in adults, we found that the same model of CO detector used in this study gave reading of 8 ppm or more in less than ½ of the subjects who smoked 5 or less cigarettes per day [34]. Hence, if those data are correct, using the default adult cutoff for CO in adult smokers [41], it is unlikely that the CO monitor would detect smokers in the earliest phases of smoking where smokers consume only 1 or two cigarettes per day. If a lower cutoff such as the 3 ppm suggested by Javors and colleagues is used, [42] many more smokers would be detected. But the rate of false positive detections would rise dramatically. At W3, 100 of the 287 cotinine negative subjects had readings of 4 ppm or greater. Therefore, we do not believe that altering the cutoff point is a viable solution for the use of this CO monitor in our hands.

However, we may never know the true utility of CO monitors or methylation tests in adolescents due of the challenges imposed by the increasing use of e-cigarettes or vape pens. In brief, to understand the sensitivity and specificity of a given device, one must have a gold standard for the trait being examined. In the past, cotinine determinations served as a gold standard for smoking. However, vaping produces much lower levels of PAH and CO than conventional cigarettes [43–45] and the rate of self-reported use of e-cigarettes/vape pens markedly increased from W1 to W3 in our population and in the nation overall during the time this study was conducted (2015-2018) [46]. It should also be noted that adolescents often do not know the content of the solutions that they are vaping. Because of these developments, cotinine determinations can no longer be considered as a gold standard for smoking in adolescents and we believe that many of the positive cotinine levels detected during this study do not reflect the use of combustible sources of nicotine. Therefore, many of the “positive cotinines” that we observed may not represent smoking and therefore could not be detected by a CO monitor or the methylation test.

These data also highlight the tendency of prolonged use for both nicotine and cannabis. Sixty percent (15 of 25) of the subjects who had a positive cotinine value at W1 and participated in W3, also had a positive cotinine value at W3. Similarly, 54% of the 13 subjects who had a positive cannabinoid level at W1 and participated in W3 had a positive W3 cannabinoid level. These data make it even more clear that once use of either of these substances has been initiated, it is likely to continue which highlights the need for prevention of initiation.

A large number of studies have examined the relationship between depression and substance use initiation [47]. Consistent with some but in contrast to others [48–51], our results show a modest yet significant relationship between depression and the later onset of both nicotine and cannabis use, but not the vice versa. However, the failure to show a relationship between smoking or cannabis consumption and the later onset of depression does not mean a relationship does not exist. Because there were only 38 subjects positive for cotinine and 20 cannabinoids at W1 power to detect an effect is rather limited. Instead, what our results highlight are the complex yet significant interrelationships between depression and substance initiation and the need to be vigilant for both of these highly prevalent conditions in adolescents.

Another unresolved issue is the relationship of vaping to the initiation of smoking. Conceivably, by testing the serum samples for combusted forms of tobacco metabolites such as 2-cyanoethylmercapturic acid (CEMA) or looking for non-nicotine-derived tobacco metabolites, we could gather additional insight as to which positive cotinine values result from tobacco use and which are secondary to vaping [52]. However, urine is the preferred substrate for this type of mass spectroscopy testing and it should be noted that even this cumbersome and expensive form of testing is not particularly sensitive to tobacco use in the early stages of smoking. The unreliability of self-report of nicotine initiation in this age group will pose a formidable challenge to those seeking to understand the initiation of nicotine use at its earliest stages. In future studies, these limitations could be overcome using a battery of biological measures that include metabolite testing and epigenetic measures, minimizing the effects of unreliable self-report on the development of a more comprehensive understanding of the relationship of vaping to the onset of smoking.

In conclusion, we demonstrated that demethylation of cg05575921 occurs early in the trajectory of smoking and that it may serve as a useful tool for understanding factors associated with the initiation or maintenance of smoking behaviors.

## Supplementary information

**Additional file 1.** Abbreviated child version of the Semi-structured Assessment for the Genetics of Alcoholism.

## Data Availability

The data that support the findings of this study are available from Dr. Robert Philibert, Robert-philibert@uiowa.edu, but restrictions apply to the availability of these data, which were used under license for the current study, and so are not publicly available. Data are however available from the authors upon reasonable request and with permission of the IRB.

## Abbreviations

AHRR: Aryl hydrocarbon repressor receptor
CEMA: 2-cyanoethylmercapturic acid
CO: Carbon monoxide
ddPCR: Droplet digital polymerase chain reaction
ELISA: Enzyme-linked immunosorbent assay
IRB: Institutional Review Board
NIH: National Institute of Health
PAH: Polycystic aromatic hydrocarbons
PHQ-9: Patient health questionnaire-9
THC: Tetrahydrocannabinol
W1: Wave 1; first annual in-person interview
W2: Wave 2; second annual in-person interview
W3: Wave 3; third annual in-person interview
