# Supplementary material for: Refinement of cg05575921 demethylation response in nascent smoking
Source: Clin Epigenetics. 2020 Jun 24;12:92. doi: 10.1186/s13148-020-00882-w (PMC7315557; doi:10.1186/s13148-020-00882-w)
Supplement: Supplementary file 1 — Additional file 1. Abbreviated child version of the Semi-structured Assessment for the Genetics of Alcoholism. [file 13148_2020_882_MOESM1_ESM.pdf]

Cigarette Smoking in the Home

A1. **Do you have any brother(s)/ sister(s) living at home who smoke?**

NO ....1 (If no skip to A2)

YES..2

Answer the following questions for the brother or sister who lives at home who smokes the most:

A1.1 When he or she smokes, how many cigarettes does he or she smoke a day? ______

A1.2 How often does he or she smoke cigarettes?

every day? 1

almost every day?..2

only 2-3 days a week?....... 3

1 or fewer days a week?.......................................4

A2. Does your mother smoke cigarettes?

NO ....1 (If no skip to A3)

YES..2

A2.1 **When she smokes, how many cigarettes does she smoke a day?** ______

A2.2 **How often does she smoke cigarettes?**

every day? 1

almost every day?..2

only 2-3 days a week?....... 3

1 or fewer days a week?.......................................4

2.3 **Does she smoke cigarettes in the house?**

NO 1

YES..5

A3. **Does your father smoke cigarettes?**

NO ....1 (If no skip to A4)

YES..2

A3.1 **When he smokes, how many cigarettes does he smoke a day?** ______

A3.2 **How often does he smoke cigarettes?**

every day? 1

almost every day?...2

only 2-3 days a week?....... ………………………….......3

1 or fewer days a week?.......................................4

A3.3 **Does he smoke cigarettes in the house?**

NO 1

YES..5

A4. **What is the total number of people in your home who smoke cigarettes?** ______

A4.1 **Are there places other than your home where you are regularly around adult cigarette smokers?**

NO ....1 (If no skip to 5)

YES...2

A5. W**hat is the approximate number of hours per week that you are exposed to smoke in these places?** ______

Smoking Willingness and Prototype Questions

Suppose you were with a group of kids and there were some cigarettes that you could have if you wanted. How willing would you be to do the following things?

F1. **How willing would you be to take a puff?**

1) not at all willing …………………………………..1

2) kind of willing ………………………………………2

3) very willing ……………………………………………3

F2. **How willing would you be to take one and smoke it?**

1) not at all willing ……………………………………1

2) kind of willing …………………………………………2

3) very willing ………………………………………………3

F3**. How willing would you be to take some cigarettes with you for later?**

1) not at all willing ……………………………………..1

2) kind of willing ……………………………………………2

3) very willing…………………………………………………2

Now I want to ask you some questions about your images of other people. Images are pictures we have in our mind about people and groups. For example, we all have ideas about what the type of kid your age who plays basketball is

like. We might say that the typical basketball player is tall and skinny. We are not saying that all of these people are alike, only that some of them are similar in some ways. In these questions, you will be asked to think about different images you have."

"A number of young people your age smoke. I want to know what you think about them. Take a moment and think about the type of kid your age who smokes. I am not thinking about anyone in particular, just your image of kids who smoke. "

Type of kids your age who smoke cigarettes...How popular are they?

(1) not at all

(2) a little bit

(3) kind of

(4) very

Type of kids your age who smoke cigarettes...How smart are they?

(1) not at all

(2) a little bit

(3) kind of

(4) very

Type of kids your age who smoke cigarettes...How cool are they?

(1) not at all

(2) a little bit

(3) kind of

(4) very

Type of kids your age who smoke cigarettes...How attractive (good-looking) are they?

(1) not at all

(2) a little bit

(3) kind of

(4) very

Type of kids your age who smoke cigarettes...How dull or boring are they?

(1) not at all

(2) a little bit

(3) kind of

(4) very

Alcohol Willingness and Prototypes Questions

"Suppose you were with a group of kids and there was some alcohol there that you could have if you wanted. How willing would you be to do the following things?"

How willing would you be to have a few sips?

(1) not at all willing

(2) kind of willing

(3) very willing

How willing would you be to drink one drink?

(1) not at all willing

(2) kind of willing

(3) very willing

How willing would you be to have more than one drink?

(1) not at all willing

(2) kind of willing

(3) very willing

"A number of young people your age drink alcohol. I want to know what you think about them. Take a moment and think about the type of kid your age who drinks. I am not thinking about anyone in particular, just your image of kids who drink".

Type of people your age who drink alcohol...How popular are they?

(1) not at all

(2) a little bit

(3) kind of

(4) very

Type of kids your age who drink alcohol...How smart are they?

(1) not at all

(2) a little bit

(3) kind of

(4) very

Type of people your age who drink alcohol...How cool are they?

(1) not at all

(2) a little bit

(3) kind of

(4) very

Type of kids your age who drink alcohol...How attractive (good-looking) are they?

(1) not at all

(2) a little bit

(3) kind of

(4) very

(Type of people your age who drink alcohol...How dull or boring are they?

(1) not at all

(2) a little bit

(3) kind of

(4) very

Marijuana Willingness and Prototype Questions

Suppose you were with a group of kids and there were some marijuana that you could have if you wanted. How willing would you be to do the following things?

How willing would you be to take a puff?

(1) not at all willing

(2) kind of willing

(3) very willing

How willing would you be to take a joint and smoke it?

(1) not at all willing

(2) kind of willing

(3) very willing

How willing would you be to smoke more than one joint?

(1) not at all willing

(2) kind of willing

(3) very willing

"Now I want to ask you some questions about your images of kids who smoke marijuana. Suppose you were with a group of kids and there was some marijuana there that you could have if you wanted. How willing would you be to do the following things?"

Type of people your age who smoke marijuana...How popular are they?

(1) not at all

(2) a little bit

(3) kind of

(4) very

Type of kids your age who smoke marijuana...How smart are they?

(1) not at all

(2) a little bit

(3) kind of

(4) very

Type of kids your age who smoke marijuana...How cool are they?

(1) not at all

(2) a little bit

(3) kind of

(4) very

Type of people your age who smoke marijuana...How attractive (good-looking) are they?

(1) not at all

(2) a little bit

(3) kind of

(4) very

Type of kids your age who smoke marijuana...How dull or boring are they?

(1) not at all

(2) a little bit

(3) kind of

(4) very

Risky Encounters

Have you experienced any inappropriate sexual contacts with someone you didn't want? By sexual contact I mean a person touching your sexual parts, you touching their sexual parts, or sexual intercourse.

NO…………………........... 1

YES……………….…………….5

Did this happen more than once?

NO 1

YES, MORE THAN ONCE 5

Internet Gaming

How much time each week do you spend on internet gaming sites?

Less than 1 hour? 1

1 to 3 hours?..2

4-8 hours?....... ……………………….........3

More than 8 hours?.......................................4

Skip Boxed Questions during the Intake Interview

Have you smoked **<u>cigarettes</u>** in the past 6 months? **If no, skip to next box, if yes continue.**		
How many have you smoked in the past	Day?	Number _________
(*<u>Please prompt with less than one, one, two etc)</u>*	Week?	Number ________
On average, how many cigarettes have you smoked each week		
In the past ? *(please prompt with less than one etc*)	Month?	Number ________
	6 months?	Number ________
	Year?	Number ________

Have you smoked **<u>cigars/cigarillos</u>** have you smoked in the past 6 months? (If no, skip to next box)		
How many have you smoked in the past	Day?	Number _________
	Week?	Number ________
On average, how many cigars/cigarillos have you smoked each week		
in the past (*<u>Please prompt with less than one etc)</u>*	Month?	Number ________
	6 months?	Number ________
	Year?	Number ________

Have you used **E-Cigarettes** in the past 6 months? (If no, skip to next box, if yes continue.)

How many times have you used E-Cigs in the past	Day?	Number _________
	Week?	Number ________
On average, how many times have you used E-Cigs each week	Month?	Number ________
in the past ? *(please prompt with less than one etc*)	6 months?	Number ________
	Year?	Number ________

**Have you used Chew in the past 6 months?** **(** If no, skip to next box, if yes continue.)		
How many times have you used chew in the past	Day?	Number _________
(*Please prompt with less than one, one, two etc)*	Week?	Number ________
On average, how many times have you used chew each week	Month?	Number ________
in the past ? *(please prompt with less than one etc*)	6 months?	Number ________
	Year?	Number ________

**Have you used Snuff in the past 6 months?** (If no, skip to below)		
How many times have you used snuff in the past	Day?	Number _________
(*Please prompt with less than one, one, two etc)*	Week?	Number ________
On average, how many times have you used snuff each week	Month?	Number ________
in the past ? *(please prompt with less than one etc*)	6 months?	Number ________
	Year?	Number ________

**Have you used Hookah or Waterpipes in the past 6 months?** (If no, skip to below)		
How many times have you used **Hookah or Waterpipes** in the past	Day?	
Number _________		
(*Please prompt with less than one, one, two etc)*	Week?	Number ________
On average, how many times have you used Hookah each week	Month?	Number ________
in the past ? *(please prompt with less than one etc*)	6 months?	Number ________
	Year?	Number ________

Risk for Gambling

In the past 12 months, would you say you have been preoccupied with gambling? Yes No

In the past 12 months, have you needed to gamble with larger amounts of money to get the same feeling of excitement? Yes No

In the past 12 months, have you often gambled longer, with more money or more frequently than you intended to? Yes No

In the past 12 months, have you made attempts to either cut down, control or stop gambling? Yes No

In the past 12 months, have you borrowed money or sold anything to get money to gamble? Yes No

C-SSAGA-A-IV
Jan 16, 2015

ENTER RESPONDENT'S I.D.:	__________ _____ _ _ ______
ENTER DATE OF INTERVIEW:	_______/________/____________ MONTH DAY YEAR
What kind of interview is this?	CHILD .... 1<br>PARENT…..……………………….. 2
Interviewers Initials	WLM EP NH

DM1 **RECORD SEX AS OBSERVED.**

MALE 1
FEMALE 2

DM2 How tall are you?

___ ______ FT IN

DM3 How much do you weigh?
A & B OMITTED

___ _______LBS

DM4 OMITTED

DM5 What is your birth date?

_______/_______/_____ DAY MO YEAR

CONFIRM AGE: So, you are_____years old?
CORRECT DOB IF R DOES NOT CONFIRM AGE

DM5 A. In what state were you born?
IF NOT BORN IN US, CODE NA

STATE: ______________________

B In what state do you currently live?
CODE STATE OF PHYSICAL (NOT LEGAL) RESIDENCE

STATE: ______________________

C What is your zip code?

ZIP CODE:

DM6 & DM7 OMITTED

DM8 Are you of Hispanic or Latino background?

NO 1
YES 5

HAND R CARD DM1.

B This card has the names of some racial groups. To which group do you belong?

CODE: _______

IF OTHER, SPECIFY:__________________

HAND R CARD DM2.

On this card is a list of countries that people may have come from.

What country did your grandparents come from? Let's start with your mother's mother. What country did your mother's mother come from?

K1. MOTHER'S MOTHER __ __
K2. MOTHER'S FATHER __ __
K3. FATHER'S MOTHER __ __
K4. FATHER'S FATHER __ __

C. What is your religion?
IF NONE, CODE 60 AND SKIP TO DM 8D.

SPECIFY: ________________________ __

CODE: _______

1. Does your religion have rules against us ing any alcohol?

NO
1

YES 5

D. In the past twelve months, how many times did you go to religious services?

___ _______TIMES

IF R IS LESS THAN 15 YEARS OLD, SKIP TO DM14K

DM9K Are you presently married or living as married, or have you never been married?

MARRIED…………………………..1
NEVER MARRIED………………..5
LIVING AS MARRIED…………..…6

DM10 OMITTED

DM11 OMITTED

DM12 OMITTED

DM13 OMITTED

DM14K **IF FEMALE:** Have you ever been pregnant?
IF MALE: Have you ever had any children?

NO.…(SKIP TODM15E)………1
YES...(IF MALE, SKIP TO DM14C)..5

DM14 How many times have you been pregnant? _______TIMES

A. Are you currently pregnant?

NO 1
YES...5

B. How many stillbirths and miscarriages have you had? ___ ___NUMBER

C. How many children have you had? ______ CHILDREN
RECORD SEX AND DOB.

	DATE OF BIRTH				DATE OF BIRTH		
SEX	MO	YEAR		SEX	MO	YEAR	
M F	/		*t*	M F	/		*t*
M F	/	_	*t*	M F	/		*t*
M F	/	_	*t*	M F	/		*t*
M F	_/	_	*t*	M F	_/		*t*

DM15E Are you currently in school?
IF SUMMER VACATION, CODE YES.

NO……………………………..…...1
YES……………………………..5

DM15 What grade are you in/What is the highest grade in school you finished?
CODE ACTUAL GRADE.
IF SUMMER, COUNT LAST GRADE COMPLETED.
IF CURRENTLY IN SCHOOL, SKIP TO DM16K1.

_______GRADE

DM15KB Why aren't you in school?

DROPPED OUT……………………1
EXPELLED……………………...2
ILLNESS……………………...…..3
GRADUATED .(SKIP TO DM16K1).4
OTHER……………………….…5

DM15KB1 How old were you when you (left/dropped out/were expelled from) school?

_______AGE

DM15B Did you get a GED?

NO……………………………1
YES…..(SKIP TO DM16K1)…….5

DM15B1 Are you working on a GED?

NO…………………………..……1
YES……………………………..5

DM16K1 Now I want to ask you about work for pay. In the last 12 months, have you had a job, like doing yard work, babysitting, or working in a store?

NO……..(SKIP TO MH1)……..1
YES……………………………..5

K2 In the last 12 months, how many weeks did you work at all?
COUNT SELF-EMPLOYMENT OR SALARIED.
IF LESS THAN 1 WEEK, CODE 1.

_______WEEKS

DM17 Are you working now?

NO..1
YES 5

IF R IS NOT CURRENTLY IN SCHOOL (DM15E=1), SKIP TO MH1.

K1. How many hours a week (do/did) you work during the school year?

___ ___HOURS

K2. How many hours a week (do/did) you work during the summer?

___ ___HOURS

DM18 OMITTED

MH1 Now I have some questions about your physical health and medical history. First, at the present time, would you say your health is excellent, very good, good, fair, or poor?	EXCELLENT....1<br>VERY GOOD....2<br>GOOD....3<br>FAIR....4<br>POOR....5
MH2 Has your health always been (ANSWER IN MH1), or has it been better or worse?	NO, WORSE....1<br>NO, BETTER....2<br>YES, SAME....5<br>BOTH BETTER & WORSE.....6

MH3 Has a doctor or other health professional ever told you that you have (had):

		NO	YES	YEAR FIRST DIAGNOSED
1.	OMITTED			
2.	Very bad headaches?	1	5	__ __ __ __
3.	A brain injury or concussion?	1	5	__ __ __ __
4.	Been knocked out or unconscious for longer than 5 min?	1	5	__ __ __ __
5.	Epilepsy or have had a seizure?	1	5	__ __ __ __
6.	A brain infection?	1	5	__ __ __ __
7.	OMITTED			
8.	Heart disease?....	1	5	__ __ __ __
9.	OMITTED			
10.	OMITTED			
11.	Asthma?....	1	5	__ __ __ __
12.	Diabetes?	1	5	__ __ __ __
13.	Cancer? **SPECIFY:** ________________________	1	5	__ __ __ __
14.	OMITTED			
15.	OMITTED			
16.	Any other serious illness(es)?____________________	1	5	__ __ __ __
17.	Another medical condition?_____________________	1	5	

MH4K	Have you ever stayed in the hospital overnight or longer?	NO……(SKIP TO MH4C)…….1<br>YES……………………………..5
A.	How many times have you been in a hospital overnight (including surgery and pregnancy), <u>not including</u> psychiatric or substance abuse treatment?	_______TIMES
B.	OMITTED	
KC.	Have you ever gone to the emergency room?<br>**COUNT URGENT CARE CLINIC.**	NO……(SKIP TO MH5)………1<br>YES……………………………..5
	C. How many times in your life have you had to go to the emergency room?	_______TIMES

MH5 In the last 6 months, how many visits have you made to a doctor, clinic, or emergency room for your physical health? **DO NOT COUNT CHIROPRACTORS OR ROUTINE PHYSICALS.**

___ ___ VISITS

MH6A Have you ever had to take any medicine that a doctor gave you for two weeks or longer (besides aspirin, Tylenol, or cough syrup, etc)?

NO……(SKIP TO MH8)………1
YES…………………………..5

What medicine(s) did you take?

____ CODE: ___ ___ ___

____ CODE: ___ ___ ___

____ CODE: ___ ___ ___

____ CODE: ___ ___ ___

____ CODE: _ ___

MH6B OMITTED

MH7 OMITTED

MH8 Has there ever been a time when you were having troubles or problems and went to talk to someone about them? For example, a school counselor, someone at your church/temple, a doctor, or someone else outside your family?

NO……. (SKIP TO TB1C) 1
YES...5

A. Did you speak to a ...?

	NO	YES
1. Psychiatrist	1	5
2. Psychologist	1	5
3. Social worker..	1	5
4. School counselor	1	5
5. Other medical doctor...	1	5
6. Nurse	1	5
7 Minister, priest, rabbi or imam	1	5
8. Another professional……………………………	1	5

IF YES, SPECIFY:________________________

MH9 OMITTED

MH10 OMITTED

Subject Screener for Tobacco, Alcohol, Cannabis, Depression and Suicidality Sections:

Now I'm going to ask you some questions about using tobacco.

(3) TB1C Have you ever:

1. Smoked a cigarette, even a puff?	NO……….(SKIP TO 2).............. 1<br>YES...5
ONS/REC. How old were you the (first/last) time you smoked a cigarette?	AGE ONS:____/___<br>ONS: 1 5<br><br>AGE REC:____/___<br>REC: 1 5
2. Smoked a cigar, even a puff?	NO……….(SKIP TO 3).............. 1<br>YES...5
ONS/REC. How old were you the (first/last) time you smoked a cigar?	AGE ONS:____/___<br>ONS: 1 5<br><br>AGE REC:____/___<br>REC: 1 5
3. Smoked a tobacco pipe, even a puff?	NO…………(SKIP TO 4)............ 1<br>YES...5
ONS/REC. How old were you the (first/last) time you smoked a tobacco pipe?	AGE ONS:____/___<br>ONS: 1 5<br><br>AGE REC:____/___<br>REC: 1 5
4. Used chewing tobacco?	NO……(SKIP TO BOX TB1)..... 1<br>YES...5
ONS/REC. How old were you the (first/last) time you used chewing tobacco?	AGE ONS:____/___<br>ONS: 1 5<br><br>AGE REC:____/___<br>REC: 1 5

If yes to any, complete Tobacco Section

---

(1) AL1 Now I would like to ask you some questions about your use of alcoholic drinks, like beer, wine, wine coolers, champagne, or hard liquor like vodka, gin, or whiskey. When I use the term „drink,‟ I mean a glass of wine, a can or bottle of beer, or a shot of liquor alone or in a mixed drink. Have you ever had a drink of alcohol?

NO... 1
YES…(SKIP TO AL1K)......... 5

A. So, you have never had even one full drink of alcohol?

NEVER… (SKIP TO MJ1) ... 1
YES, HAD A DRINK 5

If Yes to either, complete Alcohol Section

Marijuana

MJ1	Have you ever used marijuana or hashish?	NO……..(SKIP TO DR1)………1<br>YES………………..………….5

If yes, complete Marijuana section

Now I'm going to ask you some questions about your mood.

DP1	Have you ever had a period of time when everyday or nearly every day, you felt sad, depressed, or unhappy most of the time for two weeks?<br>(PROBE: What I mean is, these feelings didn't happen just for one or two days, but you felt like this most of the day, nearly every day for at least two weeks.)	NO ....1<br>YES...5
DP2	Have you ever had a period of time when nothing seemed fun anymore? For example, you lost interest in things that you usually liked to do? These might be things like not wanting to hang out with your friends, not wanting to take part in your hobbies or after school activities.	NO………(SKIP TO A) ....1<br>YES...5
	K1. Did these feelings of things not being fun anymore last most of the day, nearly every day for two weeks or more?	NO ....1<br>YES...5
	A. Have you ever had a period of time when you felt irritable, crabby, or in a bad mood most of the time for two weeks or more?	NO…(SKIP TO BOX DP2) ....1<br>YES...5
	K2. Did these feelings last most of the day, nearly every day for two weeks or more?	NO ....1<br>YES...5

BOX DP2 IF DP1, DP2K1, and DP2K2 ALL CODED 1, Skip Depression Section.

Suicidality Screener

IF DP27B14=5, SKIP TO SU1A

Now I would like to ask you some (more) questions about times when you might have been feeling really down and felt like nothing would ever go right with you.

SU1. Have you ever thought about killing yourself?	NO..1<br>YES 5
SU2. Have you ever tried to kill yourself?	NO..1<br>YES 5

If Yes to either, complete Suicidality Section

Tobacco Section

BOX TB1 IF TB1.C1=1, SKIP TO NEXT PAGE

D. Have you ever smoked a full cigarette?

NO …..……(SKIP TO next page) .1
YES 5

ONS. How old were you the first time you smoked a full cigarette?

AGE ONS: ___/___
ONS: 1 5

E. From whom did you get your first cigarette?

SPECIFY OTHER: ________________________________

SELF……..…………………….…1
FRIEND/PEER…………………..2
MINOR FAMILY..……………….3
ADULT FAMILY..……………….4
PARENT…………………………5
OTHER ADULT…..……………..6

F. From whom do you usually get your cigarettes?

SELF……..…………………….…1
FRIEND/PEER…………………..2
MINOR FAMILY..……………….3
ADULT FAMILY..……………….4
PARENT…………………………5
OTHER ADULT..………………..6

G. Did you enjoy your first experience with smoking …

A LOT?…………………………..1
SOME?…………………………...2
A LITTLE?……………………….3
NOT AT ALL?…………………..4

H. The first few times you smoked, did you . . .	NO	YES
1. cough?…………………………………………………	1	5
2. feel dizzy or light-headed?…………………………….	1	5
3. get a headache?……………………………………….…..	1	5
4. feel your heart racing?……………………………………	1	5
5. feel nauseated, like vomiting?……………………………	1	5
6. experience anything else, either good or bad? …………..	1	5
**IF YES:**		
6.1 Did you experience positive effects?………………….	1	5
6.2 Did you experience other negative effects?……………	1	5

---

(4) TB2 OMITTED

---

(5) TB3 Over your lifetime, have you smoked a total of 100 cigarettes (smoked 5 or more packs)?

NO………......................................1
YES ………(SKIP TO A)………5

1. Over your lifetime, have you smoked a total of 20 cigarettes (smoked 1 pack or more)?

NO………......................................1
YES …………………..………5

A. What is the largest number of cigarettes you've ever smoked in a single day?

NUMBER: ___ ___ ___

How many **<u>cigarettes</u>** have you smoked in the past	Day?	Number _________
(*<u>Please prompt with less than one, one, two etc)</u>*	Week?	Number ________
On average, how many cigarettes have you smoked each week	Month?	Number ________
In the past ? *(please prompt with less than one etc*)	6 months?	Number ________
	Year?	Number ________

How many **<u>cigars/cigarellos</u>** have you smoked in the past	Day?	Number _________
<u>Please prompt with less than one, one, two etc</u>	Week?	Number ________
On average, how many cigars/cigarellos have you smoked each week		
in the past (*<u>Please prompt with less than one etc)</u>*	Month?	Number ________
	6 months?	Number ________
	Year?	Number ________

**Do you use E-Cigarettes?** (If no, skip to Chew question)		
How many times have you used E-Cigs in the past	Day?	Number _________
(*<u>Please prompt with less than one, one, two etc)</u>*	Week?	Number ________
On average, how many times have you used E-Cigs each week	Month?	Number ________
in the past ? *(please prompt with less than one etc*)	6 months?	Number ________
	Year?	Number ________

**Do you use Chew?** (If no, skip to Snuff question)		
How many times have you used chew in the past	Day?	Number _________
(*<u>Please prompt with less than one, one, two etc)</u>*	Week?	Number ________
On average, how many times have you used chew each week	Month?	Number ________
in the past ? *(please prompt with less than one etc*)	6 months?	Number ________
	Year?	Number ________

**Do you use Snuff?** (If no, skip to below)		
How many times have you used snuff in the past	Day?	Number _________
(*<u>Please prompt with less than one, one, two etc)</u>*	Week?	Number ________
On average, how many times have you used snuff each week	Month?	Number ________
in the past ? *(please prompt with less than one etc*)	6 months?	Number ________
	Year?	Number ________

IF TB3.1=1 (HAS NOT SMOKED 100+ CIGARETTES LIFETIME), SKIP TO AL1.

(1 & 6) TB4A. If you are/were smoking regularly, how many days a week did you usually smoke cigarettes? **IF NOT AS OFTEN AS ONCE A WEEK, CODE 0.** ___ DAYS

IF DK, ASK:

1. Did you usually smoke at least 2 days a week?

NO 1
YES...5

ND45(=20) B. How many cigarettes did you usually smoke in a day? ___ ___ ___ CIGS*

IF DK, ASK:

1. Did you usually smoke at least 20 cigarettes in a day?

NO……………………………..1
YES .... …5*

,

C. For about how long did you smoke this many cigarettes at that rate? __ __ __ NUM

CODE UNITS:
DAYS..1
WEEKS..2
MONTHS................................... 3
YEARS..4

ONS/REC. How old were you the (first/last) time you smoked cigarettes at that rate?

AGE ONS: ___/___
ONS: 1 5

AGE REC: ___/___
REC: 1 5

Think about the period lasting a month or more when you were smoking the most.

(7) TB5x. During this period when you were smoking the most, about how many cigarettes did you usually have in a day? ___ ___ CIGS

IF DK: Was it usually (READ OPTIONS) . . .

10 OR LESS…………………..1
11-20…………………………2
21-30…………………………3
31 OR MORE………………..4

TB5 During this period when you were smoking the most, about how many minutes after you woke up did you smoke your first cigarette? ___ ___ ___ MINUTES

IF DK: A. Was it usually (READ OPTIONS)? . . .

WITHIN 5 MINUTES? 1
WITHIN 6-30 MINUTES? 2
WITHIN 31-60 MINUTES? 3
MORE THAN ONE HOUR?. 4

---

(8) TB6 When you were smoking the most, did you usually smoke more frequently during the first hours after waking than during the rest of the day?

NO 1
YES 5

(9)	TB7	When you were smoking the most, did you usually find it difficult to keep from smoking in places where it was not allowed; for example, at school, in church, at the library, in movie theaters, or when someone asked you not to?	NO ....1<br>YES 5
	TB8	When you were smoking the most, which cigarette would you have hated most to give up: the first one in the morning, after eating, while watching television, or some other one?	ANY OTHER .... 1<br>FIRST ONE IN MORNING 5
(10)	TB9	When you were smoking the most, were there times you smoked even when you were so sick that you had to be in bed most of the day?	NO ....1<br>YES 5

Now I'd like you to think about your cigarette smoking throughout your life as I ask you more questions about experiences people sometimes have when they smoke cigarettes. (Since you don't smoke now, I'd like to ask you about the times when you used to smoke cigarettes.)

(11)	TB10	Did you ever chain smoke; that is, where you smoked several cigarettes, one right after another?	NO……(SKIP TO TB11)....1<br>YES……5
		A. For how many hours in a row did you smoke like that?<br>**CODE LESS THAN 1 HOUR = 0.**	___ ___ HOURS
		1. **IF DK**: Did you ever smoke like that for 3 hours or more?	NO.... 1<br>YES……...5
		B. How many cigarettes did you smoke in a row?	___ ___ NUMBER

BOX TB10 IF LESS THAN 3 CIGARETTES, SKIP TO TB11.

		1. **IF DK**: Was it at least 3 in a row?	NO……(SKIP TO TB11)…….1<br>YES……...5
ND45		C. What is the longest period of time you have chain smoked every day or nearly every day?<br>**IF 7 OR MORE DAYS, MARK TALLY SHEET.**	___ ___ NUM<br>**CODE UNITS:**<br>DAYS……1<br>WEEKS…….2*<br>MONTHS…….3*<br>YEARS… ……..4*
(12)<br>ND46	TB11	Have you ever stopped doing things with any of your good friends because of your smoking?	NO ....1<br>YES……...5*
		A. Have you missed activities, club meetings, or sports practices because of your smoking?	NO ....1<br>YES……...5*
(13)<br>ND43	TB12	Have you often smoked a lot more than you meant to or for more days in a row than you meant to? For example, smoking half a pack or more when trying to smoke only 1 or 2 cigarettes?	NO ....1<br>YES……...5*
ND43		A. Have you often found that you've run out of cigarettes sooner than you meant to?	NO ....1<br>YES……...5*

(14) ASP3RC7 ASP4A5	TB13	Have you smoked in situations where it was dangerous to smoke; for example, smoking in bed, or around things like gasoline, paint thinners, or cleaning fluids?	NO……(SKIP TO TB14).......... 1<br>YES 5
	A.	Did this happen a total of 3 or more times?	NO…….(SKIP TO TB14)......... 1<br>YES 5
	B.	Have you smoked in a dangerous situation 3 or more times in any 12-month period?	NO .... 1<br>YES 5
(15) ND44	TB14	Have you often wanted to quit or cut down on smoking?	NO .... 1<br>YES 5*
	A.	Have you ever tried to quit smoking for at least 24 hours?	NO……(SKIP TO B1). .... 1<br>YES……………………………5
	B.	How many times did you try to quit?<br>**SKIP TO C**	___ ___ ___ TIMES
		1. Have you ever tried to cut down, by about half, the number of cigarettes you smoked?	NO….(SKIP TO TB15)............. 1<br>YES………………….………5
	C.	Were you always able to stop or cut down when you wanted to?	NO……...(SKIP TO D).............. 1<br>YES 5
		1. Were you able to stop or cut down for at least 1 month?	NO .... 1<br>YES…..(SKIP TO TB15)......... 5
ND44	D.	Have you found that you were unable to stop or cut down on smoking (for at least 1 month) at least 3 times?	NO .... 1<br>YES 5*
(16)	TB15	Since you began smoking regularly, what is the longest period of time you have gone without using any form of tobacco for any reason, like when you had an illness, or lost interest in tobacco, or intentionally quit? **IF NEVER, CODE 0 DAYS. IF LESS THAN ONE DAY, CODE 1 DAY**.	___ ___ NUM<br>**CODE UNITS:**<br>DAYS.................................. 1<br>WEEKS ....2<br>MONTHS .... 3<br>YEARS 4

BOX TB15 **IF TB15 = 0 DAYS, SKIP TO TB16. OTHERS CONTINUE.**

(23)	A.	Have you ever gone to a class or group for people trying to quit or cut down their use of tobacco?	NO .... 1<br>YES 5
	B.	Have you ever tried nicotine gum or a nicotine patch (to quit or cut down your use of tobacco)?	NO .... 1<br>YES 5
	C.	Have you ever tried nicotine-free cigarettes (to quit or cut down your use of tobacco)?	NO .... 1<br>YES 5
	D.	Have you tried any other form of treatment or medicine to quit or cut down your use of tobacco?	NO .... 1<br>YES……(SPECIFY) 5

SPECIFY:____

____

E. OMITTED

(17) TB16 I'm going to ask you about some problems that you might have had when you stopped smoking or smoked fewer cigarettes than usual. Think about the time when you had the most problems when you went without cigarettes or had fewer than usual.

I-SX

During that time:	NO	YES
1. Were you irritable, angry, or frustrated?	1	5
2. Were you nervous or anxious?	1	5
3. Were you restless?	1	5
4. Did you have trouble paying attention?	1	5
5. Did your heart slow down?	1	5
6. Did you feel down or depressed?	1	5
7. Did you have such a strong need for cigarettes that you couldn't think of anything else?	1	5
8. Did your appetite increase or did you gain weight?	1	5
9. Did you have trouble sleeping?	1	5

BOX TB16

A. OMITTED

B. OMITTED

C. Did the problems you had after quitting or cutting down on smoking often interfere with how you got along at school or other activities?
NO 1
YES 5

D. Did you start smoking again or use other kinds of nicotine (e.g., chewing tobacco) to make these problems go away, or to keep them from happening again?
NO 1
YES 5*

---

(18) TB17 Has smoking ever made you nervous or jittery or caused you any other emotional or mental problem?
NO…….(SKIP TO TB18)........ 1
YES 5

A. Did this keep you from doing the things you normally do?
NO……..(SKIP TO TB18)........ 1
YES 5

ND47

B. Did you keep on smoking after you knew it caused you problems like these?
NO 1
YES 5*

(19)	TB18	Has smoking caused you any health problem, such as a problem with your heart, lung trouble, a cough that wouldn't go away, or anything like that?<br><br>**SPECIFY:** ______________________________________	NO……..(SKIP TO TB19)…..1<br>YES………..(SPECIFY)……..5<br><br>CODE:___ ___ ___
ND47		A. Did you keep on smoking after you knew it caused you (this/these) health problem(s)?	NO……………………………1<br>YES…..……………………….5*
(20)<br>ND47	TB19	Have you kept smoking when you had another serious illness that you knew was made worse by smoking, for example: asthma or bronchitis?<br><br>**SPECIFY:** ______________________________________	NO .... 1<br>YES………(SPECIFY)……….5*<br><br>CODE: ___ ___ ___
(21)	TB20	A. After you had been smoking for a while, did you need to smoke more to keep getting the effects you wanted?	NO .... 1<br>YES………(SKIP TO C)……..5
		B. After you had been smoking for a while, did you come to need more cigarettes each day?	NO………..(SKIP TO D) .... 1<br>YES……………………………5
ND41A		C. Was this a big increase? So, if you used to smoke 10 cigarettes a day, did you increase to 15 a day, or go from 20 to 30?	NO .... 1<br>YES..(SKIP TO BOX TB21)….5*
ND41B		D. After you had been smoking for a while, did you find that cigarettes had less effect on you than before?	NO……………………………1<br>YES…..……………………….5*

Alcohol Section

AL1 continued.

ONS. How old were you the first time you had your very first whole drink? Age in Years ____

(7) AL2 I'd like to ask you about reactions that some people have when they drink any type of alcohol.

A. While drinking, has one or two drinks of alcohol ever made you . . . **CODE IN COL. A.**

DO NOT COUNT IF ONLY ONE TYPE OF ALCOHOL CAUSED THE REACTION.

	COL A		COL B	
	NO	YES	NO	YES
1. flush or blush--that is, your face and hands felt hot and your face turned red?..	1	5	1	5
a. **IF AL2A.1=5, ASK**: Did you flush or blush a few minutes after only one drink?	1	5		
2. break out into a rash?	1	5	1	5
3. feel very sleepy (when you weren't already tired)?.......	1	5	1	5
4. feel sick to your stomach?	1	5	1	5
5. have headaches, head pounding, or throbbing?	1	5	1	5
6. feel your heart beating hard inside your chest?............	1	5	1	5

FOR EACH 5 CODED IN COL. A, ASK B. OTHERS SKIP TO AL3.

B. Did (SX) ever keep you from drinking any alcohol? Yes No

-

AL 2C. How many drinks of alcohol have you had in the past	Day?	Number _______
	Week?	Number _______
AL 2D On average, how many drinks per week have you had the past	Month?	Number _______
	6 months?	Number ________
	Year?	Number ________

.

(2) AL3 Let"s talk about the last week. Did you drink anything with alcohol in it during the last seven days?

NO………(SKIP TO C) 1
YES 5

I"d like to know about the alcohol you have had each day in the last week. Today is _____________. Let's begin with yesterday.

How many drinks of (beer, wine, liquor) did you have on (DAY OF WEEK)?

REFER TO CARD AL1 FOR THE DEFINITION OF A STANDARD DRINK.
IF OTHER, RECORD SPECIFIC DRINK NAME.

	BEER	WINE	LIQUOR	OTHER	(SPECIFY DRINK)
M	______	______			__________________
Tu	______	______			__________________
W	______	______			__________________
Th	______	______			__________________
F	______	______			__________________
Sa	______	______			__________________
Su	______	______			__________________

B. OMITTED

BOX AL3 IF R DRANK THIS WEEK (AL3=5), SKIP TO D.

C. When was the last time you had a drink with alcohol?
IF > 1 YEAR AGO, SKIP TO ALF1K.

_____/ __________*t*
MO YEAR

IF DK DATE, ASK:
How old were you the last time you had a drink with alcohol?

AGE REC:____/____*t*
REC: 1 5

IF AGE REC=CURRENT AGE OR 1 YEAR LESS THAN CURRENT AGE AND REC=1, OR IF AGE REC IS UNKNOWN, ASK:

C1. Did you have anything to drink within the past 12 months?

NO…(SKIP TO AL4F1K).....1
YES….(SKIP TO AL4A)….. 5

IF R DID NOT DRINK IN PAST WEEK, SKIP TO AL4A. OTHERS CONTINUE.

D. Was your drinking last week typical of the way you have been drinking during the past 6 months?

NO 1
YES…. 5

(3) AL4 A. Think about your use of alcohol over the past 6 months. During this period, on how many weeks did you drink alcohol? ___ ___ WEEKS

IF EVERY WEEK, CODE 26. IF 0, SKIP TO AL4E1K.

IF AL3D=5, SKIP TO AL4C

B. I"d like to know about the alcoholic drinks that you would usually have each day when you drank any alcohol. Think about a week in the last 6 months that is an example of the way you usually drank. Let"s start with the weekend. On a usual Friday night when you drank, how many (beers, coolers, etc.) would you have?

REFER TO CARD AL1 FOR THE DEFINITION OF A STANDARD DRINK.
IF OTHER, RECORD SPECIFIC DRINK NAME.

	BEER	WINE	LIQUOR	OTHER	(SPECIFY DRINK)
M	______	______			__________________
Tu	______	______			__________________
W	______	______			__________________
Th	______	______			__________________
F	______	______			__________________
Sa	______	______			__________________
Su	______	______			__________________

C. I'd like you to think about the week in the last 6 months when you drank the most. How many days did you drink during that week? ___DAYS

IF R VOLUNTEERS THAT NO WEEK STANDS OUT AS THE HEAVIEST (I.E., TYPICAL= HEAVIEST), CODE 0 AND SKIP TO AL4E1K.

D. How many drinks did you have on a typical day during that week? _______DRINKS

E. OMITTED

Now I'd like you to think about the last 12 months. I‟m going to ask you some questions about this period

AL4 E1K. Did you ever have 5 or more drinks in 24 hours?

NO………(SKIP TO E2K)..... 1
YES………………………...5

HAND R CARD AL2

E1. **IF YES:** How often did this happen during the last 12 months?

EVERY DAY 1
5-6 DAYS A WEEK (NEARLY EVERYDAY) 2
ABOUT 4 DAYS A WEEK (200-259 DAYS)..........3
ABOUT 3 DAYS A WEEK (150-199 DAYS)..........4
ABOUT 2 DAYS A WEEK (100-149 DAYS)..........5
ABOUT 1 DAY A WEEK (50-99 DAYS) 6
ABOUT 3 DAYS A MONTH (36-49 DAYS)...........7
ABOUT 2 DAYS A MONTH (24-35 DAYS)...........8
ABOUT 1 DAY A MONTH (12-23 DAYS) 9
ABOUT 6-11 DAYS A YEAR 10
ABOUT 3-5 DAYS A YEAR..................................11
ABOUT 1 TO 2 DAYS A YEAR 12

E2K. During the last 12 months did you ever get drunk? By "drunk" I mean that you couldn‟t talk clearly and it was hard to keep your balance.

NO………(SKIP TO E3) 1
YES………………………...5

E2. **IF YES:** How often did you get drunk during the last 12 months?

EVERY DAY 1
5-6 DAYS A WEEK (NEARLY EVERYDAY) 2
ABOUT 4 DAYS A WEEK (200-259 DAYS)..........3
ABOUT 3 DAYS A WEEK (150-199 DAYS)..........4
ABOUT 2 DAYS A WEEK (100-149 DAYS)..........5
ABOUT 1 DAY A WEEK (50-99 DAYS) 6
ABOUT 3 DAYS A MONTH (36-49 DAYS)...........7
ABOUT 2 DAYS A MONTH (24-35 DAYS)...........8
ABOUT 1 DAY A MONTH (12-23 DAYS) 9
ABOUT 6-11 DAYS A YEAR 10
ABOUT 3-5 DAYS A YEAR..................................11
ABOUT 1 TO 2 DAYS A YEAR 12

E3. During the last 12 months, about how many days did you drink anything with alcohol in it?

EVERY DAY 1
5-6 DAYS A WEEK (NEARLY EVERYDAY) 2
ABOUT 4 DAYS A WEEK (200-259 DAYS)..........3
ABOUT 3 DAYS A WEEK (150-199 DAYS)..........4
ABOUT 2 DAYS A WEEK (100-149 DAYS)..........5
ABOUT 1 DAY A WEEK (50-99 DAYS) 6
ABOUT 3 DAYS A MONTH (36-49 DAYS)...........7
ABOUT 2 DAYS A MONTH (24-35 DAYS)...........8
ABOUT 1 DAY A MONTH (12-23 DAYS) 9
ABOUT 6-11 DAYS A YEAR 10
ABOUT 3-5 DAYS A YEAR..................................11
ABOUT 1 TO 2 DAYS A YEAR 12

AL4 Fx. We just talked about your drinking over the past 12 months. Now I'd like you think about the 12-month period when you drank the most. Would this be a different 12-month period than the one we just talked about?

NO……..(SKIP TO AL5)…..1
YES… 5

(Now I'd like you to think about the 12-month period in your life when you drank the most. I"m going to ask you some questions about this period.) **HAND R CARD AL2.**

AL4 F1K. During that 12-month period in your life when you drank the most, did you ever have 5 or more drinks in 24 hours?

NO…….(SKIP TO F2K) 1
YES………………………...5

F1. **IF YES:** How often did this happen during those 12 months?

EVERY DAY 1
5-6 DAYS A WEEK (NEARLY EVERYDAY)....... 2
ABOUT 4 DAYS A WEEK (200-259 DAYS) 3
ABOUT 3 DAYS A WEEK (150-199 DAYS) 4
ABOUT 2 DAYS A WEEK (100-149 DAYS) 5
ABOUT 1 DAY A WEEK (50-99 DAYS)................ 6
ABOUT 3 DAYS A MONTH (36-49 DAYS).......... 7
ABOUT 2 DAYS A MONTH (24-35 DAYS).......... 8
ABOUT 1 DAY A MONTH (12-23 DAYS) 9
ABOUT 6-11 DAYS A YEAR 10
ABOUT 3-5 DAYS A YEAR 11
ABOUT 1 TO 2 DAYS A YEAR 12

F2K. During that 12-month period in your life when you drank the most, did you ever get drunk?

NO………(SKIP TO F3)........ 1
YES………………………...5

F2. **IF YES:** How often did you get drunk during those 12 months?

EVERY DAY 1
5-6 DAYS A WEEK (NEARLY EVERYDAY)....... 2
ABOUT 4 DAYS A WEEK (200-259 DAYS) 3
ABOUT 3 DAYS A WEEK (150-199 DAYS) 4
ABOUT 2 DAYS A WEEK (100-149 DAYS) 5
ABOUT 1 DAY A WEEK (50-99 DAYS)................ 6
ABOUT 3 DAYS A MONTH (36-49 DAYS).......... 7
ABOUT 2 DAYS A MONTH (24-35 DAYS).......... 8
ABOUT 1 DAY A MONTH (12-23 DAYS)............ 9
ABOUT 6-11 DAYS A YEAR 10
ABOUT 3-5 DAYS A YEAR 11
ABOUT 1 TO 2 DAYS A YEAR 12

F3. During that 12-month period in your life when you drank the most, about how many days did you drink anything with alcohol in it?

EVERY DAY 1
5-6 DAYS A WEEK (NEARLY EVERYDAY)....... 2
ABOUT 4 DAYS A WEEK (200-259 DAYS) 3
ABOUT 3 DAYS A WEEK (150-199 DAYS) 4
ABOUT 2 DAYS A WEEK (100-149 DAYS) 5
ABOUT 1 DAY A WEEK (50-99 DAYS)................ 6
ABOUT 3 DAYS A MONTH (36-49 DAYS).......... 7
ABOUT 2 DAYS A MONTH (24-35 DAYS).......... 8
ABOUT 1 DAY A MONTH (12-23 DAYS)............ 9
ABOUT 6-11 DAYS A YEAR 10
ABOUT 3-5 DAYS A YEAR 11
ABOUT 1 TO 2 DAYS A YEAR 12

ONS/REC. How old were you when that period (began/ended)?

AGE ONS:___/___

AGE REC:___/___
REC: 1 5

(4) AL5 How old were you when you started to drink regularly; that is, drinking at least once a month for 6 months or longer? **IF NEVER, CODE 0.**

AGE: ___ ___ *t*

BOX AL5A IF AL4E2K=5 OR AL4F2K=5, SKIP TO AL5A.

1. Have you ever gotten drunk, where you couldn‟t talk clearly and it was hard to keep your balance?

NO..(SKIP TO BOX AL5B)..1
YES…………………………5

A. How old were you the very first time you got drunk, (that is, where you couldn‟t talk clearly and it was hard to keep your balance)?

AGE: _______

B. OMITTED

BOX AL5B IF TB3=5, CONTINUE. OTHERS SKIP TO AL6.

C. When you drink, do you almost always smoke cigarettes at the same time? **COUNT TOBACCO ONLY.**

NO 1
YES 5

(5) AL6 In your lifetime, what is the largest number of drinks you have ever had in a 24-hour period? I mean whole drinks, like a can of beer or a shot of hard liquor, not just sips.

IF DK, ASK AL6.1. OTHERS SKIP TO A.

___ ___ ___ DRINKS

1. Did you ever have 3 or more drinks in a 24-hour period?

NO…….(SKIP TO AL6A)…1
YES 5

A. In the past 6 months, what is the largest number of drinks you've drunk in a 24-hour period?

___ _______DRINKS

BOX AL7 IF NEVER HAD 3 OR MORE DRINKS IN A 24-HR PERIOD AND DRANK LESS THAN 10 DRINKS LIFETIME [(AL6<3 OR AL6_DK=1) AND (AL1.K<10 OR AL1K_DK<2)], SKIP TO MJ1.

OTHERS CONTINUE.

(6) AL8 Was there ever a time when you drank almost every day for a week or more? By "almost every day" I mean at least 4 days out of 7.

NO……(SKIP TO AL9) 1
YES 5

A. Think about those periods of time when you drank almost every day (again, at least 4 out of 7 days). What was the largest number of drinks that you would drink almost every day for at least 1 week? **ALMOST EVERY DAY = 4 OUT OF 7.**

_______DRINKS

B. So, almost every day during this period you drank at least (# FROM A) drinks?

NO……....(RE-ASK A)...........1
YES 5

ONS. How old were you when this period began?

AGE ONS: ___/___
ONS: 1 5

D. How long did this period last?

_______WEEKS

.

(20) Now I‟m going to ask you about how things might have changed for you since you started drinking.

AL9 Have you ever found that when you drank the same amount you used to, it didn‟t affect you as much?

NO………(SKIP TO E)...........1
YES...5

A1. When you first started drinking regularly, how many drinks did it take you to get an effect?

___ _______DRINKS

A2. Later on, how many drinks did you need to get this effect? **CODE THE TYPICAL UPPER BOUND OF TOLERANCE. DO NOT COUNT AN ISOLATED EXPERIENCE.**

___ _______DRINKS

B. How old were you the (first/last) time you needed (# IN A2) drinks to get an effect?

AGE ONS:____/____
ONS: 1 5

AGE REC:____/____
REC: 1 5

C. WAS THE INCREASE IN A2 TO 4 DRINKS OR MORE?

NO ……..(SKIP TO E)……....1
YES ………………………...5

AD3RA7
AD41A
ADICD4

D. WAS INCREASE 50% OR MORE? CHECK CARD AL3.

NO ………………………….1
YES ……(SKIP TO AL10)….5*

E. Have you ever needed to drink a lot more alcohol than you used to in order to get drunk? For example, did you once need 2 beers to get drunk but later needed to drink 4 beers to feel the same way?

NO……(SKIP TO AL10) 1
YES....................................... 5

F1. When you first started drinking, how many drinks did it take you to get drunk?

___ _______DRINKS

F2. Later on, how many drinks did it take to get drunk? **CODE THE TYPICAL UPPER BOUND OF TOLERANCE. DO NOT COUNT AN ISOLATED EXPERIENCE.**

___ _______DRINKS

G. How old were you the (first/last) time you needed (# IN F2) drinks to get drunk?

AGE ONS:____/____
ONS: 1 5

AGE REC:____/____
REC: 1 5

H. WAS THE INCREASE IN F2 TO 4 DRINKS OR MORE?

NO …...(SKIP TO AL10)……1
YES ……………………….. 5

AD3RA7
AD41B
ADICD4

I. WAS INCREASE 50% OR MORE? CHECK CARD AL3.

NO ………………………… 1
YES ……………………….. 5*

(9)<br>AD3RA2/B<br>AD44<br>ADICD2	AL10 Have you 3 or more times wanted to stop or cut down on how much you drank?<br>**DO NOT COUNT DIETING OR PREGNANCY.**	NO……….(SKIP TO B) .... 1<br>YES…………………………5*
	A. How old were you the (first/last) time?	AGE ONS:___/___<br>ONS: 1 5<br>AGE REC:___/___<br>REC: 1 5
	B. Have you ever tried to stop or cut down on drinking?<br>**COUNT ANY REASON.**	NO…….(SKIP TO AL11) .... 1<br>YES 5
AD3RA2<br>ALCFGNB1	C. Were you always able to stop or cut down on drinking when you tried to?	NO, UNABLE............... …....1*<br>YES…(SKIP TO AL11) ..…….5
AD44<br>ADICD2	D. How many times were you unable to stop or cut down?<br>**IF 3 OR MORE, IF DK, ASK D1. OTHERS SKIP TO E.**	_______TIMES*
AD44<br>ADICD2	1. Was it 3 or more times?	NO .... 1<br>YES…………………………5*
	E. How old were you the (first/last) time?	AGE ONS:___/___<br>ONS: 1 5<br>AGE REC:___/___<br>REC: 1 5
(21)<br>ALCFGNB2	AL11 Some people try to control their drinking by making rules, like not drinking before 5 o'clock or never drinking alone. Have you ever made any rules to control your drinking?	NO ……(SKIP TO AL12) .... 1<br>YES 5
	ONS. How old were you the first time?	AGE ONS:___/___<br>ONS: 1 5
(13)<br>ALCFGNB1	AL12 Have you ever drunk much more than you really meant to? For example, when you decided to drink 2 drinks and ended up drinking 4 or more?	NO .... 1<br>YES…(SKIP TO ONS/REC)... 5
	A. Have you ever continued drinking for more days in a row than you meant to?	NO…….(SKIP TO AL13) .... 1<br>YES 5
	ONS/REC. How old were you the (first/last) time?	AGE ONS:___/___<br>ONS: 1 5<br>AGE REC:___/___<br>REC: 1 5
AD3RA1/B<br>AD43<br>ADICD2	C. Did this happen 3 or more times?	NO…………………………...1<br>YES…………………….….5*

(14)	AL13 Have you ever started drinking and become drunk when you didn't want to?	NO…….(SKIP TO AL14) .... 1<br>YES 5
	ONS/REC. How old were you the (first/last) time?	AGE ONS:____/____<br>ONS: 1 5<br>AGE REC:____/____<br>REC: 1 5
AD3RA1/B<br>AD43<br>ADICD2	B. Did this happen 3 or more times?	NO…………………………...1<br>YES………………………….5*
(22)	AL14 Have you ever stopped doing things with any of your good friends because of your drinking? Or have you missed regular activities, like club meetings or sports practices because you were drinking, drunk, or hung over?	NO……..(SKIP TO AL15) ...... 1<br>YES 5
	ONS/REC. How old were you the (first/last) time?	AGE ONS:____/____<br>ONS: 1 5<br>AGE REC:____/____<br>REC: 1 5
AD3RA5/B<br>AD46<br>ADICD5	B. Did this happen 3 or more times or for a month or more?	NO .... 1<br>YES…………………………5*
(15)	AL15 Have you ever spent <u>a lot</u> of your time getting alcohol, drinking alcohol, or trying to feel better after drinking alcohol?	NO…….(SKIP TO AL16) .... 1<br>YES 5
AD3RA3<br>AD45<br>ADICD5	A. Did this period last for a month or more or did you have 3 or more periods like that?	NO..……(SKIP TO AL16)........ 1<br>YES…………………………5*
	ONS/REC. How old were you the (first/last) time?	AGE ONS:____/____<br>ONS: 1 5<br>AGE REC:____/____<br>REC: 1 5

(12)	AL16 Have you ever been drunk for 2 days or more without sobering up, except for sleeping?	NO…….(SKIP TO AL17K)..... 1<br>YES 5
	A. Did this keep you from doing schoolwork, homework, chores or other things you were supposed to do?	NO…..(SKIP TO AL17K) .... 1<br>YES 5
AD3RA4/B<br>ADICD5<br>ALCFGNA4	B. How many times has this happened?<br>**IF 3 OR MORE, SKIP TO ONS/REC. IF DK, ASK B1.**<br>**OTHERS SKIP TO ONS/REC.**	___ _______TIMES *
AD3RA4/B<br>ADICD5<br>ALCFGNA4	1. Did this happen 3 or more times?	NO .... 1<br>YES…………………………5*
	ONS/REC. How old were you the (first/last) time this happened?	AGE ONS:____/____<br>ONS: 1 5<br>AGE REC:____/____<br>REC: 1 5
	**IF FEWER THAN 3 BINGES, SKIP TO AL17K**	
AA4A1	D. Did this happen 3 or more times in any 12-month period?	NO .... 1<br>YES 5
(31)<br>ALCFGNA3	AL17K. Have you ever passed out from drinking? That is, fallen into a deep sleep when you didn"t want to?	NO…………………………..1<br>YES…………………………5
	AL17 Have you ever had blackouts? I"m not talking about passing out, but drinking enough so that the next day you could not remember things you had said or done?	NO…….(SKIP TO AL18) .... 1<br>YES 5
	ONS/REC. How old were you the (first/last) time?	AGE ONS:____/____<br>ONS: 1 5<br>AGE REC:____/____<br>REC: 1 5
	B. How many blackouts have you had from drinking?<br>**IF DK, ASK B1. OTHERS SKIP TO AL18.**	___ _______TIMES
	1. Did you have 3 or more blackouts?	NO .... 1<br>YES 5

(10)	AL18 Did you ever feel you needed a drink just after you had gotten up (that is, before breakfast)?	NO .... 1<br>YES 5
ALCFGNB3	A. Did you ever take a drink just after you had gotten up?<br><br>**IF AL18 AND AL18A ARE BOTH CODED 1, SKIP TO AL19. OTHERS CONTINUE.**	NO .... 1<br>YES 5
	ONS/REC. How old were you the (first/last) time you took (needed) a drink just after you had gotten up?	AGE ONS:____/____<br>ONS: 1 5<br><br>AGE REC:____/____<br>REC: 1 5
	C. Did this happen 3 or more times?	NO .... 1<br>YES 5
(11)<br>ADICD1	AL19 At times when you couldn‟t drink, did you ever want to drink so badly that you couldn‟t think of anything else?	NO…….(SKIP TO AL20)……1<br>YES………………………….…5*
	ONS/REC. How old were you the (first/last) time?	AGE ONS:____/____<br>ONS: 1 5<br><br>AGE REC:____/____<br>REC: 1 5
(19)<br>ALCFGNB4	AL20 Have you ever drunk unusual things like mouthwash or cough syrup (like Nyquil) to get an effect, "buzzed," or drunk?	NO……(SKIP TO AL21) .... 1<br>YES 5
	ONS. How old were you the first time?	AGE ONS:____/____<br>ONS: 1 5

(36)<br>AD3RA4/B<br>AA3RA2<br>AA4A2	AL21 Have you drunk alcohol 3 or more times while taking medicine or drugs you knew were clearly dangerous to mix with alcohol?	NO ……(SKIP TO AL22) .... 1<br>YES……....................... ……5*
	A. What medication(s) or drug(s) did you use with alcohol 3 or more times when you knew they were dangerous to mix with alcohol?	
	1)____________________________________	CODE: __________
	2)____________________________________	CODE: __________
	3)____________________________________	CODE: __________
	4)____________________________________	CODE: __________
	**SPECIFY:** Why did you think this was dangerous?<br>____________________________________	
	ONS/REC. How old were you the (first/last) time you drank alcohol while taking medications or drugs you knew were dangerous to mix with alcohol?	AGE ONS: ___/___<br>ONS: 1 5<br>AGE REC:___/___<br>REC: 1 5
AA4A2	C. Did this happen 3 or more times in any 12-month period?	NO .... 1<br>YES 5
ADICD6	D. Did you have any bad effects from mixing alcohol and (DRUG/any of these drugs)?	NO .... 1<br>YES…….(SPECIFY)………....5*
	**SPECIFY: __________________________________**	

(30)	AL22K1 Have you ever driven a car when you were under the influence of alcohol?	NO…… ....1<br>YES 5
	K2. When you have been under the influence of alcohol, have you ever done something else that might have gotten you hurt; for example, riding your bike or skateboard, rollerblading, crossing against traffic, swimming, or anything else that might be dangerous?	NO ....1<br>YES…………………………5
	**IF BOTH CODED NO, SKIP TO AL25K.**<br>**OTHERS CONTINUE.**	
	ONS/REC. How old were you the (first/last) time any of these things happened?	AGE ONS:___/___<br>ONS: 1 5<br>AGE REC:___/___<br>REC: 1 5
AD3RA4/B<br>AA3RA2/B<br>ASP3RC7<br>ASP4A5	B. How many times (has/have) (this/these things) happened? **IF 3 OR MORE, MARK TALLY AND SKIP TO C. IF FEWER THAN 3, SKIP TO BOX AL22. IF DK, ASK B1.**	_______TIMES *
AD3RA4/B<br>AA3RA2/B<br>ASP3RC7<br>ASP4A5	1. Did (this/these things) happen 3 or more times?	NO….(SKIP TO BOXAL22)... 1<br>YES…………………………5*
AA4A2	C. Did (this/these things) happen 3 or more times in any 12-month period?	NO ....1<br>YES 5

BOX AL22 IF AL22K1=1, SKIP TO AL25K

(27)<br>ALCFGNC2	AL23 Have you ever been stopped or arrested for drunk driving?	NO…….(SKIP TO AL24) .... 1<br>YES 5
	ONS/REC. How old were you the (first/last) time?	AGE ONS:___/___<br>ONS: 1 5<br>AGE REC:___/___<br>REC: 1 5
AD3RA4/B<br>AA3RA2/B<br>ASP3RC7<br>ASP4A5	B. How many times has this happened? **IF 3 OR MORE, MARK TALLY AND SKIP TO C. IF FEWER THAN 3, SKIP TO AL24. IF DK, ASK B1.**	_______TIMES *
AD3RA4/B<br>AA3RA2/B<br>ASP3RC7<br>ASP4A5	1. Did this happen 3 or more times?	NO…….(SKIP TO AL24) .... 1<br>YES…………………………5*
AA4A3	C. Did this happen 3 or more times in any 12-month period?	NO ....1<br>YES 5

(27)<br>ALCFGNC2	AL24 When you have been drinking and driving, have you ever damaged your car or had an accident?<br>**COUNT ALL ACCIDENTS, EVEN IF NOT REPORTED TO THE POLICE.**	NO……(SKIP TO AL25K)...... 1<br>YES 5
ASP3RC7	ONS/REC. How old were you the (first/last) time?	AGE ONS:___/___<br>ONS: 1 5<br>AGE REC:___/___<br>REC: 1 5
AD3RA4/B<br>AA3RA2/B	B. How many times has this happened? **IF 3 OR MORE, MARK TALLY AND SKIP TO C. IF FEWER THAN 3, SKIP TO AL25K. IF DK, ASK B1.**	_______TIMES *
AD3RA4/B<br>AA3RA2/B	1. Did this happen 3 or more times?	NO…….(SKIP TO AL25K)...... 1<br>YES……………………….…5*
AA4A2	C. Did this happen 3 or more times in any 12-month period?	NO .... 1<br>YES 5

	AL25K. Have you ever gone to school (or to work) when drunk or hung over?	NO...(SKIP TO AL25K1)…..1<br>YES…………………………5
	ONS/REC. How old were you the (first/last) time?	AGE ONS:___/___<br>ONS: 1 5<br>AGE REC:___/___<br>REC: 1 5
	KB. Did this happen 3 or more times?	NO .... 1<br>YES…………………………5

(23) AD3RA4/B	AL25K1. Have you ever missed any school (or work) because you were drunk or hung over?	NO .... 1<br>YES 5
	K2. Have your grades gone down because of drinking or being hung over?	NO .... 1<br>YES 5
	K3. Have you ever dropped out of school (or quit a job) because of drinking?	NO .... 1<br>YES 5
	K4. Have you had any other problems at school (or at work) because you were drinking or hung over?	NO .... 1<br>YES 5
	K5. Have you had any problems at home with getting your chores done because of your drinking?	NO .... 1<br>YES 5
	**IF NO 5'S IN AL25K1-AL25K5, SKIP TO KC. OTHERS CONTINUE.**	
	ONS/REC How old were you the (first/last) time any of these things happened (NAME 5"s IN AL25K1-AL25K5)?	AGE ONS:___/___<br>ONS: 1 5<br>AGE REC:___/___<br>REC: 1 5
	KB1. Have (this/any of these things) (NAME 5"S IN AL25K1-AL25K5) happened 3 or more times in your lifetime?	NO…….(SKIP TO C)………1<br>YES…………………………..…5*
AA4A1	KB2. Did (this/these things) happen 3 or more times in any 12-month period?	NO .... 1<br>YES 5
	KC. Has anyone in your family told you they thought you were drinking too much?	NO……(SKIP TO AL26)…..1<br>YES…………………………5
	1. Did s/he really think you were drinking too much, or was s/he against anyone drinking or anyone as young as you drinking?	TOO YOUNG.…………………1*<br>TOO MUCH……………………2*<br>AGAINST ALL DRINKING…..3

		COL I			COL II	
	**FOR EACH 5 CODED IN AL26A.1-8, GET AGE ONSET AND ASK,** "Did this happen 3 or more times?" **CODE IN COL II.**	NO	YES	AGE ONS	NO	YES
(17) ALCFGND2 ALCFGND4 AD3RA6 AA3RA1/B	AL26A 1. Have your friends or anyone else outside your family told you that you were drinking too much?	1	5	___/___	1	5*
ALCFGND3 AD3RA6	2. Has anyone ever stopped being friends with you because of your drinking?	1	5	___/___	1	5*
ALCFGNC3 AD3RA6 AA3RA1/B	3. Did your drinking ever cause you to have problems at school (or work)?	1	5	___/___	1	5*
	4. When you‟ve been drinking, have there been times when you‟ve gotten really angry at someone and shouted or yelled at them?	1	5	___/___	1	5
ALCFGNC4	5. Did you ever hit things or throw something when you had been drinking?	1	5	___/___	1	5
ALCFGNC4	6. OMITTED					
ALCFGNC4	7. OMITTED					
ALCFGNC4	8. Have you gotten into fights where you shoved or hit someone when you were drinking?	1	5	___/___	1	5

IF ANY 5 IS CODED IN COL. I, CONTINUE. OTHERS SKIP TO AL28.

REC. How old were you the last time any of these experiences happened **(REVIEW SX CODED 5 IN COL. I)**?

AGE REC:____/____
REC: 1 5

AA4A4

C. Did any of these experiences happen 3 or more separate times in any 12-month period?

NO 1
YES 5

(24) AA4A4 AD3RA6

AL27 OMITTED

(28)<br>ALCFGNC1	AL28 Have you ever been arrested or held by the police even for a short time because of drinking (other than for drunk driving)?	NO…….(SKIP TO AL29) .... 1<br>YES 5
	ONS/REC. How old were you the (first/last) time?	AGE ONS:____/____<br>ONS: 1 5<br>AGE REC:____/____<br>REC: 1 5
AD3RA6	B. How many times has this happened? **IF 3 OR MORE, MARK TALLY AND SKIP TO C. IF FEWER THAN 3, SKIP TO AL29. IF DK, ASK B1.**	_______TIMES *
AD3RA6	1. Did this happen 3 or more times?	NO……(SKIP TO AL29) .... 1<br>YES………………………….…5*
AA4A3	C. Did this happen 3 or more times in any 12-month period?	NO .... 1<br>YES 5

(29)	AL29 Have you ever accidentally been seriously hurt when you were drinking? For example, have you ever had a bad fall or cut yourself badly, been hurt in a traffic accident, or anything like that?	NO…….(SKIP TO AL32)........ 1<br>YES…………………………5
	ONS/REC. How old were you the (first/last) time?	AGE ONS:____/____<br>ONS: 1 5<br>AGE REC:____/____<br>REC: 1 5
AD3RA4/B<br>ADICD6<br>AA3RA2	B. How many times has this happened? **IF 3 OR MORE, MARK TALLY AND SKIP TO C.**<br>**IF LESS THAN 3, SKIP TO D. IF DK, ASK B1.**	_______TIMES *
AD3RA4/B<br>ADICD6<br>AA3RA2	1. Did this happen 3 or more times?	NO……(SKIP TO D).............1<br>YES……………………….…..5*
AA4A2	C. Did this happen 3 or more times in any 12-month period?	NO .... 1<br>YES 5
	D. Did you go to an emergency room or see a doctor because of the accident(s)?	NO .... 1<br>YES 5

(16)	AL30 OMITTED	
(35)<br>ALCFGNA2	AL31 OMITTED	

(36)
AD3RA6
AD47
ADICD6
AA3RA1

AL32 Have there been times when you drank when you knew you had an illness or problem with your health that might be made worse by drinking?

NO……(SKIP TO AL33) 1
YES…………………………5*

SPECIFY: What illness or condition?

____ CODE: __________

____ CODE: __________

ONS/REC. How old were you the (first/last) time?

AGE ONS:___/___
ONS: 1 5

AGE REC:___/___
REC: 1 5

C. Did drinking make your illness or problem worse?

NO 1
YES 5

---

(37)

AL33 When you have been drinking alcohol, have any of the following things happened to you?

	NO	YES
1. Did you feel really depressed or not interested in things for more than a day (24 hours)?…………………………………………………………	1	5
2. OMITTED		
3. Did you have trouble concentrating on things or thinking clearly for more than a day (24 hours)?………………………………………………..	1	5
4. OMITTED		
5. Did you see or hear things that weren't really there for more than a day (24 hours)?	1	5

IF ALL ARE CODED 1, SKIP TO AL34. OTHERS CONTINUE.

AD3RA6
AD47
ADICD6
AA3RA1

6. Did you think that drinking had anything to do with problems like (NAME 5‟s in AL33.1-5)?

NO…..(SKIP TO AL34) 1
YES…………………………5

A. Did you continue to drink after you knew it caused you any of these problems?

NO…..(SKIP TO AL34) 1
YES…………………………5*

ONS/REC. How old were you the (first/last) time?

AGE ONS:___/___
ONS: 1 5

AGE REC:___/___
REC: 1 5

(25)
ALCFGND1

AL34 Have you ever thought that you were drinking too much? — NO…….(SKIP TO AL35)........1 / YES 5

A. How old were you the first time you thought that? — AGE ONS:___/___ ONS: 1 5

(26)
ALCFGND5

AL35 Have you ever felt guilty about drinking? — NO……(SKIP TO AL35K1)... 1 / YES..5

A. How old were you the first time you felt guilty about drinking? — AGE ONS:___/___ ONS: 1 5

Sometimes when people drink, they do things that they otherwise wouldn‟t.

AL35K1. When you have been drinking, have you ever had sex when you otherwise would not have?	NO.. 1<br>YES 5
K2. When you have been drinking, have you ever pressured someone to have sex with you?	NO……..(SKIP TO K3)……1<br>YES 5
A. Would you have done this if you had not been drinking?	NO.. 1<br>YES 5
K3. Has drinking ever made you careless about sex so that you didn‟t protect yourself or your partner against pregnancy or sexually transmitted diseases (like HIV)?	NO.. 1<br>YES 5
K4. When you have been drinking, have you taken any other risks that you normally wouldn‟t? For example, did you walk outside alone late at night or go into dangerous areas?	NO.. 1<br>YES 5
K5. Have you ever physically hurt someone else when you were drinking?	NO.. 1<br>YES 5
K6. When you‟ve been drinking, have you ever ridden in a car when the driver had been drinking or using drugs?	NO.. 1<br>YES 5
A. Would you have done this if you had not been drinking?	NO.. 1<br>YES 5

Cannabis Section

(1)

A. How many times have you used marijuana or hashish ? ___ ___ ___ ___ TIMES

1. **IF DK:** Would you say 11 or more times?
   NO……………………………1
   YES…………………………...5

2. **IF NO**: Would you say 5 or more times?
   NO……………………………1
   YES…………………………...5

IF MJ1A< 21 OR MJ1A1=1, SKIP TO C.

B. Did you ever use marijuana at least 21 times in a single year?
   NO……………………………1
   YES…………………………...5

C. From whom did you first get marijuana?
   SELF…………………………...1
   FRIEND/PEER………………...2
   MINOR FAMILY……………...3
   ADULT FAMILY………………4
   PARENT……………………….5
   OTHER….(SPECIFY)…………6

SPECIFY OTHER: ____________________________

____

D. From whom do/did you usually get marijuana?
   SELF…………………………...1
   FRIEND/PEER………………...2
   MINOR FAMILY……………...3
   ADULT FAMILY………………4
   PARENT……………………….5
   OTHER….(SPECIFY)…………6

SPECIFY OTHER: ____________________________

____

When you use cannabis, do you usually smoke it?	Yes	No
How many <u>times</u> have you smoked cannabis in the past	Day?	Number _________
	Week?	Number ________
	Month?	Number ________
	6 months?	Number ________
	Year?	Number ________

(2) MJ2 How old were you the first time you used marijuana? — AGE ONS: ___/___ *t* — ONS: 1 5

IF AGE ONS 15 OR LATER, SKIP TO REC.
OTHERS CONTINUE.

A. Did you use marijuana more than once before you were 15?
NO……………………………...1
YES……………………………5

REC. How old were you the last time you used marijuana? — AGE REC: ___/___ *t* — REC: 1 5
IF REC OVER 1 YEAR AGO, SKIP TO D.
OTHERS CONTINUE.

C. How many times did you use marijuana in the last 12 months? — __ __ __ TIMES
IF DK, ASK C1. IF MORE THAN 20 TIMES, SKIP TO MJ3. OTHERS SKIP TO D.

1. Did you use marijuana at least 21 times during the past 12 months?
NO……………………………..1
YES…………………………….5

D. Did you ever use marijuana at least once a week for a month or more?
NO……………………………...1
YES……………………………5

BOX MJ2 IF MJ1A IS LESS THAN 5 OR MJ1A2=1, SKIP TO DR1.
OTHERS CONTINUE.

---

(3)
DRFGNC

MJ3 What was the longest period of time you used marijuana almost every day? — ___ ___ ___ NUM

CODE UNITS:
DAYS…………………..1
WEEKS………………..2
MONTHS………………3
YEARS………………...4

IF NEVER, CODE 0 DAYS AND SKIP TO B.
IF LESS THAN 2 WEEKS, SKIP TO B.
OTHERS CONTINUE.

ONS/REC. How old were you the (first/last) time you used marijuana almost every day for at least two weeks?
AGE ONS: ___/___ *t* — ONS: 1 5
AGE REC:___/___ *t* — REC: 1 5

B. Please think about the period when you were using marijuana the most. During that period, how many days a month did you use marijuana? — ___ ___ DAYS

C. During that period of heaviest use, how much marijuana did you use on an average or typical day? — ___ ___ ___ NUM

CODE UNITS:
HITS…………………2
JOINTS/CIGS……….3
PIPEFULS…………...4

D. How old were you when that period started? — AGE: __ __ *t*

E. How long did that period last? — __ __ __ __ MONTHS

(4) DRFGNC	MJ4	Have you ever stayed high from marijuana for a whole day or more?	NO………(SKIP TO MJ5)……..1<br>YES…………………………...5
		ONS/REC. How old were you the (first/last) time you stayed high from marijuana for a whole day or more?	AGE ONS: ___/___<br>ONS: 1 5<br>AGE REC: ___/___<br>REC: 1 5

(5) DD3RA3/B DD45 DDICD5	MJ5	Did you ever have a period of a month or more when a lot of your time was spent using marijuana, getting marijuana, or trying to feel better after using marijuana?	NO…………………………...1<br>YES……………………………5*

(6) MJ6 Because of using marijuana, did any of the following things happen to you? **CODE IN COLUMN I.**

	COL. I NO	COL. I YES	COL. II NO	COL. II YES
1. Did you feel really depressed or not interested in things for more than a day (24 hours)?………..	1	5	1	5*
2. Did you have trouble concentrating on things or thinking clearly for more than a day (24 hours)?…	1	5	1	5*
3. OMITTED				
4. Did you talk to your friends or family less often o see them less often?…………………………..	1	5	1	5*
5. Did you hear, see, or smell things that weren't reall there?……………………………………	1	5	1	5*

IF ALL ARE CODED 1, SKIP TO MJ7

K. Did you think that using marijuana had anything to do with problems like these (NAME 5's in MJ6.1-5)?

NO……(SKIP TO MJ7)………1
YES……………………………5

DD3RA6/B
DD47
DDICD6
DA3RA1/B

FOR EACH 5 CODED IN COL.I, ASK A.

A. Did you keep using marijuana after you knew it caused this? **CODE IN COLUMN II.**

IF MJ6.4 IS CODED 1, SKIP TO MJ7. OTHERS CONTINUE.

DA4A4

B. Did you talk to your friends or family less often or see them less often 3 or more times in any 12-month period?

NO……………………………1
YES…………………………..5

(7)<br>DD3RA2<br>DD44<br>DDICD2	MJ7	Have you often wanted to stop or cut down on how much marijuana you were using?	NO……………………………1<br>YES…………………………..5*
DD3RA2		A. Have you ever tried to stop or cut down on marijuana but couldn't do it? **IF NEVER TRIED TO STOP/CUT DOWN, CODE NO.**<br><br>**IF NO, COULD STOP (OR NEVER TRIED), SKIP TO MJ8. OTHERS CONTINUE.**	NO, COULD STOP………….1<br>YES, COULD NOT STOP…..5*
DD44<br>DDICD2		B. Have you been unable to stop or cut down on marijuana 3 or more times?	NO……………………………1<br>YES…………………………..5

(8)<br>DD3RA1/B<br>DD43<br>DDICD2	MJ8	Have you often used more marijuana than you meant to, or used it for a longer time than you really wanted to?	NO……………………………1<br>YES…………………………..5*

(9)<br>DD3RA7<br>DD41<br>DDICD4	MJ9	Have you ever found that when you used the same amount of marijuana that you used to, it didn't affect you as much, or have you ever needed to use more marijuana to get high?	NO……………………………1<br>YES…………………………...5*

(10) MJ10 After you had stopped or cut down on marijuana, did any of the following things happen for most of the day for 2 days or longer? Did you.....

CODE IN COLUMN I.

	COL. I NO	COL. I YES	COL. II NO	COL. II YES
1. feel nervous, tense, restless or irritable?…………	1	5	1	5
2. have trouble sleeping?……………………………..	1	5	1	5
3. tremble or twitch?………………………………….	1	5	1	5
4. sweat or have a fever?……………………………..	1	5	1	5
5. feel sick to your stomach or throw up ?………….	1	5	1	5
6. have diarrhea or stomach aches?…………………..	1	5	1	5
7. have a big change in your appetite, either up or down that is, have a big change from your normal level?……………………………………………..	1	5	1	5

BOX MJ10A IF NO 5'S CODED IN MJ10.1-7, SKIP TO MJ11. OTHERS CONTINUE.

	A. Have you ever used marijuana to keep from having any of these problems or to make them go away?	NO….(SKIP TO BOX MJ10B)...1<br>YES…………………………..…..5
DD3RA9/B<br>DD42B<br>DDICD3	B. Did this happen 3 or more times?	NO……………………………1<br>YES…………………………...5*

BOX MJ10B IF ONLY ONE 5 CODED IN COL. I, SKIP TO MJ11. OTHERS CONTINUE.

DD3RA8
DD42A
DDICD3
DRFGNA

C. Did these problems ever happen together?
NO………(SKIP TO G)…...…..1
YES…………………………...5*

D. Which ones? **CODE IN COL. II**

DD3RB
DA3RA

E. How many times did you have problems like that (when they happened together)?
___ ___ ___ TIMES

DD3RB

F. What was the longest time these problems happened together?
___ ___ ___ DAYS

G. Did these problems interfere with your school (work) or home responsibilities?
NO…………………………….1
YES…………………………...5

---

(11)
ASP3RC7
ASP4A5

MJ11 Have you ever driven a car when you had been using marijuana?
NO…….…………………..…..1
YES…………………………...5

1. When you have been high from using marijuana, have you ever done something else that might have gotten you hurt; for example, riding your bike or skateboard, rollerblading, crossing against traffic, swimming, or anything else that might be dangerous?
NO…………………………….1
YES…………………………...5

IF BOTH MJ11 AND MJ11.1 CODED 1, SKIP TO MJ11B. OTHERS CONTINUE

DD3RA4/B
DA3RA2/B

A. Have you been in situations like this 3 or more times?
NO…….(SKIP TO B)…………..1
YES…………………………...5*

DA4A2

1. Did this happen 3 or more times in any 12-month period?
NO…………………………….1
YES…………………………...5

B. Have you ever accidentally been seriously hurt when you were using marijuana? For example, have you ever had a bad fall or cut yourself badly, been hurt in a traffic accident, or anything like that?
NO…….(SKIP TO MJ12)……...1
YES…………………………...5

DD3RA6/B
DDICD6
DA3RA1/B

C. Did this happen 3 or more times?
NO…….(SKIP TO D)…..……..1
YES…………………………...5*

DA4A2

1. Did this happen 3 or more times in any 12-month period?
NO…………………………….1
YES…………………………...5

D. Did you go to an emergency room or see a doctor because of the accident(s)?
NO…………………………….1
YES…………………………...5

(12)	MJ12	Did your marijuana use ever cause you to have problems with your friends or family?	NO……(SKIP TO MJ13)………1<br>YES…………………………...5
DA4A4		A. Did this happen 3 or more times in any 12-month period?	NO……………………………1<br>YES…………………………...5
DD3RA6<br>DA3RA1		B. Did you continue to use marijuana after you realized it was causing these problems?	NO……………………………1<br>YES…………………………...5*
	MJ13	Have you ever been arrested or had any other trouble with the police because of your marijuana use?	NO…….(SKIP TO MJ14)……...1<br>YES…………………………...5
DD3RA6		A. Did this happen 3 or more times?	NO…….(SKIP TO MJ14)……...1<br>YES…………………………...5*
DA4A3		1. Did this happen 3 or more times in any 12-month period?	NO……………………………1<br>YES…………………………...5
(14)<br>DD3RA4/B	MJ14	Has your being high on marijuana or recovering from its after-effects <u>often</u> interfered with your responsibilities at school (work) or home?	NO……..(SKIP TO MJ14K)…....1<br>YES…………………………...5*
DA4A1		A. Did this happen 3 or more times in any 12-month period?	NO……………………………1<br>YES…………………………...5
	MJ14K.	Have you ever gone to school (or to work) when you were high from using marijuana?	NO..…(SKIP TO MJ14KB1)…. 1<br>YES…………………………..5
		A. Did this happen 3 or more times?	NO……………………………1<br>YES…………………………...5

	MJ14KB1. Have you ever missed any school (or work) because you were high from using marijuana?	NO…………………………….1<br>YES…………………………...5
	2. Have your grades gone down because of using marijuana?	NO…………………………….1<br>YES…………………………...5
	3. Have you ever dropped out of school (or quit a job) because of using marijuana?	NO…………………………….1<br>YES…………………………...5
	4. Have you had any other problems at school (or at work) because of your marijuana use?	NO…………………………….1<br>YES…………………………...5
	5. Have you had any problems at home with getting your chores done because of your marijuana use?	NO…………………………….1<br>YES…………………………...5
	**IF NO 5'S IN MJ14KB1-MJ14KB5, SKIP TO MJ14K2A.**<br>**OTHERS CONTINUE.**	
	A. Has/Have (this/any of these things) (NAME 5'S IN MJ14KB1-B5) happened 3 or more times in your lifetime?	NO...…(SKIP TO MJ14K2A)..1<br>YES……………….……..….5*
	1. Did (this/these things) happen 3 or more times in any 12-month period?	NO…………………………….1<br>YES…………………………...5
DD3RA6<br>DA3RA1/B	MJ14K2A. Have your friends, family or anyone outside your family told you they thought you shouldn't be using marijuana?	NO……..…(SKIP TO B)…….1<br>YES…………..…………….5
	1. Did this happen 3 or more times?	NO…………………………….1<br>YES…………………………...5*
DD3RA6<br>DA3RA1/B	B. Has anyone ever stopped being friends with you because of your marijuana use?	NO……..…(SKIP TO C)…….1<br>YES…………..…………….5
	1. Did this happen 3 or more times?	NO……………..……..…….1<br>YES…….……………………..5*
	C. Have you ever thought that you were using marijuana too much?	NO…………………..…..….1<br>YES……………...………...5
(16)	MJ15 OMITTED.	
(15)<br>DDICD1	MJ16 At times when you couldn't use marijuana, did you ever want it so badly that you couldn't think of anything else?	NO……..(SKIP TO MJ17)……..1<br>YES…………………………...5*
	ONS/REC. How old were you the (first/last) time?	AGE ONS: ___/____<br>ONS: 1 5<br>AGE REC: ___/____<br>REC: 1 5

(13)	MJ17	Have you ever stopped doing things with any of your good friends because of your marijuana use? Or have you missed regular activities, like club meetings or sports practices because of using marijuana?	NO…….(SKIP TO MJ18)……...1<br>YES…………………………...5
DD3RA5/B<br>DD46<br>DDICD5		A. Has this happened 3 or more times, or for at least a month?	NO……………………………1<br>YES…………………………...5*

(17)	MJ18	Have you ever used marijuana together with one or more other drugs, including alcohol?<br><br>**IF YES, SPECIFY:** Let's list these drugs, beginning with the first you used together with marijuana.<br>1. ______________________________<br>2. ______________________________<br>3. ______________________________<br>4. ______________________________	NO………(SKIP TO MJ18K1)...1<br>ALCOHOL ONLY…………...3<br>YES……….(SPECIFY)……….5<br><br>CODE: ___ ___ ___<br>CODE: ___ ___ ___<br>CODE: ___ ___ ___<br>CODE: ___ ___ ___

Sometimes when people get high, they do things that they otherwise wouldn't.

MJ18K1. When you have been using marijuana, have you ever had sex when you otherwise would not have?	NO...1<br>YES...5
K2. When you have been using marijuana, have you ever pressured someone to have sex with you?	NO……..(SKIP TO K3)…….1<br>YES...5
A. Would you have done this if you had not been using marijuana?	NO...1<br>YES...5
K3. Has using marijuana ever made you careless about sex so that you didn't protect yourself or your partner against pregnancy or sexually transmitted diseases (like HIV)?	NO...1<br>YES...5
K4. When you have been using marijuana, have you taken any other risks that you normally wouldn't? For example, did you walk outside alone late at night or go into dangerous areas?	NO...1<br>YES...5
K5. Have you ever physically hurt someone else when you were using marijuana?	NO...1<br>YES …………………………..5
K6. When you've been high from using marijuana, have you ever ridden in a car when the driver had been drinking or using drugs?	NO...(SKIP TO BOX MJ19) .. 1<br>YES …………………………..5
A. Would you have done this if you had not been using marijuana?	NO...1<br>YES …………………………..5

BOX MJ22 IF 2+ SX, CONTINUE. OTHERS SKIP TO MJ23.

(22) MJ22 Since the age of (ONS), has there ever been a period of time lasting 3 months or longer when you did not use marijuana at all?

NO……(SKIP TO MJ23)…………..1
YES………………………………5

A. How many times have you stopped using marijuana for 3 months or longer?

___ ___ TIMES

When did the first period begin?
IF R HAD MORE THAN 4 ABSTINENT PERIODS, RECORD THE 4 LONGEST.

FROM __ __/__ __ __ __ MO YEAR	TO __ __/__ __ __ __ MO YEAR	*t*
FROM __ __/__ __ __ __ MO YEAR	TO __ __/__ __ __ __ MO YEAR	*t*
FROM __ __/__ __ __ __ MO YEAR	TO __ __/__ __ __ __ MO YEAR	*t*
FROM __ __/__ __ __ __ MO YEAR	TO __ __/__ __ __ __ MO YEAR	*t*

(18) MJ23 Have you ever talked about your marijuana use with a doctor or counselor?

NO………(SKIP TO MJ24)……1
YES…………………………...5

A. Did you talk with a . . .

	NO	YES
1. Psychiatrist………………………………………	1	5
2. Another medical doctor…………………………	1	5
3. Psychologist………………………………………	1	5
4. Another mental health professional………………	1	5
5. Minister, priest, rabbi or imam………………..…	1	5
6. Another professional……………………………..	1	5

IF YES, SPECIFY:______________________

ONS/REC. How old were you the (first/last) time you talked about your marijuana use with one of these professionals?

AGE ONS: ___/___
ONS: 1 5

AGE REC: ___/___
REC: 1 5

BOX MJ23 IF R ONLY SPOKE TO ONE PROFESSIONAL, SKIP TO MJ24. OTHERS CONTINUE.

C. To whom did you talk first?

___ ___ CODE

MJ24	Have you ever been in treatment for your marijuana use?	NO ……….(SKIP TO D)………1<br>YES…………………………...5

A. Were you ever treated at:

	NO	YES
1. NA or another self-help group?……..…………	1	5
2. an outpatient drug program, where you didn't stay overnight?………………………………….	1	5
3. an outpatient program for something other than marijuana?………………………………………	1	5
4. an inpatient drug program?……………………..	1	5
5. when you were an inpatient for medical problem caused by marijuana use?…………….	1	5
6. at any other place or program? ………………… **IF YES, SPECIFY**: ______________________________	1	5

B. How old were you the (first/last) time you were treated for a problem with marijuana?

AGE ONS: ___/____
ONS: 1 5

AGE REC: ___/____
REC: 1 5

C. Where were you <u>first</u> treated? **RECORD CODE (1-6) AND SKIP TO DR1.**

CODE: ___

D. Did you ever attend a self-help group (like NA) for your marijuana use?

NO…….(SKIP TO DR1)………1
YES…………………………..5

ONS/REC. How old were you the (first/last) time you attended a self-help group for your marijuana use?

AGE ONS: ___/___
ONS: 1 5

AGE REC: ___/___
REC: 1 5

Depression Section

Now I'm going to ask you some questions about your mood.

DP1	Have you ever had a period of time when everyday or nearly every day, you felt sad, depressed, or unhappy most of the time for two weeks?<br>(PROBE: What I mean is, these feelings didn't happen just for one or two days, but you felt like this most of the day, nearly every day for at least two weeks.)	NO..1<br>YES......................................5
DP2	Have you ever had a period of time when nothing seemed fun anymore? For example, you lost interest in things that you usually liked to do? These might be things like not wanting to hang out with your friends, not wanting to take part in your hobbies or after school activities.	NO………(SKIP TO A)............1<br>YES......................................5
	K1. Did these feelings of things not being fun anymore last most of the day, nearly every day for two weeks or more?	NO..1<br>YES......................................5
	A. Have you ever had a period of time when you felt irritable, crabby, or in a bad mood most of the time for two weeks or more?	NO…(SKIP TO BOX DP2)......1<br>YES......................................5
	K2. Did these feelings last most of the day, nearly every day for two weeks or more?	NO..1<br>YES......................................5

BOX DP2 IF DP1, DP2K1, and DP2K2 ALL CODED 1, SKIP TO SU1. OTHERS CONTINUE.

DP3 Think about the time in your life that stands out as the "worst" time in your life of feeling (MOOD ENDORSED ABOVE). I'm interested in periods that lasted at least two weeks.

How old were you when this worst period began?	AGE ONS:___/___
K1. **IF DK:** Did this worst period occur . . .<br>**READ OPTIONS UNTIL R ANSWERS AFFIRMATIVELY**	BEFORE 10 YRS OLD………...1<br>BEFORE 12 YRS OLD………...2<br>BEFORE 15 YRS OLD………...3<br>AFTER 15 YRS OLD…………4
A. How long did this worst period last?	___ ___ NUM<br>**CODE UNITS:**<br>DAYS....................................1<br>WEEKS..................................2<br>MONTHS...............................3<br>YEARS………………………4
1. **IF DK:** Was it two weeks or longer?	NO..1<br>YES…………………………..5
B. So you were ___ years old when this worst period ended?<br>**CORRECT AGE REC IF R DOES NOT CONFIRM**	AGE REC: ___/___

.

During this worst period when you were ____ years old . . .

(3B/4C) DEP4A1	DP4	A. Were you feeling depressed, sad, unhappy, or down for most of the day, nearly every day, for 2 weeks or more?	NO....1<br>YES....5*
(3C/4D) DEP4A1		B. Did you feel crabby or irritable most of the day, nearly every day, for 2 weeks or more?	NO....1<br>YES....5*
DEP4A2		C. Did most things stop being fun for you most of the day, nearly every day, for at least 2 weeks or more?	NO....1<br>YES....5*

BOX DP4 IF NO MOOD ENDORSED (DP4A, B, AND C=1), GO BACK TO DP3 AND CHECK FOR ANOTHER EPISODE. IF NO OTHER EPISODE, GO BACK TO DP1 AND/OR DP2 AND REVIEW ENDORSEMENTS.

During this worst period when you were _____ years old . . .

(9) DEP4A2	DP5	Were you a lot less interested in doing fun things or things you usually liked to do?	NO....1<br>YES....5*
(5) DEP4A3	DP6	A. Did you have a change in appetite? For example, did you eat a lot less than usual or a lot more than usual?	NO……(SKIP TO KB1)……..1<br>YES....5*
		1. Was this an increase, a decrease or did you have both?	INCREASE….....2<br>DECREASE… .....3<br>BOTH....4
DEP4A3		KB1. Did you lose weight when you were not trying to?	NO....1<br>YES....5*
		KB2. Did you gain weight when you were not trying to?	NO....1<br>YES....5*
		**IF NO TO BOTH KB1 AND KB2, SKIP TO DP7**	
		**IF YES TO BOTH KB1 AND KB2, ASK:**<br>B2. Which amount was bigger? The amount of weight you gained or the amount you lost?	GAINED….....2<br>LOST….....3
		KC. How much weight did you (lose/gain)? **IF BOTH, CODE THE MORE SIGNIFICANT CHANGE.**	___ ___ ___ LBS
		D. OMITTED	
		E. How long did it take you to (lose/gain) this amount of weight?	___ ___ NUM<br>**CODE UNITS:**<br>DAYS....1<br>WEEKS....2<br>MONTHS....3<br>YEARS....4

During this worst period when you were _____ years old . . .

(6)	DP7	Did you have more trouble sleeping than usual?	NO……….(SKIP TO F) .... 1<br>YES 5
		A. Did you have trouble falling asleep?	NO ………(SKIP TO C)........... 1<br>YES 5
DEP4A4		B. Did it take you an hour or more to fall asleep?	NO .... 1<br>YES…………………………..5*
DEP4A4		C. Did you wake up in the middle of the night and have trouble going back to sleep?	NO .... 1<br>YES…………………………..5*
		D. Did you wake up early in the morning, a lot earlier than usual, and have trouble falling back asleep?	NO ……..(SKIP TO F) .... 1<br>YES 5
DEP4A4		E. Was this at least one hour earlier than usual?	NO .... 1<br>YES..5*
DEP4A4		F. Did you sleep much more than usual?	NO .... 1<br>YES..5*
(7)<br>DEP4A5	DP8	Were you so fidgety or restless that you had a hard time keeping still?	NO…(SKIP TO DP9KA) .... 1<br>YES 5
		A. Was it so bad that other people noticed, or could they have noticed if they had been around?	NO .... 1<br>YES..5*
(8)<br>DEP4A5	DP9KA.	Did you talk more slowly than usual?	NO……(SKIP TO KB).............. 1<br>YES 5
		1. Was it so bad that other people noticed, or could they have noticed if they had been around?	NO .... 1<br>YES..5*
	KB.	Did you move more slowly than usual? For example, did you just feel slowed down?	NO…….(SKIP TO DP10) .... 1<br>YES…………………………...5
		1. Was it so bad that other people noticed, or could they have noticed if they had been around?	NO .... 1<br>YES..5*
(10)<br>DEP4A6	DP10	Did you feel tired most of the time, or feel that you had a lot less energy than usual?	NO .... 1<br>YES..5*
(11)<br>DEP4A7	DP11K.	Most of the time, did you feel that most things were your fault? For example, did you feel that problems at school, at home, or with your friends were mostly your fault?	NO .... 1<br>YES..5*
	KA.	Did you feel that you were a bad person?	NO .... 1<br>YES..5*

During this worst period when you were _____ years old . . .

(12) DEP4A7	DP12 Did you feel that you were a failure or a worthless person?	NO....1<br>YES....5*
(13) DEP4A8	DP13K Did you have a lot of problems thinking? For example, did your thoughts seem to come more slowly than usual?	NO....1<br>YES....5*
	KA. Did you have a lot of problems concentrating? For example, paying attention to your schoolwork or other things you were doing?	NO....1<br>YES....5*
(13) DEP4A8	DP14 Did you have a lot more problems than usual making decisions or making up your mind about things that you were doing? For example, did you have trouble making up your mind about every little thing?	NO....1<br>YES....5*
(14) DEP4A9	DP15 KA. Did things seem so bad that you wished you were dead?	NO....1<br>YES....5*
DEP4A9	KB. Did you think a lot about being dead or dying?	NO…….(SKIP TO D)……….1<br>YES....5*
DEP4A9	C. Did you make a plan about how you might kill yourself?	NO....1<br>YES....5*
DEP4A9	D. Did you try to kill yourself?	NO....1<br>YES....5*

.

(16)	DP16 You told me about the following things that were happening to you when you were feeling the worst. **(REVIEW SX)**. **HAND R DEPRESSION TALLY SHEET.**	
	A. Did you feel (MOOD ENDORSED IN DP4A-C) and have some of these problems nearly every day, for at least 2 weeks?	NO....1<br>YES....5
(19)	DP17 Did you see a doctor or some other professional person to help you with these problems?	NO………(SKIP TO DP18).....1<br>YES....5

A. Did you talk to a:

	NO	YES
1. Psychiatrist……	1	5
2. Psychologist……	1	5
3. Social worker……	1	5
4. School counselor……	1	5
5. Other medical doctor……	1	5
6. Nurse……	1	5
7. Minister, priest, rabbi or imam……	1	5
8. Another professional: ____________	1	5

(20) DP18 During this worst period, were you prescribed any medicine to help you with these feelings (or were you already taking medicine)?

NO…….(SKIP TO DP19) 1
YES 5

A. Do you know the name of the medicine?

1. ____ CODE: ___ ___ ___

2. ____ CODE: ___ ___ ___

3. ____ CODE: ___ ___ ___

DP19 Did all these feelings during that worst period cause problems for you with:

1. Your family? For example, did you argue more with them, or did you just not feel like doing things with them? Anything else like that?

   NO…………………………….…...1
   YES…………………………..5

2. Your friends? For example, did you stay away from them, or have problems being with them?

   NO…………………………….…...1
   YES…………………………..5

3. Did your grades go down or did you have trouble getting your work done, or did you have more problems with your teachers? **CODE N/A IF NOT IN SCHOOL DURING EPISODE.**

   NO…………………………….…...1
   YES…..………………………..…5
   N/A…..………………………...7

4. Did you have trouble getting your work done at your job, or did you have more problems with your co-workers or boss? **CODE N/A IF NOT WORKING DURING EPISODE.**

   NO…………………………….…...1
   YES…………………………..5
   N/A….………………………...7

5. Any other problems during this worst period of time?

   NO…………………………….…...1
   YES…………………………..5

Sometimes people have episodes of depression that follow the death of a loved one, heavy drinking or drug use, a change in smoking behavior, a change in medication, or a serious illness (or childbirth).

BOX DP20 IF TB3=1, SKIP TO BOX DP21. OTHERS CONTINUE.

DP20 During the 6 weeks (that would be a month and a half) before this worst period of time started, did you quit or cut down on smoking?

NO.. 1
YES.. 5+

	**BOX DP21 IF AL1a=1 OR AL6 < 3, SKIP TO BOX DP22.**

(31) DP21 During the 6 weeks (that would be a month and a half) before this worst period of time started, how many days a week did you usually drink alcohol? ____ DAYS

1. **IF DK:** Did you usually drink alcohol at least 2 days a week?
   NO…(SKIP TO BOX DP22)... 1
   YES……………………………5

2. **IF YES**: Did you usually drink alcohol at least 4 days a week?
   NO……................................. 1
   YES…………………………...5

**BOX DP21A IF 0 OR 1 DAYS, SKIP TO BOX DP22. OTHERS CONTINUE.**

A. On the days when you drank, how many drinks would you usually have? **HAND R CARD AL1.** ___ ___ ___ DRINKS

1. **IF DK AND MALE**: Would you usually have 5 or more drinks a day?
   IF DK AND FEMALE: Would you usually have 3 or more drinks a day?
   NO………………………….... 1
   YES…………………………..5

DEP4D

**BOX DP21B**	**CODE SILENTLY:** TYPICALLY 3+ (FEMALE) OR 5+ (MALE) DRINKS FOR 4+ DAYS PER WEEK?	NO…………………………..1<br>YES…………………………5+

B. During the 6 weeks before this worst period began, what was the largest number of drinks you had in one day? ___ ___ ___ DRINKS

1. **IF DK:** Did you drink at least 5 drinks in a single day?
   NO…(SKIP TO BOX DP22)... 1
   YES…………………………...5

**BOX DP21C**	**IF 4 DRINKS OR FEWER (DP21b <5 OR DP21b1=1), SKIP TO BOX DP22.**<br><br>**CODE SILENTLY:**<br>TYPICALLY 5+ DRINKS AT LEAST TWICE A WEEK? (DP21a $\geq$ 5 OR DP21a1=5)	NO…………………………...1<br>YES...(SKIP TO BOX DP22)...5+

DEP4D C. Did you drink at least 5 drinks 2 or more times a week during the 6 weeks before this worst period began?
NO... 1
YES.. 5+

E & F OMITTED

.

(30) DEP4D

DP22 During the 6 weeks before this worst period began, did you use cannabis or street drugs, such as cocaine or ecstasy, or use any prescription drugs when they were not prescribed or more than prescribed?

NO……(SKIP TO DP23) 1
YES………(SPECIFY)............. 5

List THE THREE USED MOST.

1. ______________________________ CODE: ___ ___ ___
2. ______________________________ CODE: ___ ___ ___
3. ______________________________ CODE: ___ ___ ___

B. During that time, on average, how many days per week did you take (DRUG)?

DRUG 1: ___ DAYS
DRUG 2: ___ DAYS
DRUG 3: ___ DAYS

BOX DP22B CODE SILENTLY:
WAS ANY DRUG USED 4 OR MORE DAYS PER WEEK ON AVERAGE?

NO…………………………...1
YES…………………………5+

C. What is the average number of times you used (DRUG) on those days you used?

DRUG 1: ___ ___ AVG
DRUG 2: ___ ___ AVG
DRUG 3: ___ ___ AVG

D. During the 6 weeks before this episode began, what was the largest number of times you used (DRUG) in one day?

DRUG 1: ___ ___ MAX
DRUG 2: ___ ___ MAX
DRUG 3: ___ ___ MAX

E. On how many days during that 6-week period did you use (DRUG) **(# IN D)** times in a day? **(6 WEEKS = 42 DAYS)**

DRUG 1: ___ ___ DAYS
DRUG 2: ___ ___ DAYS
DRUG 3: ___ ___ DAYS

F & G OMITTED

---

(29) DEP4D

DP23 During the 6 weeks before this worst period began, did you start or change the dose of a prescription medicine, such as steroids or asthma medications?

NO…….(SKIP TO DP24) 1
YES………(SPECIFY)........... 5+

1. ______________________________ CODE: ___ ___ ___
2. ______________________________ CODE: ___ ___ ___

A & B OMITTED

---

(28)<br>DEP4E	DP24	Did this worst period of time begin within 6 months of learning about the death of someone very close to you?<br><br>A. What was this person's relationship to you?<br><br>___________________________________<br><br>B & C OMITTED	NO…….(SKIP TO DP25) .... 1<br>YES……………………...…5+
(26)<br>DEP4D	DP25	Did this worst period of time begin within the 6 weeks that followed a serious illness that you had? For example, thyroid disease, bad problems with diabetes, a tumor or a need for an operation, or a very bad infection?<br><br>**SPECIFY:**________________________________<br><br>A & B OMITTED	NO…(SKIP TO BOX DP26)... 1<br>YES……..(SPECIFY)............. 5+<br><br>CODE: ___ ___ ___

**BOX DP26**	**IF R IS MALE, SKIP TO DP27x.**<br>**IF R IS FEMALE, BUT HAS NEVER BEEN PREGNANT (DM14K=1), SKIP TO DP27x.**<br>**OTHERS CONTINUE.**

(27)	DP26K	Have you ever given birth to a child, had a miscarriage, or an abortion?	NO….(SKIP TO DP27x) .... 1<br>YES 5
		1. Did this worst period begin around the time of a childbirth, miscarriage, or abortion?	NO….(SKIP TO DP27x) .... 1<br>YES 5
		A. Did this worst period of time begin between the 2 weeks before to 6 weeks after the (birth/miscarriage/abortion)?<br><br>B & C OMITTED	NO.. 1<br>YES... 5+
(32F/33F)	DP27x	How many periods of time lasting 2 weeks or longer (such as the one(s) we have been talking about) have you had in your whole lifetime? This includes the one(s) we already talked about?<br><br>**IF ONLY 1 EPISODE AND R SAW A PROFESSIONAL (DP17=5), SKIP TO DP31C.**<br>**IF ONLY 1 EPISODE AND R DID NOT SEE A PROFESSIONAL (DP17=1), SKIP TO DP34.**	___ ___ ___ NUMBER
(34)	ONS/REC.	How old were you the (first/last) time you had a period of depression lasting 2 weeks or longer?	AGE ONS: ___/___<br>AGE REC: ___/___<br>REC: 1 5
		B. **IF DK ONSET:** Did your first episode of depression which lasted at least 2 weeks occur . . .<br><br>**BEGINNING WITH "BEFORE 10 YRS OLD," READ OPTIONS UNTIL R ANSWERS AFFIRMATIVELY.**	BEFORE 10 YRS OLD……….1<br>BEFORE 12 YRS OLD……….2<br>BEFORE 15 YRS OLD……….3<br>AFTER 15 YRS OLD…………4

	**BOX DP27 IF ANY + IN DP20-DP26, CONTINUE. OTHERS SKIP TO DP28.**

(32) DP27 Have you ever had another time in your life when you felt down, sad, or unhappy, or lost interest in things you usually liked to do, or felt irritable most of the time? This would be a time that did not follow the death of a loved one, did not follow daily (or almost daily) use of alcohol or drugs, did not occur when you quit or cut down on smoking, did not follow a serious physical illness, and did not follow a change in prescription medicines (**IF FEMALE:** and was not around the time of childbirth, miscarriage, or abortion)? Again, I'm interested in episodes which lasted at least 2 weeks.

NO…….(SKIP TO DP28) 1
YES 5

IF MORE THAN ONE ADDITIONAL CLEAN EPISODE, HAVE R PICK THE MOST SEVERE ONE. 2 WEEK DURATION IS CRUCIAL.

C. How old were you then? ___/___ AGE

KA1. **IF DK:** Did your first episode of depression which lasted at least 2 weeks occur . . .
BEGINNING WITH "BEFORE 10 YRS OLD," READ OPTIONS UNTIL R ANSWERS AFFIRMATIVELY.

BEFORE 10 YRS OLD……..... 1
BEFORE 12 YRS OLD…… 2
BEFORE 15 YRS OLD…… 3
AFTER 15 YRS OLD………...4

B. During this period when you were ___ years old:
COUNT ONLY IF MORE THAN USUAL

BOX A Depressed

B1. Were you sad, unhappy, or depressed a lot more than usual?
NO……................................... 1
YES……………………………5

B2. Were you a lot more irritable or crabby than usual?
NO……................................... 1
YES……………………………5

BOX B Loss of Interest

B3. Did you lose interest in things you usually liked to do?
NO……................................... 1
YES……………………………5

	**BOX DP27B IF NO MOOD ENDORSED (DP27B1, B2, AND B3=1), GO BACK TO DP27A AND CHECK FOR ANOTHER EPISODE. IF NO OTHER EPISODE, GO BACK TO DP27 AND REVIEW ENDORSEMENT.**

BOX C Appetite/ Weight

B4. OMITTED
A. OMITTED

B5. Did you lose weight or gain weight when you were not trying to?
NO……....(SKIP TO 6)…..….1
YES…………………………..5

KA. Did you lose weight?
NO……................................... 1
YES…………………………..5

KB. Did you gain weight?
NO……................................... 1
YES……………………… …5

BOX D Sleeping

B6. Did you have trouble sleeping?
NO……................................... 1
YES…………………………...5

B7. Did you sleep too much?
NO……................................... 1
YES…………………………...5

During this period when you were ___ years old:

BOX E Restless/ Slowed Down	B8. Were you a lot more restless than usual?	NO……………………………1<br>YES…………………………..5
	B9. Did you feel a lot more slowed down than usual?	NO……………………………1<br>YES…………………………..5
BOX F Tired	B10. Were you tired all the time, or did you feel that you had very little energy?	NO…………………………..…1<br>YES……………….………….5
BOX G Guilt	B11. Did you feel guilty about things or bad about yourself?	NO……………………………1<br>YES…………………………..5
BOX H Thinking	B12. Did you have difficulty thinking or concentrating?	NO……………………………1<br>YES…………………………..5
BOX I Thoughts of Dying	B13. Did you think about dying?	NO……………………………1<br>YES…………………………..5
	B14. Did you think about committing suicide?	NO………(SKIP TO B)………1<br>YES…………………………..5
	A. Did you make a suicide plan?	NO……………………………1<br>YES…………………………..5
	B. Did you try to kill yourself?<br>1. What did you do?<br>SPECIFY: ________________________________	NO..(SKIP TO BOX DP27B)..1<br>YES…………………………..5

BOX DP27B IF FIVE OR MORE BOXES CODED 5 IN DP27 B1-14, CONTINUE. OTHERS SKIP TO DP28.

KC. Did these problems happen nearly every day for two weeks or longer? **REVIEW SX ENDORSED IN DP27B.5-14.**	NO……(SKIP TO DP28)……1<br>YES…………………………..5
D. Did they happen at the same time as (MOOD ENDORSED IN DP27B.1-3)?	NO…….(SKIP TO DP28)…..1<br>YES…………………………..5
KE. Did (MOOD AND SX) happen nearly every day for two weeks or longer?	NO…………………………..1<br>YES…………………………..5

DP28 What is the longest period of time you've ever had when, for 2 weeks or more, you felt (depressed, sad, unhappy, crabby/irritable, or uninterested in things you usually liked)?

___ ___NUM

CODE UNITS:
DAYS..1
WEEKS.......................................2
MONTHS.....................................3
YEARS 4

BOX DP30 ASK DP30. 1-5 ONLY IF DP19. 1-5 CODED NO.

DP30 Have any of your depressive episodes ever caused problems for you with:

	NO	YES	NA
1. family	1	5	
2. friends	1	5	
3. work	1	5	7
4. school	1	5	7
5. other situations	1	5	

CODE N/A IF NOT WORKING OR IN SCHOOL DURING ANY OF DEPRESSIVE EPISODES.

BOX DP31 IF DP17=5, SKIP TO DP31C.

DP31 Has there ever been a time when you wanted to talk to a doctor or other professional about these periods of depression?

NO…….(SKIP TO DP32) 1
YES 5

A. Did you do it?

NO……..(SKIP TO DP32) 1
YES 5

B. Did you talk to a:

	NO	YES
1. Medical doctor/Psychiatrist.……………………………..	1	5
2. Psychologist/Social worker/Counselor……………………	1	5
3. Nurse…………………………………………….….	1	5
4. Minister, priest, rabbi or imam……………………………	1	5
5. Another professional……………………………………..	1	5

IF YES, SPECIFY: ___________________________

C. How old were you the (first/last) time you talked to a health professional about your depression?
IF ONLY 1 EPISODE (DP27x=1), SKIP TO DP34.
IF DP17=5, SKIP TO BOX DP33.
OTHERS CONTINUE.

AGE ONS: ___/___

AGE REC: ___/___
REC: 1 5

DP32 Were you ever treated by a doctor, or other professional for depression?

NO….(SKIP TO DP34) 1
YES 5

BOX DP33 IF DP18=5, SKIP TO DP34.

DP33	Were you ever prescribed medicine for depression?<br>A. What medication(s)?<br>1. ____<br>2. ____<br>3. ____	NO….(SKIP TO DP34) .... 1<br>YES .... 5<br><br>CODE: ___ ___ ___<br>CODE: ___ ___ ___<br>CODE: ___ ___ ___
DP34	Were you ever hospitalized for depression?<br><br>For how long?	NO…….(SKIP TO SU1) .... 1<br>YES…………………………...5<br><br>___ ___NUM<br>**CODE UNITS:**<br>DAYS.. 1<br>WEEKS.. 2<br>MONTHS.................................. 3<br>YEARS 4

Suicide Section

Now I would like to ask you some (more) questions about times when you might have been feeling really down and felt like nothing would ever go right with you.

SU1. Have you ever thought about killing yourself?

NO......... (SKIP TO SU2)......... 1
YES.. 5

A. (Earlier you told me that you'd thought about taking your own life) Did those thoughts last for at least 7 days in a row?

NO.. 1
YES.. 5

IF DP15C=5 OR DP27B14A=5, SKIP TO SU1C

B. Did you have a plan about how you might kill yourself? (Did you actually consider a way to take your life?)

NO.....(SKIP TO ONS/REC) ... 1
YES.. 5

C. (Earlier you told me you had a plan for how you might kill yourself) What were you going to do?
SPECIFY:___________________________________

ONS/REC. How old were you the (first/last) time you had these thoughts about killing yourself?

AGE ONS: ___/___

AGE REC:____/____
REC: 1 5

IF DP15D=5 OR DP27B14B=5, SKIP TO SU2A

SU2. Have you ever <u>tried</u> to kill yourself?

NO .... (SKIP TO SU12)...... 1
YES.. 5

A. (Earlier you said that you'd tried to kill yourself) How many times (did you try to kill yourself)?

_______TIMES

ONS/REC. How old were you the (first/last) time?

AGE ONS:____/____
ONS: 1 5

AGE REC:___/_____
REC: 1 5

SU3. Can you tell me what happened when you tried to kill yourself?
IF MORE THAN 1, ASK ABOUT THE MOST SERIOUS ATTEMPT. CODE SILENTLY: TYPE OF METHOD INTENDED.

CODE: ____

1. Fire gun.
2. Crash car.
3. Carbon monoxide poisoning.
4. Cut wrists or stab self.
5. Take pills.
6. Jump from height.
7. Jump in front of train/car/vehicle.
8. Strangulation, choking, suffocation, hanging, drowning.
9. Other or combination.

SU3A. How close did you come to killing yourself? CODE: ___

CODE SILENTLY: DEGREE OF COMPLETION

2. Put self in vicinity (e.g., brought gun/pills into room, walked into train station).
3. Stopped short of completing act (held gun/pills, stood on edge of platform, sat in car).
4. Attempted act (jumped, pulled trigger, swallowed pills).

B. Did you really want to die?

NO……………………………1
YES…………………………..5

C. **CODE SILENTLY: INTENT** CODE: ___

1. Unclear (no information or not sure)
2. Denies intent
3. Reports minimal intent
4. Reports significant intent with some ambivalence
5. Very severe/extreme intent to die

D. Afterwards, were you sorry that you didn't die?

NO... 1
YES.. 5

SU4. How old were you then? AGE:

SU5. Did you see a doctor for medical treatment after you tried to kill yourself?

NO…….(SKIP TO SU6A)…..1
YES.. 5

SU6. Were you admitted to a hospital afterwards because you hurt yourself (for medical reasons)?

NO... 1
YES.. 5

A. Were you admitted to a hospital afterwards because of (the thoughts you were having/trying to kill yourself)—not because you hurt yourself?

NO... 1
YES.. 5

B. Did you see a doctor or counselor because you (had these thoughts/made a plan to kill yourself/tried to kill yourself)?

NO... 1
YES.. 5

SU8. Did you think you would die from what you had done?

NO... 1
MAYBE.................................... 3
YES.. 5

SU9. Did you try to kill yourself:

		NO	YES
1.	While feeling sad or down?	1	5
2.	While feeling extremely good or high?	1	5
3.	After you had been drinking?	1	5
4.	After using drugs?	1	5
5.	While having strange thoughts or experiences, or while seeing visions?	1	5
6.	Under other circumstances	1	5

SU12. (Other than when you were trying to kill yourself,) did you ever hurt yourself on purpose, for example, by cutting or burning yourself?	NO.. 1<br>YES... 5
A. How many times?	_______TIMES
ONS/REC. How old were you the (first/last) time?	AGE ONS:___/___<br>ONS: 1 5<br>AGE REC:___/___<br>REC: 1 5

**FOR ANY AGE ONS THAT R SAYS "DON'T KNOW", ASK CORRESPONDING DK QUESTION: Was it when you were:**	**UNDER 13……………..……….1**<br>**13-14…………………………….2**<br>**15-17…………………………….3**
**A/D PROBE: Did this ever happen when you were under the influence of alcohol (or drugs)?**<br>**[IF YES:] Did this only happen when you were under the influence of alcohol (or drugs)?**	**ONLY ALC/DRUGS = 3**<br>**NEVER ALC/DRUGS = 5**<br>**BOTH = 6**

Many kids do things that can get them into trouble with their parents or teachers. I am going to ask you about different ways of getting into trouble.

AS1 Have you ever skipped or "ditched" school for an entire day?
NO……….(SKIP TO AS1C)….….1
YES…………………………….5

A. Has this happened three or more times?
NO………(SKIP TO AS1C)..…….1
YES……………………...……5

ASP3RB1
CD3RA5
CD4A15
CDICD18
FGNASPA

ONS. How old were you the first time you skipped school?
MARK TALLY IF AGE ONSET BEFORE 13.
AGE ONS: ___/___ A
AGE ONS DK: 1 2 3

C. Have you ever cut classes?
NO……(SKIP TO AS2A)……..1
YES…………………………….5

D. Have you done this 3 or more different days?
NO……(SKIP TO AS2A)……..1
YES…………………………….5

E. How old were you the first time you cut classes?
MARK TALLY IF AGE ONSET BEFORE 13.
AGE ONS: ___/___ A
AGE ONS DK: 1 2 3

AS2A Have you ever been suspended from school? This would include in-school and out-of-school suspensions.
NO……….(SKIP TO AS2B)…......1
YES…………………………..….5

FGNASPA

ONS. How old were you the first time?
AGE ONS: ___/___
AGE ONS DK: 1 2 3

AS2B Have you ever been expelled from school?
NO……….(SKIP TO AS3)……...1
YES…………………………...…5

FGNASPA

ONS. How old were you the first time?
AGE ONS: ___/___
AGE ONS DK: 1 2 3

FGNASPB	AS3	Have you ever run away from home overnight?	NO………(SKIP TO AS4)……….1<br>YES……………………………5
		A. Why did you run away?	**CODE SILENTLY:**<br>AVOID PHYSICAL ABUSE…..2<br>AVOID SEXUAL ABUSE……..3<br>OTHER………………………....4
ASP3RB2<br>CD3RA2<br>CD4A14<br>CDICD19		B. Have you run away overnight more than once?<br>**MARK TALLY A ONLY IF AS3A=4**	NO………(SKIP TO C)..…………1<br>YES…………………………......5 A
		How old were you the (first/last) time you ran away from home overnight?	AGE ONS: ___/___<br>AGE ONS DK: 1 2 3
		**CODE AGES AND THEN SKIP TO AS4.**	AGE REC: ___/___
ASP3RB2<br>CD3RA2		C. After you ran away, did you return home?<br>**MARK TALLY A ONLY IF AS3A=4**	NO……..…(SKIP TO C2)………..1 A<br>YES……………….….………...5
CD4A14<br>CDICD19		1. When you ran away, how long did you stay away from home? **MARK TALLY IF AWAY FOR 7 OR MORE DAYS AND IF AS3A=4.**	__ __ __ DAYS A
		How old were you?	AGE ONS: ___/___<br>AGE ONS DK: 1 2 3
	AS4	Have you ever stayed out late at night without permission, either for 2 or more hours after the curfew your parents set or all night without permission?	NO…….(SKIP TO AS5)……..…1<br>YES……………………..……..…5
		A. Has this happened 3 or more times?	NO…..….(SKIP TO AS5)…...….1<br>YES…………………..……..…5
CD4A13<br>CDICD12		How old were you the first time?<br>**MARK TALLY IF AGE ONS LESS THAN 13.**	AGE ONS: ___/___ A<br>AGE ONS DK: 1 2 3
	AS5	Did you ever sneak out of the house at night after your parents thought you had gone to bed?	NO……....(SKIP TO AS6)…..….1<br>YES…………………………….…5
		A. Has this happened 3 or more times?	NO……...(SKIP TO AS6)…..….1<br>YES…………………………….5
CD4A13<br>CDICD12		How old were you the first time?<br>**MARK TALLY IF AGE ONS LESS THAN 13.**	AGE ONS: ___/___ A<br>AGE ONS DK 1 2 3

(7)<br>CD3RA11<br>CD4A2	AS6K1	Have you ever started fights with your brothers or sisters, not just screaming or arguing, but fights where you hit each other?	NO………....(SKIP TO K2)…...1<br>ALC/DRUGS ONLY…………3<br>YES, CLEAN…………………5<br>BOTH A/D & CLEAN………..6
		1. Has this happened 3 or more times?	NO……….…(SKIP TO K2)…..1<br>YES……………………..…...…..5 A
ASP3RB3<br>FGNASPF	A.	At what age did you (first/last) start fights with siblings?	
		1. UNRELATED TO ALC/DRUGS.	AGE ONS: ___/___
			AGE ONS DK: 1 2 3
		2. IN CONTEXT OF ALC/DRUGS.	AGE ONS A/D:
			AGE ONS A/D DK: 1 2 3
		. RECENCY.	AGE REC: ___/___
CD3RA13			
CD3RA11<br>CD4A2<br>CDICD10	KB.	Have you ever started physical fights with persons other than your brothers and sisters?	NO….(SKIP TO BOX AS6D)…..1<br>ALC/DRUGS ONLY…………3<br>YES, CLEAN…………………5<br>BOTH A/D & CLEAN………..6
		1. Has this happened 3 or more times?	NO…(SKIP TO BOX AS6D)…1<br>YES………………...…...……5 A
ASP3RB3<br>FGNASPF	C.	At what age did you (first/last) start fights with persons other than siblings?	
		1. UNRELATED TO ALC/DRUGS.	AGE ONS: ___/___
			AGE ONS DK: 1 2 3
		2. IN CONTEXT OF ALC/DRUGS.	AGE ONS A/D: ___/___
			AGE ONS A/D DK: 1 2 3
		RECENCY.	AGE REC: ___/___

BOX AS6D IF R IS UNDER 15 YEARS OLD, SKIP TO AS7. OTHERS CONTINUE.

ASP3RC3
ASP4A4
FGNASPF
DSICDB4

D. (Even though you didn't start fights,) since your 15th birthday, have you been in 3 or more physical fights? **DO NOT COUNT FIGHTS WITH SIBLINGS UNLESS SOMEONE WAS HURT.**

NO………..(SKIP TO AS7)……. . 1
ALC/DRUGS ONLY.………..….3
YES, CLEAN……..………..…..5
BOTH A/D & CLEAN.…………6

E. How old were you the (first/last) time?
1. UNRELATED TO ALC/DRUGS.

AGE ONS: ___/___

AGE ONS DK: 1 2 3

2. IN CONTEXT OF ALC/DRUGS.

AGE ONS A/D: ___/___

AGE ONS A/D DK: 1 2 3

RECENCY.

AGE REC: ___/___

---

(12)
CDICD3

AS7 Have you often challenged your parents, teachers, or other adults by refusing to do things they asked you to do, just because you didn't want to? For example, refusing to do things like chores or running errands, refusing to participate in class, or not behaving well?

NO………..(SKIP TO AS8)……1
YES……………………………5

How old were you the first time?

AGE ONS: ___/___

AGE ONS DK: 1 2 3

---

(13)
CDICD1
FGNASPF

AS8 When things do not go your way, have you ever gotten so angry that you've thrown things, broken things, or laid on the ground and screamed?

NO……….(SKIP TO AS9)…….1
YES……………………………5

How old were you the first time?

AGE ONS: ___/___

AGE ONS DK: 1 2 3

---

(14)
CD4A1
CDICD22

AS9 Do people complain that you are often a bully, deliberately hurting, threatening, or being mean to other people?

NO……….(SKIP TO AS10)…...1
YES…………………………...5 A

How old were you the (first/last) time?

AGE ONS: ___/___

AGE ONS DK: 1 2 3

AGE REC: ___/___

---

(15)
CD3RA8
CD4A5
CDICD14

AS10 Have you ever hurt or injured a pet or any other animal on purpose?
SPECIFY: ________________________________

NO…….(SKIP TO AS11)………1
YES…...…(SPECIFY)………..….5 A

A. How many times?

___ ___ TIMES

ASP3RB6

How old were you the (first/last) time?

AGE ONS: ___/___

AGE ONS DK: 1 2 3

AGE REC: ___/___

(4)<br>CD3RA3	AS11 Of course everybody tells lies or makes up stories once in awhile. Have you often lied or made up stories a lot?	NO……………………………..1<br>ALC/DRUGS ONLY………….3<br>YES, CLEAN…………………..5 A<br>BOTH A/D & CLEAN………..6 A
CD3RA3<br>CD4A11<br>CDICD9	A. Have you often gotten into trouble a lot because people said you were lying?	NO……………………………..1<br>ALC/DRUGS ONLY………….3<br>YES, CLEAN…………………..5 A<br>BOTH A/D & CLEAN………..6 A
	B. Have you ever used an alias or a false name? **EXCLUDE MINORS USING FALSE ID TO BUY ALCOHOL OR ENTER A BAR.**	NO….(SKIP TO BOX AS11)…...1<br>ALC/DRUGS ONLY………….3<br>YES, CLEAN…………………..5<br>BOTH A/D & CLEAN………..6
CD3RA3<br>CD4A11<br>CDICD9	1. Did you ever do this to take advantage of a person or a situation?	NO……………………………..1<br>YES……………………………5 A

BOX AS11 IF AS11, AS11A AND ASB.1 ARE ALL CODED 1, SKIP TO AS11D. OTHERS CONTINUE.

ASP3RB10<br>ASP3RC6<br>ASP4A2	How old were you when you (first/last) (told a lot of lies/used an alias to take advantage of someone)?	AGE ONS: ___/___
	1. UNRELATED TO ALC/DRUGS.	AGE ONS DK: 1 2 3
		AGE ONS A/D: ___/___
	2. IN CONTEXT OF ALC/DRUGS.	AGE ONS A/D DK: 1 2 3
	RECENCY.	AGE REC: ___/___
	D. Have you ever tricked or conned someone into giving you something or getting them to do something for you--like telling your parents you need extra money for a school project when you really want the money to buy something else?	NO……(SKIP TO AS12)……..1<br>ALC/DRUGS ONLY………….3<br>YES, CLEAN…………………..5<br>BOTH A/D & CLEAN………..6
	E. Have you done that 3 or more times?	NO……………………….…...1<br>YES…………………………...5 A
	F. How old were you the (first/last) time?	
	1. UNRELATED TO ALC/DRUGS.	AGE ONS: ___/___
		AGE ONS DK: 1 2 3
	2. IN CONTEXT OF ALC/DRUGS.	AGE ONS A/D: ___/___
		AGE ONS A/D DK: 1 2 3
	RECENCY.	AGE REC: ___/___

CDICD5<br>DSICDB6	AS12 When something goes wrong that is your fault, do you <u>usually</u> try to get out of it by blaming others?	NO……….(SKIP TO AS13)……..1<br>ALC/DRUGS ONLY…………..3<br>YES, CLEAN…………………..5<br>BOTH A/D & CLEAN………..6
	How old were you the (first/last) time?<br>1. UNRELATED TO ALC/DRUGS.	AGE ONS: ___/___<br>AGE ONS DK: 1 2 3
	2. IN CONTEXT OF ALC/DRUGS.	AGE ONS A/D: ___/___<br>AGE ONS A/D DK: 1 2 3
	RECENCY.	AGE REC: ___/___
CD3RA3<br>CD4A11<br>CDICD9	AS13 Have you <u>often</u> cheated on schoolwork, on exams, in games, or anything like that, or have other people often said that you cheated?	NO………(SKIP TO AS14)……1<br>YES……………………………..5 A
CD3RA3<br>CD4A11<br>CDICD9	A. OMITTED	
ASP3RB10<br>ASP3RC6<br>ASP4A2	How old were you the (first/last) time?	AGE ONS: ___/___<br>AGE ONS DK: 1 2 3<br>AGE REC: ___/___

(5)<br>CD3RA1<br>CD4A12<br>CDICD17<br>DSICDB2	AS14	Have you <u>more than once</u> stolen money or things from your family, friends, or relatives? **COUNT ONLY IF MORE THAN A FEW DOLLARS.**	NO………(SKIP TO B)……... 1<br>ALC/DRUGS ONLY………..3<br>YES, CLEAN………………..5 A<br>BOTH A/D & CLEAN………6 A
ASP3RB11		A. How old were you the (first/last) time?<br>1. UNRELATED TO ALC/DRUGS.	AGE ONS: ___/___<br>AGE ONS DK: 1 2 3
		2. IN CONTEXT OF ALC/DRUGS.	AGE ONS A/D: ___/___<br>AGE ONS A/D DK: 1 2 3
		RECENCY.	AGE REC: ___/___
CD3RA1<br>CD4A12<br>CDICD17<br>DSICDB2		B. Have you <u>more than once</u> shoplifted from stores or secretly stolen from other people? (NO CONFRONTATION)	NO…….(SKIP TO D)…………1<br>ALC/DRUGS ONLY………..3<br>YES, CLEAN………………..5 A<br>BOTH A/D & CLEAN………6 A
ASP3RB11		C. How old were you the (first/last) time?<br>1. UNRELATED TO ALC/DRUGS.	AGE ONS: ___/___<br>AGE ONS DK: 1 2 3
		2. IN CONTEXT OF ALC/DRUGS.	AGE ONS A/D: ___/___<br>AGE ONS A/D DK: 1 2 3
		RECENCY.	AGE REC: ___/___
CD3RA1<br>CD4A12<br>CDICD17<br>DSICDB2		D. Have you <u>more than once</u> signed someone else's name on a check or used a credit card without permission?	NO…..…(SKIP TO AS15)…....1<br>ALC/DRUGS ONLY………..3<br>YES, CLEAN………………..5 A<br>BOTH A/D & CLEAN………6 A
ASP3RB11		E. How old were you the (first/last) time?<br>1. UNRELATED TO ALC/DRUGS.	AGE ONS: ___/___<br>AGE ONS DK: 1 2 3
		2. IN CONTEXT OF ALC/DRUGS.	AGE ONS A/D: ___/___<br>AGE ONS A/D DK: 1 2 3
		RECENCY.	AGE REC: ___/___
ASP3RC2<br>ASP4A1		F. OMITTED	

(18)<br>CD3RA6<br>CD4A10<br>CDICD23<br>DSICDB2	AS15	Have you ever broken into someone else's home, car, or building (not because you were locked out)?	NO……..(SKIP TO AS16)…….1<br>ALC/DRUGS ONLY………..3<br>YES, CLEAN………………..5 A<br>BOTH A/D & CLEAN………6 A
		K. How many times have you done something like that?	__ __ __TIMES
		**IF DK:** 1. Was it . . .	1 TIME ………………..………..1<br>2 TIMES…………………….……..2<br>3-5 TIMES…………………………3<br>6-10 TIMES…………………………4<br>11 OR MORE TIMES……...………5
ASP3RC2<br>ASP4A1		How old were you the (first/last) time?<br>1. UNRELATED TO ALC/DRUGS.	AGE ONS: ___/___
			AGE ONS DK: 1 2 3
		2. IN CONTEXT OF ALC/DRUGS.	AGE ONS A/D: ___/___
			AGE ONS A/D DK: 1 2 3
		RECENCY.	AGE REC: ___/___
		B. OMITTED	
(19)<br>CD3RA12<br>CD4A6<br>CDICD20<br>DSICDB2	AS16	Have you ever taken money or property from someone else by threatening them or using force, like mugging them (using a knife or gun), snatching a purse, or stealing a wallet?	NO….(SKIP TO AS17)………1<br>ALC/DRUGS ONLY…………3<br>YES, CLEAN.………………..5 A<br>BOTH A/D & CLEAN……….6 A
ASP3RC2<br>ASP4A1		K. How many times have you done something like that?	__ __ __TIMES
		**IF DK:** 1. Was it . . .	1 TIME ………………..………..1<br>2 TIMES…………………….……..2<br>3-5 TIMES…………………………3<br>6-10 TIMES…………………………4<br>11 OR MORE TIMES………………5
ASP3RB12		How old were you the (first/last) time?<br>1. UNRELATED TO ALC/DRUGS.	AGE ONS: ___/___
			AGE ONS DK: 1 2 3
		2. IN CONTEXT OF ALC/DRUGS.	AGE ONS A/D: ___/___
			AGE ONS A/D DK: 1 2 3
		RECENCY.	AGE REC: ___/___
		B. OMITTED	

(16)<br>CD3RA4<br>DSICDB2	AS17	Have you ever set a fire on purpose that you were not supposed to?	NO……..(SKIP TO AS18)…….1<br>ALC/DRUGS ONLY………..3<br>YES, CLEAN………………..5<br>BOTH A/D & CLEAN………6
CD4A8<br>CDICD16		A. Did you do this with the intention to damage property?	NO……………………………1<br>YES…………………………..5 A
ASP3RB9<br>ASP3RC2<br>ASP4A1<br>DSICDB2		How old were you the (first/last) time?<br>1. UNRELATED TO ALC/DRUGS.	AGE ONS: ___/___<br>AGE ONS DK: 1 2 3
		2. IN CONTEXT OF ALC/DRUGS.	AGE ONS A/D: ___/___<br>AGE ONS A/D DK: 1 2 3
		3. RECENCY.	AGE REC: ___/___
ASP3RC2		C. Has this happened 3 or more times?	NO……………………………1<br>YES…………………………..5
(6)<br>CD3RA7<br>CD4A9<br>CDICD15<br>DSICDB2	AS18	Have you ever damaged someone's property on purpose other than by fire setting? For example, breaking windows, destroying computer files, spray painting graffiti, throwing rocks at cars, or tearing clothes.<br><br>**SPECIFY:** ______________________________<br>____________________________________	NO……..(SKIP TO AS19)…….1<br>ALC/DRUGS ONLY………..3<br>YES, CLEAN………………..5 A<br>BOTH A/D & CLEAN………6 A
		K. How many times have you done something like that?	__ __ __TIMES
		**IF DK**: 1.Was it . . .	1 TIME ……………….…………1<br>2 TIMES………………….……..2<br>3-5 TIMES…………………………3<br>6-10 TIMES…………………………4<br>11 OR MORE TIMES………………5
ASP3RB8		How old were you the (first/last) time?<br>1. UNRELATED TO ALC/DRUGS.	AGE ONS: ___/___<br>AGE ONS DK: 1 2 3
		2. IN CONTEXT OF ALC/DRUGS.	AGE ONS A/D: ___/___<br>AGE ONS A/D DK: 1 2 3
		RECENCY.	AGE REC: ___/___
		**IF AGE ONS IS LESS THAN 15, ASK B.**<br>**OTHERS SKIP TO BOX AS18.**	

AS18B. Did you more than once damage someone's property before you turned 15?

NO……………………………1
YES…………………………..5

BOX AS18 IF AGE REC <15, SKIP TO AS19. IF AGE REC > 15, OR IF AGE REC UNKNOWN AND AGE ONS ≥ 15, ASK D.

NOTE: AS18C CODED AUTOMATICALLY IN SAS CONVERSION ONLY.

C. Since your 15th birthday, have you damaged someone else's property on purpose?

NO……..(SKIP TO AS19)…….1
YES…………………………..5

ASP3RC2
ASP4A1

D. Have you done this 3 or more times since your 15th birthday?

NO……………………………1
YES…………………………..5

---

(9)
CD3RA13
CD4A4
CDICD13

AS19 When you weren't fighting, have you ever physically hurt another person on purpose—like twisting their arm behind their back so it really hurt, holding their head under water for a long time, burning them, or anything like that?

NO……..(SKIP TO AS20)…….1
ALC/DRUGS ONLY………..3
YES, CLEAN………………..5 A
BOTH A/D & CLEAN………6 A

SPECIFY:________________________________

ASP3RB7
ASP3RC3
ASP4A4
FGNASPF
DSICDB4

K. How many times have you done something like that?

__ __ __TIMES

IF DK: 1. Was it . . .

1 TIME ……………….…………1
2 TIMES……………………….……..2
3-5 TIMES…………………………3
6-10 TIMES…………………………4
11 OR MORE TIMES…………...…5

How old were you the (first/last) time?

1. UNRELATED TO ALC/DRUGS.

AGE ONS: ___/___

AGE ONS DK: 1 2 3

2. IN CONTEXT OF ALC/DRUGS.

AGE ONS A/D: ___/___

AGE ONS A/D DK: 1 2 3

RECENCY.

AGE REC: ___/___

(8)<br>CD3RA10<br>CD4A3<br>CDICD11<br>DSICDB4<br>FGNASPF	AS20 Have you ever used a weapon like a stick, gun, or a knife to hurt someone?	NO……..(SKIP TO AS21)…….1<br>ALC/DRUGS ONLY………..3<br>YES, CLEAN…………………5 A<br>BOTH A/D & CLEAN………6 A	
ASP3RB4<br>ASP3RC3<br>ASP4A4	How old were you the (first/last) time?		
	1. UNRELATED TO ALC/DRUGS.	AGE ONS:	___/___
		AGE ONS DK:	1 2 3
	2. IN CONTEXT OF ALC/DRUGS.	AGE ONS A/D:	___/___
		AGE ONS A/D DK:	1 2 3
	RECENCY.	AGE REC:	___/___
(26)<br>CD3RA9<br>CD4A7<br>CDICD21<br>DSICDB4	AS21 Have you ever made someone do sexual things with you when s/he didn't want to?	NO…(SKIP TO BOX AS22)….1<br>ALC/DRUGS ONLY………..3<br>YES, CLEAN………………..5 A<br>BOTH A/D & CLEAN………6 A	
ASP3RB5<br>ASP3RC3<br>ASP4A4	How old were you the (first/last) time?		
	1. UNRELATED TO ALC/DRUGS.	AGE ONS:	___/___
		AGE ONS DK:	1 2 3
	2. IN CONTEXT OF ALC/DRUGS.	AGE ONS A/D:	___/___
		AGE ONS A/D DK:	1 2 3
	RECENCY.	AGE REC:	___/___

BOX AS22 IF 3 OR MORE MARKS ON ASP TALLY, CONTINUE.

OTHERS SKIP TO AS23.

B. When you were doing things like (REVIEW SX ENDORSED), did any of the following things happen?

	NO	YES
1. Did your grades go down?…………………………………………	1	5
2. Did your teachers get angry with you a lot?…………………….....	1	5
3. Did your teachers often tell you that you had a bad attitude?……...	1	5
4. Did you feel very sad?……………………………………………..	1	5
5. Did you lose friends?………………………………………………	1	5
6. Did you ever lose a boyfriend or girlfriend?……………………….	1	5
7. Did your parents get really angry with you a lot?………………….	1	5
8. Were you grounded or not allowed to do something you really wanted to do?……………………………………………….………..	1	5
9. Were you sent to live somewhere else (including foster care)?……	1	5
10. Were you sent to a counselor? …………………………………….	1	5
IF YES, SPECIFY REASON:____________________________		
11. Were you arrested?……………………………………………….	1	5
12. Were you sent to juvenile court?…………………………………	1	5
IF YES, SPECIFY: _____________________________________		
13. Anything else?……………………………………………………	1	5
IF YES, SPECIFY: _____________________________________		

(21) AS23

Variables	Have you ever....	NO	ALC/ DRUG ONLY	YES CLEAN	BOTH A/D & CLEAN
ASP3RC2 ASP4A1 DSICDB2	1. Used an ATM or Credit card that you did not own?	1	3	5	6
ASP3RC2 ASP4A1 DSICDB2	2. Received, sold, or bought stolen goods (fenced), sold drugs, or "run numbers" (illegally gambled)?	1	3	5	6
ASP3RC2 ASP4A1 DSICDB2 FGNASPG	3. Been paid for having sex with someone?	1	3	5	6

a. **IF YES (3, 5, OR 6):** Were you paid with drugs?

NO……………………………1
YES…………………………...5

ASP3RC2 ASP4A1 DSICDB2 FGNASPG

4. OMITTED

IF ALL CODED 1, CONTINUE.
OTHERS SKIP TO B.

ASP3RC2 ASP4A1

A. Have you ever done anything else that you could have been arrested for, even if you weren't (other than using drugs or underage drinking)?

NO….(SKIP TO BOX AS25).…1
ALC/DRUGS ONLY………..3
YES, CLEAN………………..5
BOTH A/D & CLEAN………6

SPECIFY:________________________________

B. Did this happen 3 or more times?

NO……………………………1
YES…………………………..5

How old were you the (first/last) time?

1. UNRELATED TO ALC/DRUGS. — AGE ONS: ___/___
   AGE ONS DK: 1 2 3
2. IN CONTEXT OF ALC/DRUGS. — AGE ONS A/D: ___/___
   AGE ONS A/D DK: 1 2 3

RECENCY. — AGE REC: ___/___

AS24 OMITTED

(27) ASP3RC3 ASP4A4 DSICDB4 FGNASPE	AS26	Have you ever been accused of child abuse or been the subject of a complaint on the child abuse hotline?	NO……..(SKIP TO AS27)……1<br>ALC/DRUGS ONLY………..3<br>YES, CLEAN………………..5<br>BOTH A/D & CLEAN………6
		How old were you the (first/last) time?<br>1. UNRELATED TO ALC/DRUGS.	AGE ONS: ___/___<br>AGE ONS DK: 1 2 3
		2. IN CONTEXT OF ALC/DRUGS.	AGE ONS A/D: ___/___<br>AGE ONS A/D DK: 1 2 3
		RECENCY.	AGE REC: ___/___
(28) ASP3RC3 ASP4A4 DSICDB4 FGNASPF	AS27	Deleted Question.	–

	**BOX AS28 IF R IS LESS THAN 15 YEARS OLD, SKIP TO AS29. OTHERS CONTINUE.**	

(10) ASP3RC7 ASP4A5	AS28	Have you ever had a traffic ticket for a moving violation (things like speeding, running a red light, causing an accident, or driving without a license)?	NO……..(SKIP TO AS29)……1<br>ALC/DRUGS ONLY………..3<br>YES, CLEAN………………..5<br>BOTH A/D & CLEAN………6
FGNASPC DSICDB2		A. How many tickets have you received in your life?<br>**IF DK, ASK A1. OTHERS SKIP TO B.**	___ ___ TICKETS
FGNASPC DSICDB2		1. Was it at least 4?	NO……………………………1<br>YES…………………………..5
		How old were you the (first/last) time?<br>1. UNRELATED TO ALC/DRUGS.	AGE ONS: ___/___<br>AGE ONS DK: 1 2 3
		2. IN CONTEXT OF ALC/DRUGS.	AGE ONS A/D: ___/___<br>AGE ONS A/D DK: 1 2 3
		RECENCY.	AGE REC: ___/___

(11)<br>ASP3RC2<br>ASP4A1<br>DSICDB2	AS29	Have you ever been arrested for anything? **DO NOT COUNT DRUNK & DISORDERLY CONDUCT OR PUBLIC INTOXICATION.**	NO…(SKIP TO BOX AS30)..…1<br>ALC/DRUGS ONLY………..3<br>YES, CLEAN………………..5<br>BOTH A/D & CLEAN………6
		How old were you the (first/last) time you were arrested?<br>1. UNRELATED TO ALC/DRUGS.	AGE ONS: ___/___
			AGE ONS DK: 1 2 3
		2. IN CONTEXT OF ALC/DRUGS.	AGE ONS A/D: ___/___
			AGE ONS A/D DK: 1 2 3
		RECENCY.	AGE REC: ___/___
FGNASPC		B. How many times have you been arrested?	___ ___ TIMES
FGNASPC		C. Have you ever been convicted of a felony?	NO……………………………1<br>YES…………………………..5
		D. Have you ever spent time in jail for something other than using drugs or alcohol?	NO…(SKIP TO BOX AS30).….1<br>YES…………………………..5
DSICDB5		E. Since you got out of jail have you ever been arrested for things other than using drugs or alcohol?	NO……………………………1<br>YES…………………………..5
	**BOX AS30 IF R IS LESS THAN 15, SKIP TO AS34. OTHERS CONTINUE.**		
	AS30K	Have you ever had a part-time or full-time job?	NO……..(SKIP TO AS34)…….1<br>YES…………………………..5
(29)<br>ASP3RC1C<br>ASP4A3<br>DSICDB2<br>FGNASPD	AS30	Have you quit 3 or more jobs before having another job lined up?<br>A. OMITTED	NO…………………………….1<br>ALC/DRUGS ONLY………..3<br>YES, CLEAN………………..5<br>BOTH A/D & CLEAN………6
(30)<br>ASP3RC1B<br>ASP4A6<br>DSICDB2	AS31	On any job you have had, have you frequently been late or absent?<br>A. OMITTED	NO……..(SKIP TO AS34)……1<br>ALC/DRUGS ONLY………..3<br>YES, CLEAN………………..5<br>BOTH A/D & CLEAN………6
		How old were you the (first/last) time?<br>1. UNRELATED TO ALC/DRUGS.	AGE ONS: ___/___
			AGE ONS DK: 1 2 3
		2. IN CONTEXT OF ALC/DRUGS.	AGE ONS A/D: ___/___
			AGE ONS A/D DK: 1 2 3
		RECENCY.	AGE REC: ___/___
		C. Have supervisors complained to you 3 or more times or were you ever fired because you were frequently late or absent?	NO……………………………1<br>YES…………………………..5

	AS32-AS33 OMITTED		
	Now I'm going to ask you a few more questions about your relationships and your sexual experiences.		
(22)<br>DSICDB3	AS34	Have you ever had a close personal friendship or love relationship that lasted continuously for more than 1 year?	NO……………………………1<br>YES…………………………..5
	AS35K	Have you ever had sexual intercourse voluntarily?	NO……..(SKIP TO AS38)....….1<br>YES…………………………..5
(23)	AS35	How old were you when you first had sexual intercourse (voluntarily)?	AGE ONS: ___/___
FGNASPG		A. How many sexual partners have you had in your life? **IF 1, SKIP TO AS37. IF 2-9, SKIP TO AS36. OTHERS CONTINUE.**	___ ___ ___ NUMBER
FGNASPG		B. Have you ever had sex with 10 different people within a single year?	NO……………………………1<br>YES…………………………..5
(24)	AS36	Have you ever been unfaithful to any person in a romantic or love relationship; that is, had an affair or one-night stand?	NO……..(SKIP TO AS37)…….1<br>ALC/DRUGS ONLY…………3<br>YES, CLEAN…………………5<br>BOTH A/D & CLEAN……….6
(25)<br>DSICDB3<br>FGNASPE		A. Did this happen 3 or more times?	NO……………………………1<br>YES…………………………..5
ASP3RC9<br>DSICDB3		B. Have you ever been faithful to 1 person for more than 1 year (that is, when you did not have any other sexual relationships)? **IF NEVER HAD A 1-YEAR RELATIONSHIP, CODE 7.**	NO, NEVER FAITHFUL……1<br>YES, WAS FAITHFUL………5<br>N/A……………………………7
ASP3RC7<br>ASP4A5	AS37	Have you more than once had unprotected sex (without a condom) with someone you believed could give you a disease, or when you had a disease that could be spread that way?	NO……………………………1<br>ALC/DRUGS ONLY…………3<br>YES, CLEAN…………………5<br>BOTH A/D & CLEAN……….6

ASP3RC7
ASP4A5

AS38 Have you often taken chances where you or someone else might get physically hurt? For example, playing with fireworks or guns in a reckless manner?

NO……………………………1
ALC/DRUGS ONLY………..3
YES, CLEAN………………..5
BOTH A/D & CLEAN………6

SPECIFY: ______________________________

IF R IS LESS THAN 15, SKIP TO BOX AS38

ASP3RC7
ASP4A5

A. Have you often taken chances when driving--like racing a train to a crossing, or drag racing?

NO……………………………1
ALC/DRUGS ONLY………..3
YES, CLEAN………………..5
BOTH A/D & CLEAN………6

SPECIFY: ______________________________

BOX AS38 IF AS38 AND AS38A ARE BOTH CODED 1, SKIP TO AS39. OTHERS CONTINUE.

How old were you the (first/last) time?

1. UNRELATED TO ALC/DRUGS.
   AGE ONS: ___/___
   AGE ONS DK: 1 2 3
2. IN CONTEXT OF ALC/DRUGS.
   AGE ONS A/D: ___/___
   AGE ONS A/D DK: 1 2 3

RECENCY. AGE REC: ___/___

---

(17)

AS39 Was there ever a time when you really enjoyed tricking people to the point that you would often go out of your way to fool them?

NO……..(SKIP TO AS40)…….1
ALC/DRUGS ONLY………..3
YES, CLEAN………………..5
BOTH A/D & CLEAN………6

How old were you the (first/last) time?

1. UNRELATED TO ALC/DRUGS.
   AGE ONS: ___/___
   AGE ONS DK: 1 2 3
2. IN CONTEXT OF ALC/DRUGS.
   AGE ONS A/D: ___/___
   AGE ONS A/D DK: 1 2 3

RECENCY. AGE REC: ___/___

ASP3RC6
ASP4A2

B. OMITTED

(35)<br>ASP3RC10<br>ASP4A7<br>DSICDB1	AS40	Have you often ignored the feelings of others in order to do what you wanted?	NO…………………………….…1<br>ALC/DRUGS ONLY………..3<br>YES, CLEAN………………..5<br>BOTH A/D & CLEAN………6
(35)<br>ASP3RC3<br>CDICD6/7<br>DSICDB6	AS41	Have you often felt irritable, angry, or resentful (that is, you frequently lost your temper, or it was easy to annoy you or make you mad)?	NO…………………………….…1<br>ALC/DRUGS ONLY………..3<br>YES, CLEAN………………..5<br>BOTH A/D & CLEAN………6
(37)<br>CDICD5<br>DSICDB6	AS42	Have you often felt that others were to blame for your troubles or your mistakes?	NO…………………………….…1<br>ALC/DRUGS ONLY………..3<br>YES, CLEAN………………..5<br>BOTH A/D & CLEAN………6
	AS43	OMITTED	

AT4A1 AD1 Let me ask you about what you were like, say from age 6 to age 10. This would be from the first through fourth grade. During this period, was there ever a period of at least 6 months when . . . .

Variable	Item	6 MONTH DURATION NO	YES
AT4A1G	1. you were <u>always</u> losing things like assignments, notebooks, homework or other things you needed?	1	5
	A1. Did your mom or the teacher complain that you were always losing things?	1	5
AT4A1A	2. you made a lot of careless mistakes in your schoolwork or jobs at home because you rushed through them without checking?	1	5
AT4A1I	3. you <u>often</u> forgot what you were supposed to be doing or what you had planned to do?	1	5
AT4A1C	4. people would tell you something and it seemed as though you weren't listening?	1	5
AT4A1B	5. you <u>quickly</u> lost interest in games you were playing or in work you were doing?	1	5
AT4A1H	6. you were easily distracted from schoolwork or from other things you were doing because every little thing would grab your attention?	1	5
	A6. When something little was going on around you, did you often stop what you were doing and pay attention to that?	1	5
AT4A1F	7. you <u>disliked</u> or <u>avoided</u> doing schoolwork or homework because it was so hard to pay attention?	1	5
AT4A1E	8. you <u>often</u> had difficulty organizing your things and activities?	1	5
AT4A1D	9. you found it really hard to follow through on instructions even when you knew what you were supposed to do and meant to do it?	1	5
ADHD3RA8	10. you often started doing one thing and then changed to something else without finishing the first thing?	1	5

BOX AD1 IF THREE OR MORE 5'S CODED IN AD1.1-10, CONTINUE. OTHERS SKIP TO AD6.

REVIEW SX AS NEEDED

AD2 Did these difficulties cause problems for you . . .

	NO	YES
1. at school?	1	5
2. at home?	1	5
3. with your friends?	1	5
4. other places?	1	5

IF ALL CODED 1, SKIP TO AD3.

A. Did any of these difficulties keep on causing problems for you (at school/at home/with friends/in other places) for a month or longer?

NO 1
YES 5

AD3 How old were you the (first/last) time you had any of these problems? **REVIEW SX CODED 5 IN AD1.1-10**

AGE ONS: ___/___
ONS: 1 5

1. **IF DK ONSET:** Was it before you were 7 years old (that would be before the first or second grade)?

NO 1
YES………………………5

AGE REC:____/____
REC: 1 5

IF AGE ONS WITHIN 2 YEARS OF AGE REC, SKIP TO AD4.

A. Between (AGE ONS) and (AGE REC), was there any full year when you were not having special difficulty paying attention or completing things?

NO 1
YES 5

AD4 Did you or a parent ever talk to a doctor about your trouble completing things or paying attention?

NO 1
YES 5

AD5 Were you ever given any medicine to help you with the problems that we've been talking about, such as (**REVIEW SX**)?

NO .... (SKIP TO AD6) 1
YES ..... (SPECIFY) 5

ONS. How old were you when you first started taking the medicine(s)?

_______AGE

DRUG 1. ______________________________________ CODE: ___________

DRUG 2. ______________________________________ CODE: ___________

DRUG 3. ______________________________________ CODE: ___________

IF DK NAME OF MEDS, ASK: Do you think you took Ritalin, Adderall, Concerta, Dexadrine or Cylert?

NO…………………………1
YES 5
DK……………………...9

A. Are you still taking the medicine?

NO 1
YES ..... (SKIP TO C)......... 5

B. How old were you when you stopped taking the medicine(s)?

_______AGE

C. After you started taking the medicine, did these problems start to get better?

NO……………………...1
YES………………………5

			6 MONTH DURATION	
AT4A2	AD6	Now I'd like to ask you some more questions about what you were like, say from age 6 to age 10, or first to fourth grade. During this period, was there ever at least 6 months when	NO	YES
AT4A2C	1.	you were always climbing on things or running around, when you were not supposed to?	1	5
AT4A2E	2.	you kept going all the time?	1	5
AT4A2D	3.	you had a really hard time doing things quietly, like reading a book, either by yourself or in school?	1	5
AT4A2A	4.	you often fidgeted and squirmed in your seat?	1	5
AT4A2B	5.	you got up from your seat a lot when you were not supposed to, for example at dinner, school, or religious services?	1	5
AT4A2F	6.	you were very talkative?	1	5
	A6.	Did people tell you that you talked all the time?	1	5
AT4A2G	7.	you often gave the answer to a question before someone had finished asking it?	1	5
AT4A2I	8.	you often interrupted other people when they were talking?	1	5
AT4A2H	9.	you had more trouble than most children with waiting for your turn, or waiting in line?	1	5
AT4A2I	10.	you often tried to butt into games or other activities without being asked?	1	5
ADHD3RA14	11.	you often did careless things like running into the street without looking or running into things because you didn't look where you were going?	1	5

BOX AD6 **IF THREE OR MORE 5'S CODED IN AD6.1-11, CONTINUE. OTHERS SKIP TO OD1.**

REVIEW SX AS NEEDED

AD7 Did these difficulties cause problems for you . . .

	NO	YES
1. at school?	1	5
2. at home?	1	5
3. with your friends?	1	5
4. other places?	1	5

IF ALL CODED 1, SKIP TO AD8

A. Did these behaviors ever cause serious problems for you (at school/at home/with friends/other places) for a month or longer?

NO....................................... 1
YES..................................... 5

AD8 How old were you the (first/last) time you did any of these things? **REVIEW SX CODED 5 IN AD6.1-11**

AGE ONS:____/____
ONS: 1 5

1. **IF DK ONSET:** Were some of these difficulties happening before you were 7, for example first or second grade?

NO 1
YES……………………….5

AGE REC:____/____
REC: 1 5

IF AGE ONS WITHIN 2 YRS OF AGE REC, SKIP TO AD9.

A. Between (AGE ONS) and (AGE REC), was there any full year when you were not overly active, fidgety or impatient?

NO.. 1
YES....................................... 5

AD9 Did you or a parent ever talk to a doctor because of these problems?

NO.. 1
YES....................................... 5

AD10 Were you ever given any medicine to help you with the problems we've been talking about, such as (**REVIEW SX**)?

NO ....(SKIP TO OD1) 1
YES ....(SPECIFY)........... 5

ONS. How old were you when you first started taking the medicine(s)?

_______AGE

DRUG 1. _______________________________________ CODE: ___________

DRUG 2. _______________________________________ CODE: ___________

DRUG 3. _______________________________________ CODE: ___________

IF DK NAME OF MEDS, ASK: Do you think you took Ritalin, Adderall, Concerta, Dexadrine or Cylert?

NO………………………...1
YES....................................... 5
DK……………………..…9

A. Are you still taking the medicine?

NO.. 1
YES ....(SKIP TO C)........ 5

B. How old were you when you stopped taking the medicine(s)?

_______AGE

C. After you started taking the medicine, did these problems get better?

NO.. 1
YES....................................... 5

	I am going to ask you about periods when you may have done things that made people angry with you—like arguing with them, disobeying grownups, annoying other people on purpose, blaming others for your own mistakes, or being very crabby. I want to ask you about things that lasted six months or more. **BEGIN SCORING ASTERISKED ITEMS ON ODD TALLY SHEET.**	
ODD3RA1<br>ODD4A1<br>ODDICDG1-1	OD1 Did you ever have a period of six months or more when you lost your temper a lot?	NO……………………………1<br>YES…………………………..5*
ODD3RA2<br>ODD4A2<br>ODDICDG1-2	OD2 Did you ever go through a period when you argued a lot with your parents, your teachers, or other adults?	NO……………………………1<br>YES…………………………..5*
ODD3RA3<br>ODD4A3<br>ODDICDG1-3	OD3 Did you often just refuse to do things that your parents, teachers, or other adults asked, like taking out the garbage or doing your homework?	NO……………………………1<br>YES…………………………..5*
ODD3RA4<br>ODD4A4<br>ODDICDG1-4	OD4 Did you ever have a period when other people said that you were always doing things on purpose to annoy or bother them---for example, teasing them?	NO…...(SKIP TO OD5)……..1<br>YES…………………………..5
	A. Did you only do this with your brothers and sisters?	NO……………………………1*<br>YES…………………………..5
ODD3RA5<br>ODD4A5<br>ODDICDG1-5	OD5 Did you ever go through a period of six months or more when you blamed others a lot if you were caught doing something wrong or if something bad happened to you?	NO…...(SKIP TO OD6)……..1<br>YES…………………………..5
	A. Did you only do this with your brothers and sisters?	NO……………………………1*<br>YES…………………………..5
ODD3RA6<br>ODD4A6<br>ODDICDG1-6	OD6 Did you feel that people bugged you or that things annoyed you a lot?	NO…...(SKIP TO OD7)……..1<br>YES…………………………..5
	A. Did you only feel this way about your brothers and sisters?	NO……………………………1*<br>YES…………………………..5
ODD3RA7<br>ODD4A7<br>ODDICDG1-7	OD7 Did you ever go through a period when you got mad a lot because you felt others were being mean or unfair to you?	NO…....(SKIP TO OD8)…….1<br>YES…………………………..5
	A. Did you only feel this way about your brothers and sisters?	NO……………………………1*<br>YES…………………………..5
ODD3RA8<br>ODD4A8<br>ODDICDG1-8	OD8 When someone did something unfair to you, did you often try to get even with them? For example, telling other people things about them that weren't true or trying to get them in trouble with parents or teachers--did you do that kind of thing a lot?	NO…(SKIP TO BOX OD8)…1<br>YES…………………………..5
	A. Did you only do this with your brothers and sisters?	NO……………………………1*<br>YES…………………………..5
	**BOX OD8 IF 4 OR MORE *ITEMS IN OD1-OD8, CONTINUE OTHERS SKIP TO PT1.**	
ODD3RA9	OD9 OMITTED	

REVIEW SX ENDORSED IN OD1-OD8

OD10 Did these feelings or behaviors ever…

Variable	Item	NO	YES	NA
ODD4B	1. make your grades go down at school?	1	5	
	2. make your teachers angry with you or say that you had a bad attitude?	1	5	
	3. make your parents really angry with you?	1	5	
	4. make your parents ground you or punish you in some other way?	1	5	
	5. make your boss mad at you?	1	5	7
	6. make you lose friends?	1	5	
	7. make you sad or lonely?	1	5	

IF ALL CODED 1 IN OD10.1-7, THEN SKIP TO OD11.

A. Did having these feelings or behaviors get you into serious trouble for a month or longer?

NO……………………………1
YES…………………………..5

ODD3RA
ODD4A
ODDICDD

OD11 Now, I'd like you to think about the period of 6 months when you had the greatest number of the problems or experiences we've talked about. These experiences might not have occurred together. But they must have occurred in the same 6-month period.

During this period of 6 months when you had the greatest number of these behaviors that we just talked about (OD1-8), how many of them did you experience at the same time? [if 4 or more, continue. Other wise skip to next page HE section]

OD12 We were talking about the period of 6 months when you had the greatest number of these feelings or behaviors. How old were you when you first had a period of 6 months or longer when several of these things happened?

AGE ONS: ___/___

When did you last have a period of 6 months or longer when several of these things happened?

AGE REC:____/____

OD13 Earlier, you said that you sometimes felt (depressed/sad/empty/uninterested) and had some other problems at the same time. Did the feelings or behaviors we just talked about, like (REVIEW SX) happen ONLY when you were feeling (depressed/sad/empty/uninterested) and having some of those other problems?

NO……………………………1
YES…………………………..5

For this part of the interview, I'd like you to tell me the two people who play the major parent roles in your life at home. It could be your biological mother and father, a stepmother or stepfather, or another relative, such as a grandparent. Can you tell me who those two people are?

Biol Mom, Step Mom, Guardian Mom, Adoptive Mom| Biol Dad, Step Dad, Guardian Dad, Adopt Dad

IF HOME SITUATION HAS CHANGED OVER TIME, PARENT FIGURE(S) MUST HAVE LIVED IN THE HOME AT LEAST 6 MONTHS.

BOX HE1

	NO	YES
**IS A MOTHER FIGURE PRESENT FOR CODING?**	**1**	**5**
**IS A FATHER FIGURE PRESENT FOR CODING?**	**1**	**5**

MOTHER FIGURE MUST BE A FEMALE. FATHER FIGURE MUST BE A MALE. IF NO TO EITHER, OMIT THE QUESTIONS THAT PERTAIN TO THAT PARENT FIGURE. REMIND R, AS NEEDED, WHO THE MOTHER AND FATHER FIGURES ARE.

		MOTHER	FATHER
HE1A How long has your (MOTHER FIGURE / FATHER FIGURE) played a role in your life?	YEARS	___ ___	___ ___

IF ALL OF LIFE, CODE CHILD'S CURRENT AGE.

HE2-HE12 OMITTED.

HEm13 Does your (MOTHER FIGURE) work outside the home (that is, have a job other than being a homemaker)?

NO……………………………….1
YES……………………………...5

HEf13 Does your (FATHER FIGURE) work outside the home (that is, have a job other than being a homemaker)?

NO……………………………….1
YES……………………………...5

HE14 Is your (FATHER FIGURE) ever away from home a lot due to any of the following reasons? **READ OPTIONS:**

	NO	YES
1. WORK	1	5
2. MILITARY SERVICE	1	5
3. ILLNESS (E.G., HOSPITALIZED	1	5
4. IMPRISONMENT………………………………………………	1	5
5. MARITALSEPARATION/DIVORCE/DESERTION……..….	1	5
6. DRUG/ALCOHOL USE.…………………………………………..	1	5
7. ANOTHER REASON…………………………….……………	1	5

HE15 Is your (MOTHER FIGURE) ever away from home a lot due to any of the following reasons? **READ OPTIONS:**

	NO	YES
1. WORK	1	5
2. MILITARY SERVICE	1	5
3. ILLNESS (E.G., HOSPITALIZED.	1	5
4. IMPRISONMENT………………………………………………	1	5
5. MARITALSEPARATION/DIVORCE/DESERTION……….….	1	5
6. DRUG/ALCOHOL USE.……………………………………....….	1	5
7. ANOTHER REASON…………………………….……………..	1	5

HE16 OMITTED

HE17 A. Does your (MOTHER FIGURE/ FATHER FIGURE) help you with things like....

	MOTHER NO	MOTHER YES	FATHER NO	FATHER YES
1. SCHOOLWORK OR PROJECTS…………..	1	5	1	5
2. CHORES……….…..………………………..	1	5	1	5
3. FUN ACTIVITIES…………………………..	1	5	1	5
4. SHOPPING………………………………….	1	5	1	5
5. MAKING PLANS……………………………	1	5	1	5
6. ANYTHING ELSE…………………………..	1	5	1	5

B. Would you say that your (MOTHER FIGURE / FATHER FIGURE) spends time with you...

	MOTHER	FATHER
MORE THAN MOST PARENTS……………………..	1	1
SAME AS MOST PARENTS………………………..	2	2
LESS THAN MOST PARENTS…………………….	3	3

HE18A. Do you and your (MOTHER FIGURE / FATHER FIGURE) ever talk about the news or what is going on in the world?

	MOTHER	FATHER
NO……………….	1	1
YES……………...	5	5

B. Do you and your (MOTHER FIGURE / FATHER FIGURE) spend time talking about other things, like movies, your friends, or anything else?

	MOTHER	FATHER
NO……………….	1	1
YES……………...	5	5

HE19. Do you talk to your (MOTHER FIGURE / FATHER FIGURE) about your problems or when you are worried about something?

	MOTHER	FATHER
NO……………….	1	1
YES……………...	5	5

HE20A. How well do you get along with your (MOTHER FIGURE / FATHER FIGURE) most of the time?

	MOTHER	FATHER
POOR……………	1	1
FAIR……………..	2	2
GOOD……………	3	3
EXCELLENT……	4	4

B. How close do you feel to your (MOTHER FIGURE/ FATHER FIGURE)?

	MOTHER	FATHER
NOT AT ALL CLOSE……………………………….	1	1
SOMEWHAT CLOSE………………………………..	2	2
VERY CLOSE………………………………………...	3	3

HE21-HE23 OMITTED

BOX HE24 IF CHILD LIVES IN A SINGLE PARENT FAMILY AND PARENTS DO NOT SHARE CUSTODY, SKIP TO HE28. OTHERS CONTINUE.

HE24. Overall, how would you rate your (PARENT FIGURES') relationship with each other-- excellent, good, fair, or poor?

EXCELLENT 1
GOOD... 2
FAIR..3
POOR.. 4

HE25.	Some parents really enjoy being with each other while others don't. Do your (PARENT FIGURES) usually seem to enjoy each other?	NO…...……………………………1<br>YES………………………………5
HE26.	Do your (PARENT FIGURES) often argue or fight in front of you?	NO………………………………..1<br>YES………………………………5
HE27.	Do either of your (PARENT FIGURES) ever hit the other?	NO…….(SKIP TO HE28)…………1<br>YES………………………………5
A.	Do you ever see it happen?	NO………………………………..1<br>YES………………………………5
HE28.	How much arguing and tension is there in your household -- a lot, some, a little, or none?	A LOT……………………………1<br>SOME…………………………….2<br>A LITTLE………………………..3<br>NONE……………………………4

HE29A. When you do something that your (MOTHER FIGURE / FATHER FIGURE) thinks is wrong, does s/he yell or fuss at you…

	MOTHER	FATHER
MORE THAN MOST PARENTS…………………	1	1
SAME AS MOST PARENTS……………………..	2	2
LESS THAN MOST PARENTS……………….……..	3	3

AS29 B has been deleted.

HE30. When you do something wrong, does your (MOTHER FIGURE / FATHER FIGURE) ground you – that is, not let you do something you really want to do…

	MOTHER	FATHER
MORE THAN MOST KIDS…………………..……	1	1
SAME AS MOST KIDS……………………...….	2	2
LESS THAN MOST KIDS……………...….……...	3	3

HE31. Do you get into trouble with your (MOTHER FIGURE/ FATHER FIGURE)…

	MOTHER	FATHER
MORE THAN MOST KIDS…………………..……	1	1
SAME AS MOST KIDS……………………...….	2	2
LESS THAN MOST KIDS……………...….……...	3	3

HE32. OMITTED

HE33. Some parents are strict with their children. They have lots of rules or don't allow the children to do things that most of their friends can. Is your (MOTHER FIGURE / FATHER FIGURE) too strict, just about right, or not strict enough?

	MOTHER	FATHER
TOO STRICT	1	1
ABOUT RIGHT	2	2
NOT STRICT ENOUGH	3	3
NOT INVOLVED	4	4

HE34. When your parents make rules, how often does your (MOTHER FIGURE / FATHER FIGURE) follow through on them and enforce them?

	MOTHER	FATHER
MOST OF THE TIME	1	1
SOME OF THE TIME	2	2
NOT VERY OFTEN	3	3

HE35. OMITTED

HE36 Deleted Question

HE37 Which of the following descriptions fits your (PARENT FIGURES)?

A. My (PARENT FIGURES) know about my plans.
- ALWAYS 1
- USUALLY 2
- SOMETIMES 3
- RARELY 4

B. My (PARENT FIGURES) have a pretty good idea of my interests, activities, and whereabouts.
- ALWAYS 1
- USUALLY 2
- SOMETIMES 3
- RARELY 4

C. My (PARENT FIGURES) know where I am and who I am with when I am not at home.
- ALWAYS 1
- USUALLY 2
- SOMETIMES 3
- RARELY 4

HE38. What is the usual way in which your (MOTHER FIGURE / FATHER FIGURE) punishes you?

	MOTHER	FATHER
NON-PHYSICAL (SCOLD, ISOLATE, FINE, REMOVE PRIVILEGES)	1	1
PHYSICAL, MILD	2	2
PHYSICAL, HARSH	3	3
DOES NOT PUNISH	4	4

HE39 & HE40 OMITTED

HE41	A.	Do you have any difficulty making new friends?	NO……………………………….1<br>YES……………………………...5
	B.	Do you have any difficulty keeping friends?	NO……………………………….1<br>YES……………………………...5
HE42	A.	How many of your friends do your (PARENT FIGURES) know?	NONE OF THEM............................1<br>A FEW OF THEM...........................2<br>MOST OF THEM............................3<br>ALL OF THEM 4
	B.	How many of your friends do your (PARENT FIGURES) dislike?	NONE OF THEM............................1<br>A FEW OF THEM...........................2<br>MOST OF THEM............................3<br>ALL OF THEM 4

Now I'd like to ask a few questions about other people who may be important in your life.

HE43	A.	How many of your best friends smoke?	NONE OF THEM............................1<br>A FEW OF THEM...........................2<br>MOST OF THEM............................3<br>ALL OF THEM 4
	B.	How many of your best friends use alcohol?	NONE OF THEM............................1<br>A FEW OF THEM...........................2<br>MOST OF THEM............................3<br>ALL OF THEM 4
	C.	How many of your best friends use marijuana?	NONE OF THEM............................1<br>A FEW OF THEM...........................2<br>MOST OF THEM............................3<br>ALL OF THEM 4
	D.	How many of your best friends use other drugs (like cocaine, uppers, or any of the other drugs we've talked about)?	NONE OF THEM............................1<br>A FEW OF THEM...........................2<br>MOST OF THEM............................3<br>ALL OF THEM 4
HE44.		Have ever had a boyfriend or girlfriend with whom you were romantically involved?	NO………(SKIP TO HE45A)........1<br>YES 5
	A.	Have you had any boyfriends or girlfriends who smoked?	NO..1<br>YES 5
	B.	Have you had any boyfriends or girlfriends who used alcohol?	NO..1<br>YES 5
	C.	Have you had any boyfriends or girlfriends who used marijuana?	NO..1<br>YES 5
	D.	Have you had any boyfriends or girlfriends who used other drugs?	NO..1<br>YES 5

HE45 A.	How many of the kids you go to school with smoke?	NONE OF THEM............................1<br>A FEW OF THEM...........................2<br>MOST OF THEM............................3<br>ALL OF THEM...............................4
B.	How many of the kids you go to school with use alcohol?	NONE OF THEM............................1<br>A FEW OF THEM...........................2<br>MOST OF THEM............................3<br>ALL OF THEM...............................4
C.	How many of the kids you go to school with use marijuana?	NONE OF THEM............................1<br>A FEW OF THEM...........................2<br>MOST OF THEM............................3<br>ALL OF THEM...............................4
D.	How many of the kids you go to school with use other drugs (like cocaine, uppers, or any of the other drugs we've talked about)?	NONE OF THEM............................1<br>A FEW OF THEM...........................2<br>MOST OF THEM............................3<br>ALL OF THEM...............................4
HE46.	Do you have any sisters or brothers?<br>**BROTHERS & SISTERS INTERPRETED BROADLY. COUNT ANYONE <u>CONSIDERED</u> TO BE A SIBLING, EVEN IF NOT BIOLOGICALLY RELATED.**	NO……...(SKIP TO HE51)…….1<br>YES……………………………..5
A.	How well do you get along with your brother(s)/ sister(s) most of the time?	POOR ....1<br>FAIR ....2<br>GOOD..3<br>EXCELLENT 4
B.	How close do you feel to your brother(s)/ sister(s)?	NOT AT ALL CLOSE.....................1<br>SOMEWHAT CLOSE.....................2<br>VERY CLOSE.................................3
HE47 A.	Do you have any brother(s)/ sister(s) living at home who smoke?	NO..1<br>YES..5
B.	Do you have any brother(s)/ sister(s) living at home who use alcohol?	NO..1<br>YES..5
C.	Do you have any brother(s)/ sister(s) living at home who use marijuana?	NO..1<br>YES..5
D.	Do you have any brother(s)/ sister(s) living at home who use other drugs?	NO..1<br>YES..5
HE48 A.	Have any of your brother(s)/ sister(s) ever bought you or given you cigarettes?	NO..1<br>YES..5
B.	Have any of your brother(s)/ sister(s) ever bought you or given you alcohol?	NO..1<br>YES..5
C.	Have any of your brother(s)/ sister(s) ever bought you or given you marijuana?	NO..1<br>YES..5
D.	Have any of your brother(s)/ sister(s) ever bought you or given you other drugs?	NO..1<br>YES..5

HE49 & HE50 OMITTED

HE51. Do you or others think you look older than your age?	NO....1<br>YES....5
A. **IF R IS FEMALE, ASK:**<br>Have you had your first menstrual period?	NO....1<br>YES....5
**IF YES:** How old were you when you had your first menstrual period?	AGE ONS: __ __
**IF R IS MALE, ASK:**<br>Has your voice changed?	NO....1<br>YES....5
**IF YES:** How old were you when your voice first changed?	AGE ONS: __ __

I've asked you a lot of questions about your feelings, experiences, and behavior. Of course, people are not all the same, and maybe there is something that is important to you that I have missed. Is there anything else that you think I should know?

____

____

____

____

TYPE OF INTERVIEW:

IN PERSON……………………………………2
BY TELEPHONE……………………………..3
BY PROXY…………………………………….4

RATE ACCURACY OF CODES THROUGHOUT C-SSAGA-A-IV:

NO DIFFICULTY…………………………………………………………………………………..1
SOME PROBLEMS, BUT MOST RATINGS REASONABLY ACCURATE……….……………2
MAJOR DIFFICULTY IN CONDUCTING EXAM……………………………………………..3
IMPOSSIBLE TO RATE WITH ANY CONFIDENCE……………………………………………4
